# New N-acyl Thiourea Derivatives: Synthesis, Standardized Quantification Method and In Vitro Evaluation of Potential Biological Activities

**DOI:** 10.3390/antibiotics12050807

**Published:** 2023-04-25

**Authors:** Roxana Roman, Lucia Pintilie, Miron Teodor Căproiu, Florea Dumitrașcu, Diana Camelia Nuță, Irina Zarafu, Petre Ioniță, Mariana Carmen Chifiriuc, Cornel Chiriță, Alina Moroșan, Marcela Popa, Coralia Bleotu, Carmen Limban

**Affiliations:** 1 Department of Pharmaceutical Chemistry, Faculty of Pharmacy, “Carol Davila” University of Medicine and Pharmacy, 6 Traian Vuia, 020956 Bucharest, Romania; roxana.roman@drd.umfcd.ro (R.R.); diana.nuta@umfcd.ro (D.C.N.); carmen.limban@umfcd.ro (C.L.); 2 National Institute for Chemical-Pharmaceutical Research & Development, 112 Vitan Av., 031299 Bucharest, Romania; 3 “C. D. Nenitzescu” Institute of Organic and Supramolecular Chemistry, 202B Splaiul Independenței, 060023 Bucharest, Romania; dorucaproiu@gmail.com (M.T.C.); fdumitra@yahoo.com (F.D.); 4 Department of Organic Chemistry, Biochemistry and Catalysis, Faculty of Chemistry, University of Bucharest, 4-12 Regina Elisabeta, 030018 Bucharest, Romania; zarafuirina@yahoo.fr (I.Z.); p_ionita@yahoo.co.uk (P.I.); 5 Department of Microbiology, Faculty of Biology & Research Institute of the University of Bucharest (ICUB), University of Bucharest, 060101 Bucharest, Romania; carmen.chifiriuc@bio.unibuc.ro (M.C.C.); marcela.popa@bio.unibuc.ro (M.P.); cbleotu@yahoo.com (C.B.); 6 Romanian Academy, 010071 Bucharest, Romania; 7 Department of Pharmacology and Clinical Pharmacy, Faculty of Pharmacy, “Carol Davila” University of Medicine and Pharmacy, 6 Traian Vuia, 020956 Bucharest, Romania; cornel.chirita@umfcd.ro; 8 Department of Organic Chemistry “Costin Nenitescu”, Faculty of Applied Chemistry and Materials Science, University POLITEHNICA of Bucharest, 011061 Bucharest, Romania; alina.morosan@upb.ro; 9 Department of Celular and Molecular Pathology, Stefan S. Nicolau Institute of Virology, 285 Mihai Bravu Ave., 030304 Bucharest, Romania; 10 Academy of Romanian Scientists, Ilfov No. 3, 050044 Bucharest, Romania

**Keywords:** thiourea derivatives, benzamide, antimicrobial activity, antioxidant activity, synthesis, reversed phase HPLC, validation

## Abstract

New N-acyl thiourea derivatives with heterocyclic rings have been synthesized by first obtaining isothiocyanate, which further reacted with a heterocyclic amine, characterized by (FT-IR, NMR spectroscopy and FT-ICR) and tested for their in vitro antimicrobial, anti-biofilm and antioxidant activities to obtain a drug candidate in a lead-optimization process. From the tested compounds, those bearing benzothiazole (**1b**) and 6-methylpyridine (**1d**) moieties revealed anti-biofilm activity against *E. coli* ATCC 25922 at MBIC values of 625 µg/mL. Compound **1d** exhibited the highest antioxidant capacity (~43%) in the in vitro assay using 1,1-diphenyl-2-picrylhydrazyl (DPPH). Considering the in vitro results, the highest anti-biofilm and antioxidant activities were obtained for compound **1d**. Therefore, a reversed-phase high-performance liquid chromatography (RP-HPLC) method has been optimized and validated for the quantitative determination of compound **1d**. The detection and quantitation limits were 0.0174 μg/mL and 0.0521 μg/mL, respectively. The R^2^ correlation coefficient of the LOQ and linearity curves were greater than 0.99, over the concentration range of 0.05 μg/mL–40 μg/mL. The precision and accuracy of the analytical method were within 98–102%, confirming that the method is suitable for the quantitative determination of compound **1d** in routine quality control analyses. Evaluating the results, the promising potential of the new N-acyl thiourea derivatives bearing 6-methylpyridine moiety will be further investigated for developing agents with anti-biofilm and antioxidant activities.

## 1. Introduction

Thiourea derivatives have been reported in the literature for their multiple biological activities, including antibacterial [1,2,3,4], antifungal [5,6], antitubercular [7,8,9], anthelmintic [10], antiviral [11,12], anticancer [4,13,14], analgesic [15,16,17], anti-inflammatory [1,18], anti-diabetic [19,20], anticonvulsant [21], antithyroid [22,23], antioxidant [24], insecticidal [25], herbicidal [26], and rodenticidal [27] effects. Thiourea derivatives are also versatile intermediates for the synthesis of heterocyclic compounds such as 1,3-thiazoles, pyrimidines, 1,2,4-triazines and 1,3-quinazolines [28]. Merging acyl thioureas with carbonyl and thiocarbonyl groups in the same molecule led to physico-chemical and biological properties appropriate for application in coordination chemistry, as intermediates in organic synthesis for important heterocyclic scaffolds, and for analytical applications such as potentiometric sensors for heavy metals or chemosensory for anions [29], as well as in the agricultural and biomedical field. 

The potential of this scaffold as an excellent building block in the discovery of new anti-infective drug candidates has been reported in many studies. A series of N-acyl-substituted derivatives of the physiologically active alkaloid anabasine has proven moderate antibacterial and antifungal activities [6]. From a series of aspirin-bearing alkylated amines, the [2-[(3-methylphenyl)carbamothioylcarbamoyl]phenyl] acetate proved to be the most active against *Escherichia coli* and *Staphylococcus aureus* [30]. The 1,4-bis(decoxyphenyl)carbamothioyl-terephthalamide derivatives with an alkyl chain of different lengths (with 10, 12, and 14 carbon atoms) have shown antibacterial activity against *E. coli* ATCC 25922, the antibacterial activity decreasing with the increase of carbon chain. The molecular docking interaction study revealed the binding affinity of these compounds to the active site of enzyme enoyl ACP reductase (FabI) of *E. coli* [2]. A series of novel thiouracil derivatives having an acyl thiourea moiety has been developed by structural transformation of a lead SecA inhibitor, showing antibacterial activities against Gram-positive bacterial strains (*Bacillus amyloliquefaciens*, *Staphylococcus aureus* and *Bacillus subtilis*) [31]. 

Some pyrazole acyl thiourea derivatives have also shown in vitro fungicidal activity against *Gibberella zeae*, *Fusarium oxysporum*, *Cytospora mandshurica* and *Tobacco mosaic* virus [32], while N-((substituted-phenyl)carbamothioyl)-1,3-dimethyl-1*H*-pyrazole-4-carboxamides proved to be active against the *Botryospuaeria berengeriana* fungal pathogen [33]. 

Considering the global threat of antimicrobial resistance and the lack of novel anti-infective drugs, developing novel antimicrobial agents remains one of the main goals in medicinal chemistry. Given these considerations and to continue our efforts to develop novel antimicrobial agents as bioactive lead candidates, we designed [34] and synthesized thiourea derivatives carrying a heterocyclic nucleus (**1a**–**1g**).

Our strategy to design and synthesize some novel prototypes has been based on bringing together two pharmacophores, i.e., thiourea and a heterocyclic ring (thiazole, benzothiazole, pyridine and pyrimidine) in a single molecular backbone. 

The benzo[d]thiazole-based N-acyl thiourea derivatives (**1a**–**1g**) were designed and synthesized according to a previously published method, with punctual modifications [34,35], characterized by in silico and experimental approaches and evaluated for their biological properties. Furthermore, a routine quality control method for quantitative evaluation of the most promising compound (**1d**) has been developed and validated by reversed-phase high-performance liquid chromatography (RP-HPLC).

## 2. Results

### 2.1. Chemistry

**Scheme 1:**
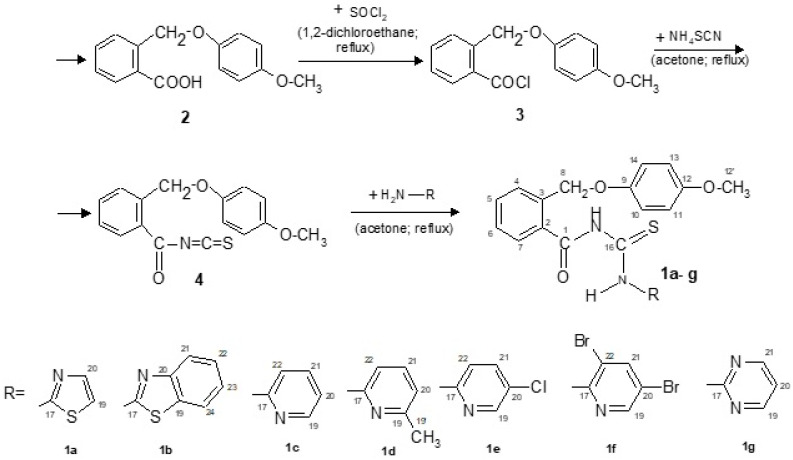
Synthesis scheme of new N-acyl thiourea derivatives (**1a**–**1g**).

The synthesis (Scheme 1) of the new N-acyl thiourea derivatives (**1a**–**1g**) involves the condensation of the acid chloride (**3**) with ammonium thiocyanate in anhydrous acetone, followed by the reaction of the resulting isothiocyanate (**4**) with a heterocyclic amine. The reaction proceeds by nucleophilic addition of the amine to the isothiocyanate.

Isothiocyanates are key intermediaries in the synthesis of nitrogen, sulfur and oxygen compounds. The high electrophilicity and nucleophilicity associated with the carbon and sulfur atoms of the isothiocyanates and their extended π electron system make them important precursors of target molecules.

#### Optimization Study for the Synthesis of **1b**

Since the synthesis yield of compound **1b** was low, we optimized its synthesis by using a phase-transfer catalyst, the tetra-*n*-butylammonium bromide (TBAB), and observed how the catalyst influenced the reaction speed and its yield.

Using a phase-transfer catalyst to stir a heterogeneous reaction system is gaining importance in the search to improve the methods of obtaining acyl thiourea derivatives by reacting isothiocyanates with nucleophiles. For example, Acyl isothiocyanates are obtained in good yields by treating the acid chlorides with potassium or ammonium thiocyanate, using TBAB as a phase-transfer catalyst [36,37,38,39,40,41,42]. Indeed, in our study, using TBAB as a phase-transfer catalyst, the yield improved to 76% relative to 2-((4-methoxyphenoxy)methyl)benzoyl chloride, compared to the 41% reaction yield carried out without the catalyst.

### 2.2. Spectral Data

The structures of the synthesized compounds were determined based on Fourier Transform InfraRed (FTIR) spectroscopy and Nuclear Magnetic Resonance (NMR) spectroscopy techniques.

#### 2.2.1. 2-((4-Methoxyphenoxy)methyl)-N-(thiazol-2-ylcarbamothioyl)benzamide (**1a**) (C_19_H_17_N_3_O_3_S_2_; MW = 399.48 g/mol; m.p. 128–131 °C; yield 65%)

^1^H-NMR (300 MHz, DMSO-d6, δ ppm, J Hz): 7.71 (dd, ^1^H, H-7, 1.4, 7.5); 7.65 (dd, ^1^H, H-4, 1.8, 7.5); 7.59 (td, ^1^H, H-5, 7.5, 1.4); 7.55 (d, ^1^H, H-20, 3.6); 7.50 (td, ^1^H, H-6, 7.5, 1.8); 7.29 (d, ^1^H, H-19, 3.6); 6.86 (d, 2H, H-11, H-13, 9.2); 6.82 (d, 2H, H-10, H-14, 9.2); 5.27 (s, 2H, H-8); 3.69(s, 3H, H-12′).

NH protons give a very broad signal in the 13.5–11.0 ppm area.

^13^C-NMR (75 MHz, DMSO-d6, δ ppm): 179.30 (C-16); 169.09 (C-1); 167.80 (C-17); 154.56 (C-12); 153.19 (C-9); 138.58 (C-20); 137.20 (C-3); 134.15 (C-2); 132.4 (C-4, C-5, C-7); 129.39 (C-4, C-5, C-7); 129.22 (C-4, C-5, C-7); 128.78 (C-6); 116.83 (C-11, C-13); 115.53 (C-10, C-14); 114.70 (C-19); 68.99 (C-8); 56.31 (C-12’).

FT-IR: 2347.9 w; 1623.8 w; 1546.6 w; 1509.0 vs; 1 485.9 s; 1454.1 m; 1393.3 s; 1326.8 s; 1276.7 w; 1223.6 s; 1181.2 m; 1043.3 m; 1018.2 m; 925.7 w; 817.7 s; 768.5 s; 737.6 s; 694.3 w; 603.6 w.

#### 2.2.2. N-(benzo[d]thiazol-2-ylcarbamothioyl)-2-((4-methoxyphenoxy)methyl)benzamide (**1b**) (C_23_H_19_N_3_O_3_S_2_; MW = 449.53 g/mol; m.p. 169–172 °C; yield 41%, after optimization 76%)

^1^H-NMR (300 MHz, DMSO-d6, δ ppm, J Hz): 8.08 (bd, ^1^H, H-24 or H-21, 7.4); 7.83 (bd, ^1^H, H-21 or H-24, 7.8); 7.70 (bd, ^1^H, H-7, 7.4); 7.65–7.49 (m, 4H, H-4, H-5, H-6, H-22); 7.43 (td, ^1^H, H-23, 8.1, 1.2); 6.92 (d, 2H, H-11, H-13, 9.2); 6.78 (d, 2H, H-10, H-14, 9.2); 5.29 (s, 2H, H-8); 3.60 (s, 3H, H-12′).

NH protons give a very broad signal in the 13.5–11.0 ppm area.

^13^C-NMR (75 MHz, DMSO-d6, δ ppm): 178.44 (C-16); 171.59 (C-1); 165.84 (C-17); 160.02 (C-20); 154.61 (C-12); 153.08 (C-9); 137.13 (C-3); 136.13 (C-19); 133.79 (C-2); 132.34 (C-4, C-5, C-7); 129.62 (C-4, C-5, C-7); 129.42 (C-4, C-5, C-7); 128.75 (C-6); 127.58 (C-22); 125.46 (C-24); 122.88 (C-23); 116.80 (C-21); 116.56 (C-10, C-14); 115.51 (C-11, C-13); 68.95 (C-8); 56.20 (C-12′).

FT-IR: 3225.1 s; 3099.2 m; 3078.5 m; 2923.1 m; 1753.8 vs; 1676.3 vs; 1657.2 s; 1592.4 w; 1571.1 m; 1489.6 w; 1483.7 s; 1412.9 m; 1391.2 w; 1292.4 s; 1245.5 m; 1075.1 m; 1056.3 w; 773.6 w; 754.4 m; 527.7 w; 503.5 w.

#### 2.2.3. 2-((4-Methoxyphenoxy)methyl)-N-(pyridin-2-ylcarbamothioyl)benzamide (**1c**) (C_21_H_19_N_3_O_3_S; MW = 393.446 g/mol; m.p. 110–114 °C; yield 56%)

^1^H-NMR (300 MHz, DMSO-d6, δ ppm, J Hz): 8.42 (bs, ^1^H, H-19); 7.94 (td, ^1^H, H-21, 7.6, 1.9); 7.67 (dd, ^1^H, H-7, 1.4, 7.4); 7.64–7.48 (m, 4H, H-4, H-5, H-6, H-22); 7.30 (dd, ^1^H, H-20, 5.9, 7.6); 6.92 (d, 2H, H-11, H-13, 9.2); 6.80 (d, 2H, H-10, H-14, 9.2); 5.27 (s, 2H, H-8); 3.66 (s, 3H, H-12′).

NH protons give a very broad signal in the 13.5–11.0 ppm area. The H-19 signal should be a doublet of doublets, the most unscreened ~8.30 ppm; probably due to reduced mobility, it appears as a very broad signal.

^13^C-NMR (75 MHz, DMSO-d6, δ ppm): 178.66 (C-16); 171.35 (C-1); 154.60 (C-12); 153.09 (C-9); 152.07 (C-17); 136.92 (C-3); 134.27 (C-2); 149.48 (C-19); 139.06 (C-21); 132.15 (C-4, C-5, C-7); 129.53 (C-4, C-5, C-7); 129.52 (C-4, C-5, C-7); 128.83 (C-6); 122.41 (C-20); 116.67 (C-10, C-14); 115.64 (C-22); 115.49 (C-11, C-13); 69.04 (C-8); 56.25 (C-12′).

FT-IR: 2993.0 w; 2325.7 w; 2080.8 w; 1621.8 w; 1545.7 w; 1509.0 vs; 1484.9 s; 1452.1 m; 1388.5 vs; 1323.9 s; 1277.6 m; 1262.2 m; 1220.7 vs; 1184.1 s; 1143.6 m; 1105.0 m; 1042.3 m; 1016.3 s; 923.7 s; 870.7 w; 818.6 m; 768.5 m; 739.6 vs; 694.3 m; 645.1 w; 603.6 w; 558.3 w; 512.0 w; 469.6 m; 429.1 m.

#### 2.2.4. 2-((4-Methoxyphenoxy)methyl)-N-((6-methylpyridin-2-yl)carbamothioyl)benzamide (**1d**) (C_22_H_21_N_3_O_3_S; MW = 407.476 g/mol; m.p. 95–98 °C; yield 52%)

^1^H-NMR (300 MHz, DMSO-d6, δ ppm, *J* Hz): 7.81 (t, ^1^H, H-21, 8.2); 7.69 (bd, ^1^H, H-7, 7.4); 7.63–7.51 (m, 4H, H-4, H-5, H-6, H-22); 7.13 (bd, ^1^H, H-20, 7.8); 6.91 (d, 2H, H-11, H-13, 9.2); 6.80 (d, 2H, H-10, H-14, 9.2); 5.27 (s, 2H, H-8); 3.66 (s, 3H, H-12′); 2.50 (s, 3H, H-19′).

NH protons give a very broad signal in the 13.5–11.0 ppm area.

^13^C-NMR (75 MHz, DMSO-d6, δ ppm): 178.59 (C-16); 170.47 (C-1); 157.75 (C-19); 155.01 (C-12); 153.36 (C-9); 151.79 (C-17); 137.02 (C-3); 134.69 (C-2); 139.31 (C-21); 132.06 (C-4, C-5, C-7); 129.64 (C-4, C-5, C-7); 129.21 (C-4, C-5, C-7); 128.79 (C-6); 121.21 (C-20); 113.29 (C-22); 117.17 (C-10, C-14); 115.83 (C-11, C-13); 69.45 (C-8); 56.55 (C-12′); 24.35 (C-19′).

FT-IR: 2961.2 w; 1604.5 w; 1556.3 w; 1531.2 s; 1509.0 vs; 1493.6 s; 1461.8 m; 1384.6 s; 1324.9 s; 1261.2 m; 1237.1 vs; 1164.8 m; 1095.4 s; 1025.9 s; 904.5 w; 812.9 vs; 795.5 vs; 757.9 s; 734.8 s.

#### 2.2.5. N-((5-chloropyridin-2-yl)carbamothioyl)-2-((4-methoxyphenoxy)methyl)benzamide (**1e**) (C_21_H_18_ClN_3_O_3_S; MW = 427.893 g/mol; m.p. 131–135 °C; yield 73%)

^1^H-NMR (300 MHz, DMSO-d6, δ ppm, *J* Hz): 8.75 (bs, 1H, H-22); 8.49 (d, ^1^H, H-19, 2.5); 8.06 (dd, ^1^H, H-21, 2.5, 8.9); 7.65 (dd, ^1^H, H-7, 1.4, 7.4); 7.63–7.47 (m, 3H, H-4, H-5, H-6); 6.91 (d, 2H, H-11, H-13, 9.2); 6.80 (d, 2H, H-10, H-14, 9.2); 5.20 (s, 2H, H-8); 3.72 (s, 3H, H-12′).

NH protons give a very broad signal in the 13.5–11.0 ppm area. The H-22 signal should be a more screened doublet, ~7.40 ppm; probably due to reduced mobility, it appears as a very broad singlet.

^13^C-NMR (75 MHz, DMSO-d6, δ ppm): 178.81 (C-16); 171.28 (C-1); 154.64 (C-12); 153.10 (C-9); 150.67 (C-17); 147.79 (C-19); 138.67 (C-21); 136.93 (C-3); 134.15 (C-2); 132.17 (C-4, C-5, C-7); 129.50 (C-4, C-5, C-7); 129.53 (C-4, C-5, C-7); 128.80 (C-6); 128.15 (C-20); 117.49 (C-22); 116.64 (C-10, C-14); 115.51 (C-11, C-13); 69.06 (C-8); 56.27 (C-12′).

FT-IR: 3227.3 w; 1723.1 m; 1704.8 vs; 1677.8 m; 1573.6 s; 1545.7 vs; 1508.1 vs; 1463.7 s; 1438.6 m; 1376.9 s; 1291.1 m; 1231.3 vs; 1205.3 s; 1144.6 m; 1117.6 m; 1043.3 s; 1030.8 s; 1009.6 m; 918.9 w; 872.6 w; 839.9 m; 810.9 s; 780.1 w; 746.3 vs; 727.0 vs; 695.2 vs; 673.0 m; 646.0 w.

#### 2.2.6. N-((3,5-dibromopyridin-2-yl)carbamothioyl)-2-((4-methoxyphenoxy)methyl)benzamide (**1f**) (C_21_H_17_Br_2_N_3_O_3_S; MW = 551.25 g/mol; m.p. 156–159 °C; yield 63%)

^1^H-NMR (300 MHz, DMSO-d6, δ ppm, *J* Hz): 8.68 (d, ^1^H, H-19, 2.2); 8.57 (d, ^1^H, H-21, 2.2); 7.65 (bd, ^1^H, H-7, 7.4); 7.62–7.47 (m, 3H, H-4, H-5, H-6); 6.97 (d, 2H, H-11, H-13, 9.2); 6.87 (d, 2H, H-10, H-14, 9.2); 5.26 (s, 2H, H-8); 3.71 (s, 3H, H-12′).

NH protons give a very broad signal in the 13.5–11.0 ppm area.

^13^C-NMR (75 MHz, DMSO-d6, δ ppm): 181.23 (C-16); 170.84 (C-1); 154.70 (C-12); 153.15 (C-9); 149.94 (C-17); 149.38 (C-19); 144.51 (C-21); 136.84 (C-3); 134.14 (C-2); 132.17 (C-4, C-5, C-7); 129.75 (C-4, C-5, C-7); 129.56 (C-4, C-5, C-7); 128.86 (C-6); 120.35 (C-22 or C-20); 119.53 (C-20 or C-22); 116.91 (C-10, C-14); 115.61 (C-11, C-13); 69.08 (C-8); 56.37 (C-12’).

FT-IR: 3068.2 w; 2962.1 w; 2087.6 w; 1599.7 w; 1507.1 vs; 1474.3 s; 1451.2 s; 1413.6 s; 1360.5 s; 1312.3 vs; 1264.1 vs; 1232.3 vs; 1194.7 s; 1098.3 vs; 1020.2 vs; 922.8 s; 858.2 s; 816.7 vs; 799.4 vs; 741.5 vs; 696.2 s; 632.5 w. 

#### 2.2.7. 2-((4-Methoxyphenoxy)methyl)-N-(pyrimidin-2-ylcarbamothioyl)benzamide (**1g**) (C_20_H_18_N_4_O_3_S; MW = 394.436 g/mol; m.p. 157–160 °C; yield 54%)

^1^H-NMR (300 MHz, DMSO-d6, δ ppm, *J* Hz): 8.74 (d, 2H, H-19, H-21, 4.9); 7.73 (dd, ^1^H, H-7, 1.4, 7.4); 7.65 (dd, ^1^H, H-4, 1.4, 7.4); 7.62 (td, ^1^H, H-5, 7.6, 1.3); 7.53 (td, ^1^H, H-6, 7.5, 1.4); 7.31 (t, ^1^H, H-20, 4.9); 6.92 (d, 2H, H-11, H-13, 9.2); 6.82 (d, 2H, H-10, H-14, 9.2); 5.27 (s, 2H, H-8); 3.68 (s, 3H, H-12′). 

NH protons give a very broad signal in the 13.5–11.0 ppm area.

^13^C-NMR (75 MHz, DMSO-d6, δ ppm): 179.06 (C-16); 169.20 (C-1); 159.40 (C-19, C-21); 158.05 (C-17); 154.61 (C-12); 153.09 (C-9); 137.34 (C-3); 134.57 (C-2); 132.26 (C-5); 129.61 (C-4); 129.07 (C-7); 128.48 (C-6); 118.64 (C-20); 116.77 (C-10, C-14); 115.53 (C-11, C-13); 68.87 (C-8); 56.29 (C-12′).

FT-IR: 3061.4 w; 2910.1 w; 2835.8 w; 1601.6 w; 1527.4 s; 1504.2 s; 1482.0 s; 1459.9 s; 1371.1 s; 1331.6 s; 1285.3 w; 1256.4 m; 1226.5 vs; 1185.0 s; 1105.0 m; 1024.0 s; 935.3 m; 860.1 w; 822.5 m; 786.8 m; 750.2 vs.

In addition, while performing the FT-ICR high-resolution mass spectrometry analysis on the tested compounds, for every peak revealed on the spectrum, the molecular formulae have been determined based on their *m*/*z*: **1a** (366.09 *m*/*z*), **1c** (392.11 *m*/*z*), **1d** (406.12 *m*/*z*), **1e** (426.07 *m*/*z*), **1f** (547.93 *m*/*z*), **1g** (393.10 *m*/*z*). 

Moreover, decomposition fragments of some of the chemicals have also been revealed. For **1a**: C_3_H_3_N_2_S (100.01 *m*/*z*), C_15_H_15_NO_3_ (258.11 *m*/*z*); **1d**: C_6_H_7_N_2_ (108.07 *m*/*z*), C_15_H_15_NO_3_ (258.11 *m*/*z*); **1e**: C_15_H_15_NO_3_ (258.11 *m*/*z*). 

### 2.3. Antimicrobial Activity Results

The in vitro antimicrobial testing results are presented in Table 1. The minimum inhibitory concentration (MIC) values obtained for the tested compounds were relatively high, ranging from >5000 to 1250 µg/mL, as compared to the antibiotic (ciprofloxacin) used as a positive control (MIC values of 0.012–0.62 µg/mL). 

Concerning the anti-biofilm effects, the results indicated minimum biofilm inhibitory concentration (MBIC) values between >5000 and 625 µg/mL. The compounds **1b** and **1d** exhibited the best anti-biofilm activity against *E. coli* ATCC 25922 at MBIC values of 625 µg/mL (Table 2).

**Figure 1:**
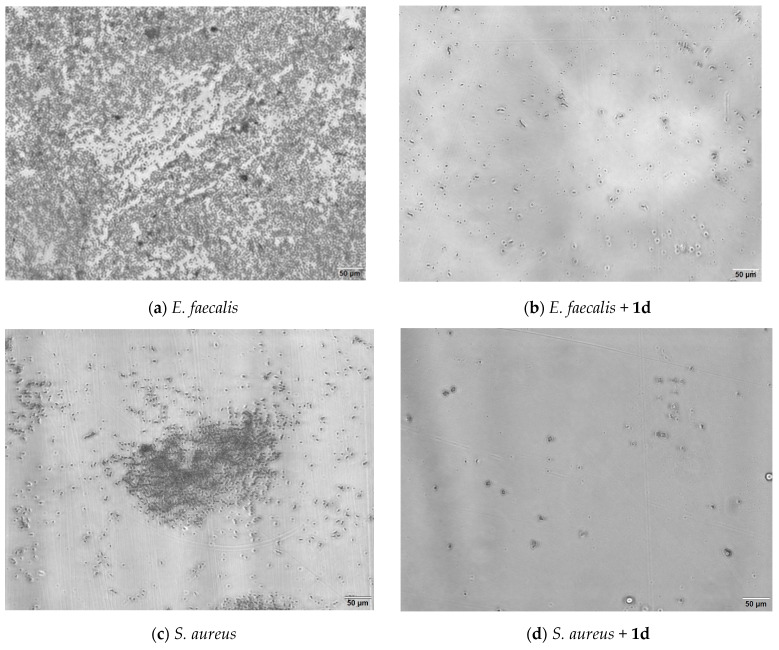

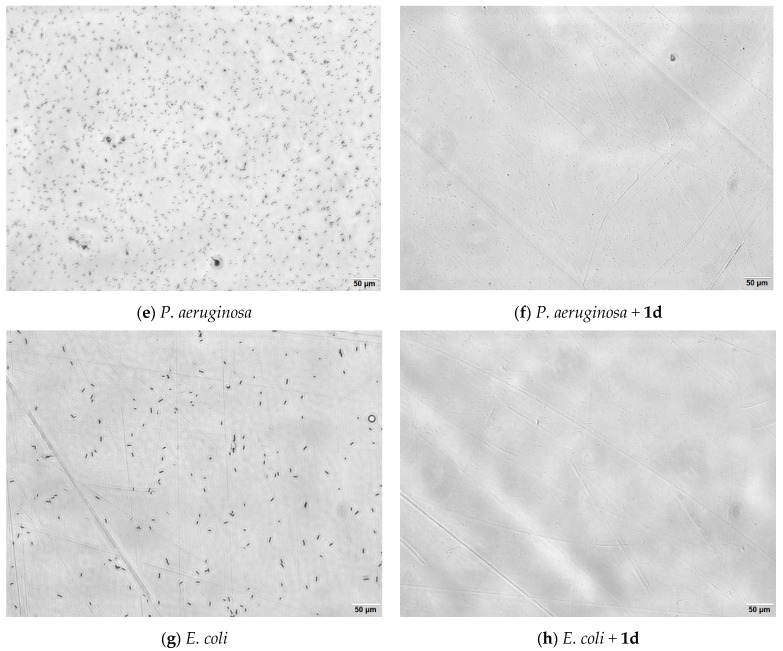
Inverted microscopy images of the bacterial biofilms developed on the plastic substratum in the absence (**a**,**c**,**e**,**g**) and presence (**b**,**d**,**f**,**h**) of the most active compound, **1d** (Olympus IX73 microscope).

The inhibitory effect of the biofilm development has also been demonstrated by the microscopic examination of the microbial biofilm in the presence/absence of the tested compounds, as exemplified in Figure 1 for the most active compound, **1d** (Figure 1).

### 2.4. Total Antioxidant Activity

The total antioxidant capacity was evaluated using the well-known DPPH assay, and the results are compiled in Table 3. For the compounds, **1a**–**1g**, the total antioxidant capacity (TAC) values showed that the highest antioxidant capacity is recorded for compound **1d** (~43%), followed by **1f** (~25%), while for the other compounds, the TAC values were between 10–15%.

### 2.5. Cytotoxicity Assay

The fluorescein diacetate dye (FDA) method that evaluates membrane integrity and intracellular enzyme activity as indicators of cell viability suggested that only a small proportion of the tested cells eliminated the dye after treatment with 250 µg/mL N-acyl thiourea derivatives.

**Figure 2:**
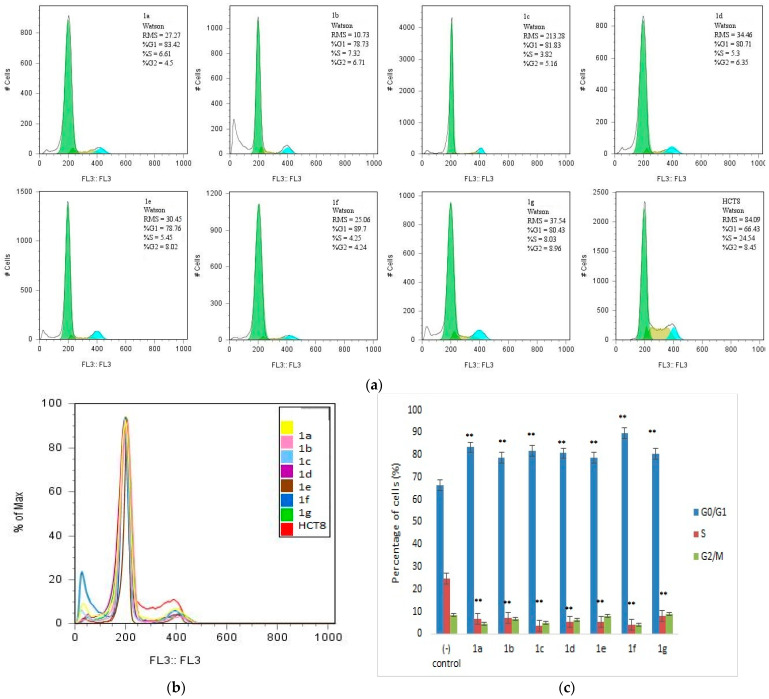
Flow cytometric analysis performed for the cell cycle distribution of HCT-8 cells treated with the N-acyl thiourea derivatives for 48 h. (**a**) Representative flow cytometry graph for each treated or untreated cell; (**b**) Overlayed histograms; (**c**) Percentage of cells in different cell cycle phases (** *p* < 0.005 as compared with the control group).

After 48 h of treatment, the HCT-8 cells were harvested to examine the effects on the cell cycle by flow cytometry. The results indicate that the N-acyl thiourea derivatives induced the G0/G1 phase arrest of the tested cells, and, as shown in Figure 2, the compounds caused a comparable decrease in the S phase. The results are consistent and statistically significant. In addition, the percentage of cells in the G2/M phase was slightly decreased by the **1a**, **1b**, **1c**, **1d**, and **1f** compounds compared with the control. 

Flow cytometry analysis showed that the newly tested compounds could cause the G0/G1 phase arrest after a prolonged contact time (Figure 2).

### 2.6. Validation of Quantitative Analysis Method by HPLC

#### 2.6.1. Specificity

System suitability was verified by registering three consecutive chromatograms of 30 µg/mL **1d** solution in the HPLC system. The symmetry factor (tailing factor) and theoretical plates were determined. The symmetry factor was demonstrated to be within the range of 0.8–1.8 (recorded values: 1.07–1.09), and the theoretical plates registered values over 3000. Mean, RSD (%) on retention time and areas of the main peak were calculated and proved to be not more than 2% (0.17% for the areas of the peaks and 0.08% for the retention time of the peaks), demonstrating the system precision. 

**Figure 3:**
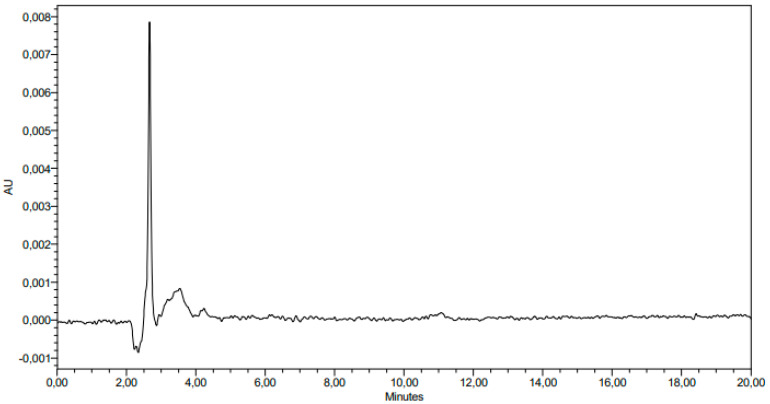
Chromatogram registered with the solvent used for sample preparation (methanol).

**Figure 4:**
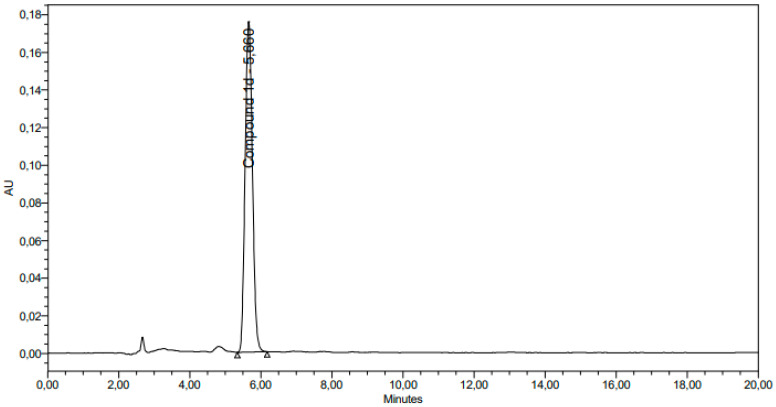
Chromatogram registered with sample solution (30 µg/mL compound **1d**).

For the interference study, the solvent used for sample preparation and solutions of 30 µg/mL **1d** were recorded and analyzed. The solvent chromatograms recorded no interfering peaks (Figure 3 and Figure 4).

Peak purity angles for **1d** recorded in the test solution chromatograms are lower than corresponding thresholds (for sample 1: 0.081 < 0.279; sample 2: 0.080 < 0.280), indicating that the method is well suited for proper separation. 

The results suggest that the chosen chromatographic parameters are adequate, and the method is specific for the quantitative determination of compound **1d**.

#### 2.6.2. LOD and LOQ

**Figure 5:**
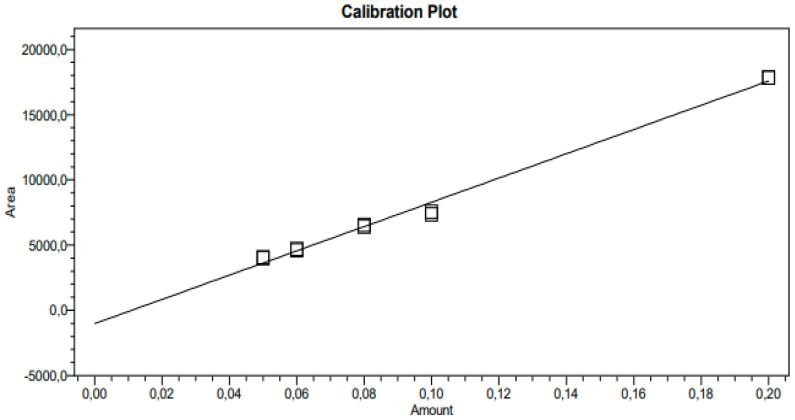
LOD/LOQ Curve (amount in µg/mL), where slope (b) = 93,068.63, standard deviation = 490.32, R^2^ = 0.9923. The calibration plot is extracted from HPLC Empower Software (extensive chromatograms are attached in the Supplementary Materials section).

**Figure 6:**
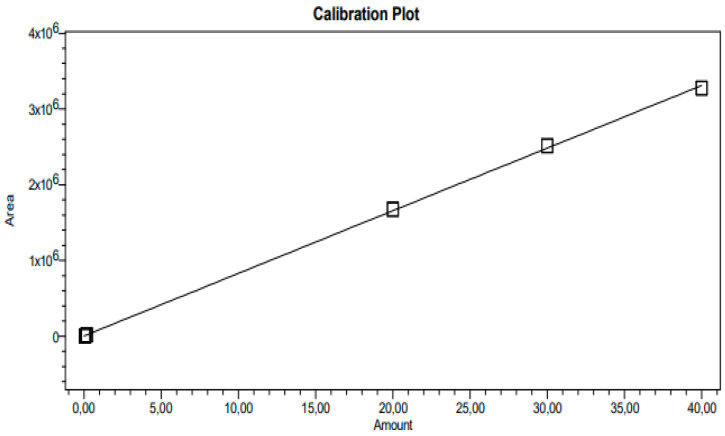
Linearity curve (amount in µg/mL) where slope (b) = 82,653.87, standard deviation = 19,721.72, R^2^ = 0.9998. The calibration plot is extracted from HPLC Empower Software (extensive chromatograms are attached in Supplementary Materials section).

The linear relationship was obtained between the area under peaks and their corresponding concentrations, with R^2^ = 0.9923 for LOD/LOQ parameter (Table 4 and Figure 5) and R^2^ = 0.9998 for the linearity parameter (R^2^ ≥ 0.99) (Table 5 and Figure 6). The formula describes the linear regression equation: Y = bX + m, where b is the estimated slope and m is the intercept. The limit of detection (LOD) and limit of quantitation (LOQ) values were determined based on the formula: LOD = 3.3* × σ/b  and LOQ = 10* σ/b, where σ is the standard deviation (see Materials and Methods section). In our case, b (slope) was 93,068.63, and the standard deviation was 490.32. LOD and LOQ values were calculated accordingly, and the obtained results are 0.0017 µg/mL (0.0057%) for LOD and 0.053 µg/mL (0.0177%) for LOQ. 

#### 2.6.3. Precision Results 

The intra-day and inter-day precision were carried out by performing six replicates of three different concentrations (20 µg/mL **1d** solution—Table 6, 30 µg/mL **1d** solution—Table 7, 40 µg/mL **1d** solution—Table 8) in two different days and evaluated in terms of RSD (%) calculated for peak areas. As a result, the intra-day (Precisions 1 and 2, individually) and inter-day precision (evaluation of cumulative Precisions 1 and 2) were shown to be less than 2%, confirming the method’s precision. 

#### 2.6.4. Accuracy Results

The accuracy of the method was demonstrated by the recovery procedure. The experiment was carried out by determining the recovery on a range of three concentrations (20 µg/mL, 30 µg/mL and 40 µg/mL solution **1d**). The sample solutions were prepared six times, on two different days, with a mean percent recovery of 100.21 ± 0.32 (99.89–100.53) in the first determination (Table 9) and 100.00 ± 0.47 (99.53–100.47) in the second determination (Table 10).

The recoveries obtained in each intra-day experiment confirm the accuracy of the method. Furthermore, for every validation parameter, the statistical analysis demonstrated that the RP-HPLC analytical method could be used for its intended purpose as an analytical tool in the identification and quantification of compound **1d**.

## 3. Discussion

There is international concern that the emergence and spread of resistance to all currently available antibiotics correlated with the slow discovery rate of novel antimicrobials will lead to a “post-antibiotic era” where common infections could become chronic or fatal and many medical procedures, such as surgery, invasive diagnosis techniques, oncological treatments will no longer be possible due to the fatal risk of infections [43]. In this context, the development of new antimicrobials is urgently required.

The present study aimed to continue the hypothesis traced in the previous research paper [34], regarding the antimicrobial activity of a series of thiourea derivatives with heterocyclic nuclei (thiazole, benzothiazole, pyridine and pyrimidine). The evaluated compounds, **1a**–**1g**, are referred to in the cited source as A.01–A.07. 

A series of molecular descriptors, i.e., area, volume, polar surface area (PSA), ovality, polarizability, permeability coefficient (log P), number of hydrogen donors, and number of hydrogen acceptors were calculated to determine the “drugability” of the evaluated chemicals. For compounds **1b** and **1f**, two Lipinski violations were detected [44]. 

The molecular docking studies predicted an optimized conformation of ligands in their target receptor proteins (*S. aureus* DNA gyrase B and *E. coli* DNA gyrase B), forming stable complexes. As an indicator of stability, compound **1a** (A.01) formed a hydrogen bond with amino acid moiety GLY101, similar to the hydrogen bond created by co-crystallized natural ligand RLIA301 (*E. coli* DNA gyrase B). In addition, compounds **1a** and **1b** registered the highest scores in the series when docked with *S. aureus* DNA gyrase B and *E. coli* DNA gyrase B, respectively. 

The molecular descriptors, good docking scores, and H-bonds created between ligands and interaction groups were correlated with the potential inhibitory activity against *Staphylococcus aureus* and *Escherichia coli*. Consequently, the in-silico data provided means to explore the in vitro affinity of the designed molecules to the selected proteins.

**Scheme 2:**
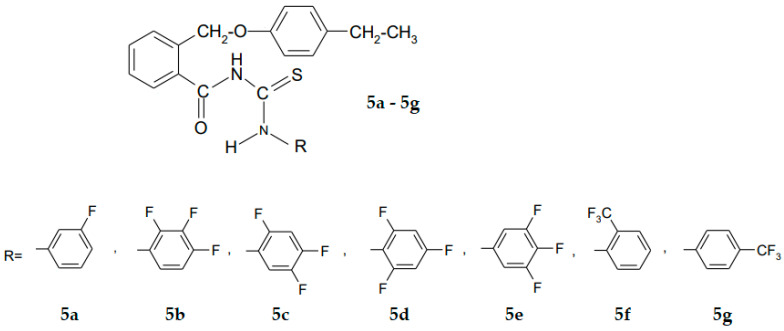
New 2-((4-ethylphenoxy)methyl)-N-(fluoro/trifluoromethylphenylcarbamothioyl)benzamides with enhanced antimicrobial activity.

In our intention to optimize the activity of acyl thiourea scaffold, the substitution of a fluorine atom or trifluoromethyl group on a phenyl nucleus was evaluated in a preceding study [45]. As a result, the molecular properties and docking scores processed with *E. coli* DNA gyrase B were developed, with favorable docking scores that indicate a possible inhibition of DNA replication. To evaluate the predicted antimicrobial activity, we synthesized new 2-((4-ethylphenoxy)methyl)-N-(fluoro/trifluoromethylphenylcarbamothioyl)benzamides, referred to in the mentioned source as **5a**–**5g** (Scheme 2). The highest antimicrobial activity against *E. coli*, *P. aeruginosa* and *E. faecalis* was obtained for the compounds designed with fluorine atoms (**5a**, **5f**, **5g**), the intensity increasing with the presence of the trifluoromethyl substituent in para position on the phenyl ring (**5g**). On the other hand, the compounds bearing three fluorine atoms exhibited the most potent antifungal effect in the series.

For many decades, the thiazole moiety has been an essential heterocycle in the world of chemistry, harboring multiple reactive positions for various reactions such as donor–acceptor, nucleophilic and oxidation, that could modulate biochemical pathways, enzymatic or receptor activities in biological systems [46]. Therefore, thiazole-bearing compounds show promising potential in medicinal chemistry for the development of analgesic, anti-inflammatory, antiviral, antidiabetic, antitumor, and antimicrobial drugs [47]. A recent review shows that the thiazole scaffold is present in more than 18 FDA-approved drugs as well as in numerous experimental drugs [48]. The development of novel derivatives from this class could address antibiotic resistance at both free or planktonic cells (genetic resistance) and sessile bacterial communities called biofilms, which can be up to a thousand times more resistant than their planktonic counterparts to different antimicrobial agents (phenotypic resistance or tolerance) [49,50]. Benzothiazole compounds consisting of a benzene ring fused with the 4 and 5 positions of a thiazole ring have been found to exhibit a wide variety of biological activities, including antibacterial, tuberculostatic, antifungal, antiparasitic, antiviral and antioxidant effects [51,52,53,54,55,56]. Pyridine is a heterocyclic compound, which, either single or in association with other heterocycles, harbors many therapeutic activities, including antimicrobial, antiviral and antioxidant properties [57]. Pyrimidine-containing heterocyclic compounds are preferred leads for the development of novel antibacterial drugs, exhibiting the advantage of being found in many physiological molecules, allowing pyrimidine derivatives to interact with other biological molecules optimally [58].

Considering the literature cited in the present paper [2,6,29,30,31] and sustained by the conclusions traced after completing the molecular docking studies [34,45], the next step was the development of a synthesis strategy based on the advantage of merging two pharmacophores of thiourea and a heterocyclic ring (thiazole, benzothiazole, pyridine and pyrimidine) in a single molecular entity. 

The identity of the compounds was demonstrated in a series of qualitative analyses such as ^1^H NMR, ^13^C NMR, FTIR and FT-ICR.

The antimicrobial activity of compounds **1a**–**1g** was examined on standard bacterial strains *Staphylococcus aureus* ATCC 25923, *Enterococcus faecalis* ATCC 29212, *Escherichia coli* ATCC 25922 and *Pseudomonas aeruginosa* ATCC 27853. Ciprofloxacin (5 µg/mL) was used as a positive control. The tested compounds exhibited low antimicrobial activity against planktonic cells, with MIC values of 5000–1250 µg/mL compared to the antibiotic used as control (ciprofloxacin, MIC values of 0.012–0.62 µg/mL). These values are higher compared to other thiazole derivatives reported in the literature, the range of concentrations reported for different Gram-positive and Gram-negative bacterial strains, either reference or clinical, being from 2 to 400 µg/mL, while for *Candida* sp. between 25 and 100 µg/mL [59,60]. 

Moreover, the compounds were evaluated for their anti-biofilm activity, the MBIC values being established spectrophotometrically using the microtiter plate test and crystal violet assay. The recorded results were better than planktonic bacteria, with the MBIC values ranging between 5000 and 312 µg/mL. Candidates **1b** and **1d** were distinguished in the series through their good anti-biofilm activity against *E. coli* ATCC 25922 at MBIC values of 625 µg/mL.

For the evaluation of antioxidant activity [61,62,63,64,65,66,67], stock solutions of compounds **1a**–**1g** were made in the same solvent (acetone) with a standard concentration of 2 mg/mL. Sodium ascorbate was used as an antioxidant reference. The total antioxidant capacity (TAC) activity was investigated through spectrophotometric assay, where a mixture of equal parts of stock solution of DPPH and stock solution of each compound were kept in the dark for 30 min. The absorbance was measured at 517 nm through the well-known DPPH spectrophotometric assay. The highest antioxidant capacity has been recorded for compound **1d** (~43%), followed by **1f** (~25%). The antioxidant potential of thiourea derivatives has been highlighted in other studies [68]. One study of the mechanisms of the antioxidant ability of thiourea derivatives concluded that hydrogen atom transfer seems to be the preferred mechanism over single electron transfer when thiourea derivatives react with free radicals [69].

The viability of cells was assessed using fluorescein diacetate dye (FDA), a lipid-soluble, uncharged, and non-fluorescent dye, which can pass through intact membranes and be activated through hydrolysis to fluorescein upon uptake by nonspecific intracellular esterase. Accumulation of free fluorescein within intact cells leads to measurable fluorescence. However, damaged membranes allow the diffusion of fluorescein ions, resulting in non-fluorescent cells that are considered non-viable, assuming that damaged cells cannot recover due to the absence of membrane repair [70,71]. In addition, the compounds induced the G0/G1 cell-cycle arrest, accompanied by a proportional decrease in the percentage of cells in the S phase. The cell cycle plays a significant role in the regulatory mechanisms of cell growth, ensuring the maintenance of genomic integrity by protecting dividing cells from the potentially fatal consequences of DNA damage, such as apoptosis [71]. Other investigations are needed regarding the signaling pathways modulation, compounds metabolism, route of elimination and clearance.

Considering the results obtained after experimental evaluation of the compounds’ activities, we decided to develop and validate a reversed-phase high-performance liquid chromatography (RP-HPLC) method for the quantitative determination of compound **1d**, as it distinguishes itself in the tested series through its good anti-biofilm and antioxidant activities.

The validation of the analytical procedure has been performed in accordance with ICH Guideline Q2 (R1), and the typical validation characteristics were evaluated: specificity, detection limit, quantitation limit, linearity, range, precision (repeatability, intermediate precision) and accuracy. All relevant data collected during validation and formulae used for calculating validation characteristics are submitted in the present paper, and the chromatograms are attached in the Supplementary Materials section. 

The procedure was demonstrated to be suitable for its intended purpose. After evaluating the specificity of the method, no interference was observed between the analytical signal of the solvent and the peak of interest (compound **1d**). Peak purity angles recorded in the test solution chromatograms were lower than corresponding thresholds, indicating that the method is well suited for proper separation. The precision of the analytical method has been evaluated by repeatability (and intermediate precision), and RSD values did not exceed the acceptance threshold (2%). 

The detection and quantification limits were determined by analysis of samples with known concentrations of analyte and by establishing a minimum level at which the analyte could be reliably detected and quantified (0.0017 µg/mL for LOD and 0.053 µg/mL for LOQ). A linear relationship was determined across the range of 0.05 µg/mL to 40.00 µg/mL of the analytical procedure. The linearity was evaluated by inspection of a plot of signals as a function of analyte concentration. To demonstrate the linear relationship, the results were evaluated by appropriate statistical methods (correlation coefficient R^2^ ≥ 0.99). In addition, the correlation coefficient, y-intercept, and slope of the regression line have been submitted. The range is derived from a linearity study, 0.05 µg/mL–40.00 µg/mL of the analytical procedure, and provides an acceptable degree of linearity, accuracy and precision. The method’s accuracy was established over three concentration levels: 20 µg/mL–40.00 µg/mL **1d** solutions (three concentrations, six replicates each), covering the specific range, and the parameter was performed in duplicate in two different days (inter-day accuracy). The standard deviation, relative standard deviation and confidence interval for each determination have been submitted. 

The data collected, and the statistical data calculated based on chromatographic registrations demonstrated that the analytical method is suitable for the quantitative determination of compound **1d** and can be used for routine quality control analyses. 

## 4. Materials and Methods

### 4.1. Chemistry

The synthesis of the 2-((4-methoxyphenoxy)methyl)benzoic acid (Scheme 1) and 2-((4-methoxyphenoxy)methyl)benzoic acid chloride (**3**) were presented in a previous article [35,72].

#### 4.1.1. General Procedure for the Synthesis of the New Compounds (**1a**–**1g**)

A mixture of 2-((4-methoxyphenoxy)methyl)benzoyl chloride (MW 276.71 g/mol) (0.01 mol) and ammonium thiocyanate (MW 76.122 g/mol) (0.01 mol), in 20 mL of dried acetone was refluxed for 1 h in a round-bottom flask coupled with a condenser and a drying tube. Then, a solution of 0.01 mol of heterocyclic amine in 2 mL acetone was added drop-wise to the reaction mixture and refluxed for 2 h to obtain 2-((4-methoxy-phenoxy)methyl)benzoyl isothiocyanate (MW 299.34 g/mol) (Scheme 1). After cooling, the solution was poured into a beaker containing an ice–water mixture. The new acyl thiourea derivative precipitate was filtered, washed with water, dried and then recrystallized from isopropanol with active carbon. 

#### 4.1.2. Optimization Study for the Synthesis of **1b**

A suspension of ammonium thiocyanate (0.01 mol) in anhydrous acetone was placed in a round-bottomed flask fitted with an ascending condenser and attached tube of calcium chloride; the crude 2-(4-methoxyphenoxymethyl)benzoic acid chloride (0.01 mol) previously solubilized in 5 mL anhydrous acetone was added drop-wise, and also 0.1 mL solution of tetra n-butylammonium bromide (TBAB) 3% in anhydrous acetone.

The reaction mixture was refluxed for 30 min and then cooled to room temperature.

0.01 M of 2-aminobenzothiazole solubilized in acetone was added, and the mixture was heated for 45 min.

After cooling, the reaction mixture was poured into cold water. The precipitated crude thiouridine (**1b**) was filtered off, dried and recrystallized from isopropanol in the presence of charcoal.

### 4.2. Measurements

All reagents were used as received from Aldrich—Steinheim (Germany) and Merck—Darmstadt (Germany), except for ammonium thiocyanate, dried at 100 °C, and acetone, dried on calcium chloride and then distilled.

#### 4.2.1. TLC Analysis 

Monitoring the chemical reactions was carried out through thin-layer chromatography (TLC) [73] by using silica gel 60 GF245 (0.2 mm thick) precoated sheets (Merck, Germany), with chloroform/ethyl acetate (4:6) as a development solvent and visualization under ultraviolet light.

#### 4.2.2. Melting Points

Melting points were determined by Electrothermal 9100 apparatus (Bibby Scientific Ltd., Stone, UK), and the results were uncorrected.

#### 4.2.3. IR Spectra

The IR spectra were recorded on a Jasco FT/IR—4200 spectrometer (Jasco, Easton, MD, USA) using the ATR technique. The outcomes are presented as vs (very strong), s (strong), m (medium), or w (weak). 

#### 4.2.4. NMR Spectra

The ^1^H NMR and ^13^C NMR spectra were recorded in deuterated dimethyl sulfoxide (DMSO-d6) on a Bruker Fourier 300 MHz instrument (Bruker Corporation, Billerica, MA, USA), operating at 300 MHz for ^1^H NMR, and 75 MHz for ^13^C NMR.

In NMR spectra, the chemical shifts were recorded as δ values, in parts per million (ppm), relative to tetramethyl silane as internal standard, and coupling constants (J) in Hertz. The standard abbreviations that indicate the multiplicity of signals are s (singlet), d (doublet), m (multiplet), dd (double doublet), td (triple doublet), bs (broad singlet), and bd (broad doublet). The ^1^H NMR data are presented in the following order: chemical shifts, multiplicity, signal/atom attribution and the coupling constants. For ^13^C NMR data, the order is as follows: chemical shifts and signal/atom attribution. 

#### 4.2.5. FT-ICR Mass Spectrometry

The identity of the compounds was tested by the XR FTMS Hybrid System QqFTMS mass spectrometry with superconducting magnet SolariX XR 15T. The Fourier Transform ion cyclotron resonance spectrometer, SolariX XR 15T (Bruker Daltonics, Bremen, Germany), was used for the high-resolution mass spectrometry analysis. The sample solutions were analyzed through direct infusion and positive ESI ionization, selecting a flow rate of 120 μL/h. The investigation was carried out using N2 as nebulization gas, of 4 Barr, at 180 °C and a flow rate of 1.2 L/min. The spectra were recorded over a mass range between 92 and 1200 amu at a source voltage of 5500 V.

### 4.3. Biological Evaluation of the Antimicrobial Activity

Quantitative assessment of the antimicrobial activity of the tested compounds was performed using the standard broth microdilution method and the following test microorganisms: *Staphylococcus aureus* ATCC 25923, *Enterococcus faecalis* ATCC 29212, *Escherichia coli* ATCC 25922 and *Pseudomonas aeruginosa* ATCC 27853. Microbial suspensions were prepared for all the strains and adjusted to match the turbidity of the 0.5 McFarland standard. The suspensions were further diluted by 1/100. The tested compounds were solubilized in DMSO at a 10 mg/mL stock concentration. Binary dilutions of the compounds were prepared in Muller–Hinton broth in 96-well plates. Further, the different concentrations of the compounds were inoculated with the standard microbial suspensions. The microplates were incubated for 18–24 h at 37 °C under static conditions. Ciprofloxacin (5 µg/mL) was used as a positive control. Microbial growth control was represented by the broth inoculated with standard microbial suspensions. The minimum inhibitory concentrations (MIC) values were determined by subculturing the different broth dilutions on Muller–Hinton agar plates.

The anti-biofilm activity of the compounds was evaluated using the microtiter plate test and crystal violet assay. For this assay, the 96-well plates used to determine MIC values were emptied and washed three times with sterile saline to eliminate the planktonic bacterial cells. The biofilm-embedded cells from the plastic wells were fixed with 150 µL methanol for 5 min and then colored with 150 µL 1% violet crystal solution (prepared in distilled water) for 20 min. The excess staining solution was removed, and the microplates were washed with distillate water. The colored bacterial cells were re-suspended in 33% acetic acid solution, and the absorbance was measured at a wavelength of 490 nm. The minimum biofilm inhibitory concentration (MBIC), defined as the lowest concentration of compound required to inhibit the formation of biofilms, was determined by spectrophotometric assessment of the microplate wells as described above. The compounds’ anti-biofilm activity has also been confirmed by contrast-phase microscopy using an Olympus IX73 microscope. For this purpose, after the staining of the microplate with violet crystal, the wells were examined to observe the adherence to the wells’ material.

### 4.4. Total Antioxidant Activity 

Firstly, a fresh stock solution with the concentration of 2 × 10^−5^ M DPPH in acetone was made; similarly, stock solutions of compounds **1a**–**1g** were made in the same solvent (acetone was used due to high solubility of these compounds in this solvent, as well as for DPPH) with a standard concentration of 2 mg/mL. Sodium ascorbate was used as an antioxidant reference. For total antioxidant capacity (TAC) measurements, 1 mL of stock solution of DPPH and 1 mL of each compound was added. The mixture was kept in the dark (at room temperature) for 30 min, followed by the absorbance measuring at 517 nm (the λ_max_ value of DPPH solution), using a UVD-3500 UV-Vis spectrophotometer. The TAC percentage was calculated following Equation (1).

(1) $$\mathrm{TAC}\;\left(\%\right)=\frac{\left({\mathrm{Abs}}_{i}-{\mathrm{Abs}}_{30\;\min}\right)}{{\mathrm{Abs}}_{i}}\times 100$$

where Abs_i_ refers to the initial absorbance of the mixture, while Abs_30 min_ refers to the absorbance measured after 30 min [65,66,72]. 

### 4.5. Cytotoxicity and Cell Cycle Assay

Cells: In our experiments, we used ileocecal colorectal adenocarcinoma cells HCT-8 (ATCC CCL-244) and hepatocellular carcinoma cells HepG2 (ATCC HB-8065). For in vitro evaluation of cytotoxicity, N-acyl thiourea derivatives were solubilized in DMSO at a concentration of 10 mg/mL, then diluted to 1 mg/mL and filtered with 0.22 µm Millex-GV Filter (Merck, Darmstadt, Germany). 

Cytotoxicity: The viability of cells treated with the new N-acyl thiourea derivatives was examined using fluorescein diacetate staining (FDA). Briefly, 5 × 10^4^ cells seeded in 96-well plates were treated with binary dilutions of N-acyl thiourea derivatives beginning with 500 µg/mL. After 24 h, the morphological changes were observed under an inverted microscope Observer D (Carl Zeiss, Oberkochen, Germany). FDA dye was added, plates were placed in the IncuCyte^®^ S3 Live-Cell Analysis System (Sartorius AG, Göttingen, Germany), and five pictures per well were taken for 2 h. 

Cell-cycle analysis: DNA flow cytometry was used to evaluate each cell cycle phase distribution. HCT-8 were plated in 24-well plates at a concentration of 7.5 × 10^5^ cells/well and then treated with 100 µg/mL of the new N-acyl thiourea derivatives for 48 h. The cells were harvested, fixed in cold ethanol, stained with propidium iodide (PI), and analyzed on an EPICS XL flow cytometer (Becton Dickinson, San Jose, CA, USA). The FlowJo 7.2.5 software (Ashland, OR, USA: Becton, Dickinson and Company; 2021) was used to analyze the percentages of cells in each cell-cycle phase.

### 4.6. RP-HPLC Analysis

#### 4.6.1. Chromatographic Conditions

The Reversed-Phase High-Performance Liquid Chromatography (RP-HPLC) analysis [74] was employed to quantitatively determine a selected chemical compound (**1d**). 

The effective chromatographic separation was achieved on Inertsil ODS-3, 5 μm, 250 × 4.6 mm (stationary phase: octadecyl silica gel C18; length: 250 mm, internal diameter: 4.6 mm, particle size: 5 μm), with mobile phase mix of 6.8 g/L potassium dihydrogen phosphate R: acetonitrile R (80: 20%, V/V), by isocratic elution. 

The flow rate of the mobile phase was set at 1.0 mL/min; detection: λ = 275 nm; injection volume: 100 μL; run time: 20 min (around 4 times the retention time of the chromatographic peak due to compound 1d; typical retention time of the compound 1d: 5.6 min); column temperature: room temperature; sample temperature: room temperature. 

#### 4.6.2. Materials and Equipment

HPLC grade solvents were used in HPLC analysis: acetonitrile reagent (Fischer Scientific, Waltham, MA, USA), methanol reagent (Scharlau, Charm, Germany), and potassium dihydrogen phosphate reagent (Thermo Scientific, Waltham, MA, USA).

Waters Alliance HPLC system, comprised of the following modules: 2695 separation module, 996 PDA detector, PC equipped with “Empower 1 PDA Software,” was used for the quantitative determination. The samples were weighed using a Mettler Toledo analytical balance, and the pH of the solutions was determined with a Lab pH meter. Syringe nylon filters (0.45 µm) (Agilent Technologies) were used.

#### 4.6.3. Mobile Phase Preparation

The analysis was completed with an isocratic elution of 80% solvent A and 20% solvent B (*V*/*V*). To obtain solvent A, 6.8 g potassium dihydrogen phosphate R was transferred into a 1000 mL volumetric flask, dissolved in ultra-purified water, then the flask was brought to volume with the same solvent. Acetonitrile R, HPLC grade, represented solvent B.

#### 4.6.4. Samples Preparation

A reversed-phase high-performance liquid chromatography method for the quantitative determination of compound **1d** has been developed and validated in compliance with ICH guidelines [75].

For the validation of the quantitative analytical method, the following parameters were evaluated: specificity, precision (intermediate precision—repeatability), limit of detection (LOD), limit of quantitation (LOQ), linearity, range, and accuracy. 

##### Specificity

System suitability testing is a measure of evaluating the analytical performances of the equipment [76]. A blank solution represented by the solvent used at sample preparation (methanol) and three consecutive injections of 30 µg/mL compound **1d** has been registered for this reason. 

The system suitability was demonstrated by the evaluation of symmetry factor with values between 0.8–1.8 [77], theoretical plates, RSDs not more than 2% calculated for the areas, and retention time of the main peak in the chromatograms obtained with 30 µg/mL **1d** solution.

For the specificity parameter, a stock solution was prepared by dissolving the solid compound **1d** in acetonitrile R. A 30 µg/mL **1d** solution was obtained by diluting the stock solution in methanol R. The interfering study was determined concerning the solvent used, where interfering peaks would not appear in the chromatogram obtained with methanol R. The peak purity was also evaluated by determining the purity angle and threshold results. 

##### LOD/LOQ

The limit of detection (LOD) and limit of quantitation (LOQ) were carried out to determine the lowest quantity of an analyte that can be distinguished and quantitated from a substance within a stated confidence limit [78]. LOD and LOQ values were calculated based on the calibration curve at low concentrations. Increasingly concentrated solutions of compound **1d** were prepared, and the calibration curve was traced (0.05 µg/mL, 0.06 µg/mL, 0.08 µg/mL, 0.1 µg/mL and 0.2 µg/mL). The regression analysis was used to obtain the standard deviation, the slope of the calibration curve (y-intercept), and correlation coefficient (R^2^ ≥ 0.99). These elements were used to determine LOD and LOQ values, as expressed in Equation (2).

(2) $$\mathrm{LOD}/\;\mathrm{LOQ}=\frac{F\;\times \;\sigma \;}{b}$$

where F—3.3 in case of LOD/10.0 in case of LOQ; σ—standard deviation; b—slope of the calibration curve.

##### Linearity

The linearity of an analytical method demonstrates its ability to provide results that are directly proportional to the concentration of the analyte within a specific range of concentrations. The range of the concentrations chosen needs to include these concentrations for which the level of precision, accuracy, LOD/LOQ, and linearity have been demonstrated [79].

The linearity of the method was demonstrated within the concentration range of the theoretical working concentration (LOQ—40.0 µg/mL **1d** solution). In order to demonstrate the linearity of the method, a calibration curve was created by preparing increasingly concentrated solutions: 0.05 µg/mL, 0.06 µg/mL, 0.08 µg/mL, 0.1 µg/mL, 0.2 µg/mL, 20 µg/mL, 30 µg/mL and 40 µg/mL. The solutions were prepared in duplicate from 200 µg/mL **1d** solution. Then, 10.0 mg compound **1d** was dissolved and diluted to 50 mL with acetonitrile R (stock solution). The target concentrations were obtained by appropriate dilutions of stock solution, with methanol as dilution solvent. The analytical method proved linear over the range of LOQ—40.0 µg/mL **1d** solution, with a plot of concentration vs. peak area described by a correlation coefficient (R^2^) ≥ 0.99. 

##### Precision and Accuracy

The inter-day precision and accuracy studies were determined based on three concentration levels: 20 µg/mL **1d** solution, 30 µg/mL **1d** solution and 40 µg/mL **1d** solution. For every concentration, a stock solution was prepared from 10 mg, 15 mg and 20 mg compound **1d** dissolved in acetonitrile R. To reach the intended concentrations; suitable dilutions were made in methanol R. The solutions were prepared six times on two different days to demonstrate the repeatability of the method. The retention time and the peak areas must not exceed RSD (%) of 2%. 

To demonstrate the accuracy of the method, three concentration levels (low, medium and high concentration) were prepared and analyzed. For accuracy parameters, the obtained concentrations were evaluated concerning a solution of 30 µg/mL compound **1d**. Each sample solution’s recovery (%) fits in the 98–102% range. Moreover, % RSD values of recovery amount must not exceed 2%. 

## 5. Conclusions

In summary, various N-acyl thiourea derivatives with heterocyclic rings were synthesized to study their antimicrobial, anti-biofilm and antioxidant bioassay results. Among the new series of N-acyl thiourea derivatives with heterocyclic rings, the compound bearing the 6-methylpyridine moiety (**1d**) exhibits the most promising anti-biofilm and antioxidant activities, with potential applications in alleviating the toxicity against host cells and reducing the deleterious effects of exacerbated/chronic inflammatory reactions that often occur in infectious processes. The computational predictions for target binding affinities determined in molecular docking studies correlated with the compounds’ in vitro antimicrobial activity against selected microorganisms *Staphylococcus aureus* ATCC 25923, *Enterococcus faecalis* ATCC 29212, *Escherichia coli* ATCC 25922 and *Pseudomonas aeruginosa* ATCC 27853. Nevertheless, further studies are required to elucidate the mechanisms of the anti-biofilm and antioxidant activities and establish if the antioxidant feature could interfere with the microbial lethality induced by this compound. The cytotoxicity assay on mammalian cells revealed that the tested compounds induced a G0/G1 phase cell arrest, requiring further research to establish the molecular pathways involved. Another future issue is investigating the structure—biology activity relationships for the designed compounds. An RP-HPLC analytical method has been developed and validated for the quantitative determination of compound **1d**. By completing the typical validation characteristics, the results obtained have met the acceptance criteria. The method was demonstrated to be specific, precise, accurate and linear in a particular range of concentrations, confirming that it suits routine quality control analyses. 

## Figures and Tables

**Table 1:** The MIC values (µg/mL) obtained for the tested compounds.

Strain/ Compound	*Staphylococcus* *aureus* ATCC 25923	*Enterococcus faecalis* ATCC 29212	*Escherichia* *coli* ATCC 25922	*Pseudomonas aeruginosa* ATCC 27853
**1a**	1250	1250	1250	2500
**1b**	1250	1250	1250	2500
**1c**	1250	1250	1250	1250
**1d**	2500	1250	1250	2500
**1e**	2500	1250	1250	1250
**1f**	2500	1250	1250	2500
**1g**	2500	1250	1250	>5000
Ciprofloxacin	0.15	0.62	0.012	0.15
DMSO	12.5%	12.5%	12.5%	12.5%

**Table 2:** The MBIC values (µg/mL) obtained for the tested compounds.

Strain/ Compound	*Staphylococcus aureus* ATCC 25923	*Enterococcus* *faecalis* ATCC 29212	*Escherichia* *coli* ATCC 25922	*Pseudomonas* *aeruginosa* ATCC 27853
**1a**	2500	2500	2500	2500
**1b**	2500	1250	625	1250
**1c**	1250	1250	1250	1250
**1d**	1250	1250	625	1250
**1e**	>5000	>5000	5000	1250
**1f**	>5000	2500	1250	2500
**1g**	2500	1250	1250	1250
DMSO	25%	12.5%	6.25%	12.5%

**Table 3:** TAC values (%) for compounds **1a**–**1g**.

Compound	1a	1b	1c	1d	1e	1f	1g
TAC (%)	14.39	12.73	10.50	43.17	10.17	24.81	15.13

**Table 4:** LOD/LOQ results for compound **1d**, expressed in µg/mL, and % (considering 100%: 30 µg/mL **1d** solution).

STDEV	SLOPE	LOD		LOQ	
490.324	930,686.3	µg/mL	%	µg/mL	%
		0.0017	0.0057	0.053	0.0177

**Table 5:** Linearity results for compound **1d**, linearity performed over 0.05 µg/mL–40.00 µg/mL. Duplicate injections of each solution were registered in the chromatographic system.

Solution No.	Compound 1d Peak Area		
	Concentration (µg/mL)	Sample I	Sample II
1.	0.05	4072	3998
2.	0.06	4604	4710
3.	0.08	6406	6552
4.	0.10	7545	7346
5.	0.20	17,849	17,837
6.	20.00	117,640	116,577
7.	30.00	173,649	173,564
8.	40.00	3,274,981	3,276,413

**Table 6:** Intermediate precision results for 20 µg/mL **1d** solution.

Sample No.	Compound 1d Results—20 µg/mL Solution					
	Precision 1 Results ^(1)^		Precision 2 Results ^(2)^		Inter-Day Precision ^(3)^	
	Retention Time [min]	Compound 1d, Area of the Peak	Retention Time [min]	Compound 1d, Area of the Peak	Retention Time [min]	Area of the Peak
1.	5.668	1,683,470	5.587	1,691,833		
2.	5.668	1,671,584	5.606	1,690,608		
3.	5.668	1,692,685	5.602	1,736,221		
4.	5.668	1,676,085	5.598	1,697,748		
5.	5.668	1,674,530	5.582	1,704,577		
6.	5.668	1,675,112	5.601	1,697,552		
*Average*	*5.668*	*167,8911*	*5.596*	*1,703,090*	*5.632*	*1,691,000*
σ ^(4)^	0.00	7820.54	0.01	16,980.00	0.04	17,840.85
RSD ^(5)^, %	0.00	0.47	0.17	1.00	0.68	1.06

^(1)^ Precision results for compound **1d**—20 µg/mL solution compound **1d**—first day; ^(2)^ Precision results for compound **1d**—20 µg/mL solution compound **1d**—second day; ^(3)^ Inter-day precision results for compound **1d** (Precision 1 + Precision 2)—20 µg/mL solution compound **1d**; ^(4)^ σ—Standard Deviation; ^(5)^ RSD—Relative Standard Deviation.

**Table 7:** Intermediate precision results for 30 µg/mL **1d** solution.

Sample No.	Compound 1d Results—30 µg/mL Solution					
	Precision 1 Results ^(1)^		Precision 2 Results ^(2)^		Inter-Day Precision ^(3)^	
	Retention Time [min]	Compound 1d, Area of the Peak	Retention Time [min]	Compound 1d, Area of the Peak	Retention Time [min]	Area of the Peak
1.	5.670	2,518,986	5.596	2,614,065		
2.	5.668	2,510,959	5.600	2,594,510		
3.	5.664	2,537,030	5.611	2,607,145		
4.	5.665	2,510,454	5.610	2,564,753		
5.	5.665	2,518,396	5.605	2,603,224		
6.	5.664	2,514,887	5.717	2,617,823		
*Average*	*5.666*	*2,518,452*	*5.623*	*2,600,253*	*5.645*	*2,559,353*
σ ^(4)^	0.00	9780.11	0.05	19,230.37	0.04	45,127.76
RSD ^(5)^, %	0.04	0.39	0.82	0.74	0.68	1.76

^(1)^ Precision results for compound **1d**—30 µg/mL solution compound 1d—first day; ^(2)^ Precision results for compound **1d**—30 µg/mL solution compound **1d**—second day; ^(3)^ Inter-day precision results for compound **1d** (Precision 1 + Precision 2)—30 µg/mL solution compound **1d**; ^(4)^ σ—Standard Deviation; ^(5)^ RSD—Relative Standard Deviation.

**Table 8:** Intermediate precision results for 40 µg/mL **1d** solution.

Sample No.	Compound 1d Results—40 µg/mL Solution					
	Precision 1 Results ^(1)^		Precision 2 Results ^(2)^		Inter-Day Precision ^(3)^	
	Retention Time [min]	Compound 1d, Area of the Peak	Retention Time [min]	Compound 1d, Area of the Peak	Retention Time [min]	Area of the Peak
1.	5.639	3,274,981	5.602	3,259,885		
2.	5.629	3,276,413	5.594	3,293,681		
3.	5.636	3,288,268	5.590	3,297,946		
4.	5.628	3,281,385	5.603	3,291,424		
5.	5.620	3,287,538	5.591	3,297,122		
6.	5.622	3,288,522	5.598	3,328,312		
*Average*	*5.629*	*3,282,851*	*5.596*	*3,294,728*	*5.613*	*3,288,790*
σ ^(4)^	0.01	6148.27	0.01	21,771.63	0.02	16,465.46
RSD ^(5)^, %	0.13	0.19	0.10	0.66	0.32	0.50

^(1)^ Precision results for compound **1d**—40 µg/mL solution compound **1d**—first day; ^(2)^ Precision results for compound **1d**—40 µg/mL solution compound **1d**—second day; ^(3)^ Inter-day precision results for compound **1d** (Precision 1 + Precision 2)—40 µg/mL solution compound **1d**; ^(4)^ σ—Standard Deviation; ^(5)^ RSD—Relative Standard Deviation.

**Table 9:** Compound **1d** mean percent recovery—Intra-day accuracy results (first day).

Sample No.	Compound 1d Concentration								
	20 µg/mL Solution			30 µg/mL Solution			40 µg/mL Solution		
	Real (µg/mL)	Determined (µg/mL)	Recovery (%)	Real (µg/mL)	Determined (µg/mL)	Recovery %	Real (µg/mL)	Determined (µg/mL)	Recovery (%)
1.	20.0800	20.2859	101.03	30.2000	30.3540	100.51	39.8000	39.4638	99.16
2.	20.0800	20.1427	100.31	30.2000	30.2572	100.19	39.8000	39.4810	99.20
3.	20.0800	20.3970	101.58	30.2000	30.5714	101.23	39.8000	39.6239	99.56
4.	20.0800	20.1970	100.58	30.2000	30.2512	100.17	39.8000	39.5409	99.35
5.	20.0800	20.1782	100.49	30.2000	30.3469	100.49	39.8000	39.6151	99.54
6.	20.0800	20.1852	100.52	30.2000	30.3046	100.35	39.8000	39.6269	99.57
*Average:*			*100.21*						
Standard Deviation:			0.70						
Relative standard deviation (RSD%):			0.70						
Confidence interval (Probability 95%):			99.89–100.53						

**Table 10:** Compound **1d** mean percent recovery—Intra-day accuracy results (second day).

Sample No.	Compound 1d Concentration								
	20 µg/mL Solution			30 µg/mL Solution			40 µg/mL Solution		
	Real (µg/mL)	Determined (µg/mL)	Recovery (%)	Real (µg/mL)	Determined (µg/mL)	Recovery %	Real (µg/mL)	Determined (µg/mL)	Recovery (%)
1.	20.5000	20.3394	99.22	31.0000	31.4265	101.3758	39.9000	39.1906	98.2220
2.	20.5000	20.3246	99.14	31.0000	31.1914	100.6174	39.9000	39.5969	99.2403
3.	20.5000	20.8730	101.82	31.0000	31.3433	101.1074	39.9000	39.6482	99.3688
4.	20.5000	20.4105	99.56	31.0000	30.8337	99.4634	39.9000	39.5698	99.1723
5.	20.5000	20.4926	99.96	31.0000	31.2962	100.9554	39.9000	39.6383	99.3440
6.	20.5000	20.4081	99.55	31.0000	31.4717	101.5215	39.9000	40.0132	100.2838
*Average:*			*100.00*						
Standard Deviation:			1.01						
Relative standard deviation (RSD%):			1.01						
Confidence interval (Probability 95%):			99.53–100.47						

## Data Availability

Not applicable.

## Supplementary Materials

The following supporting information can be downloaded at: https://www.mdpi.com/article/10.3390/antibiotics12050807/s1, Chromatograms supporting the RP-HPLC validation of the analytical method.

## Sample Availability

Samples of the compounds are available from the authors.
