# Supplementary material for: New N-acyl Thiourea Derivatives: Synthesis, Standardized Quantification Method and In Vitro Evaluation of Potential Biological Activities
Source: Antibiotics (Basel). 2023 Apr 25;12(5):807. doi: 10.3390/antibiotics12050807 (PMC10215470; doi:10.3390/antibiotics12050807)
Supplement: Supplementary file 1 [file antibiotics-12-00807-s001.zip › antibiotics-2313405-supplementary.pdf]

Quantitative determination of
compound 1d
# - Validation of the analytical method -

Validation parameters:

- Specificity
- Precision 1
- Precision 2
- LOD – LOQ
- Linearity
- Range
- Accuracy

Quantitative determination of

compound 1d

- Validation of the analytical method -

Validation parameters:

- **Specificity**
- Precision 1
- Precision 2
- LOD – LOQ
- Linearity
- Range
- Accuracy

Specificity_1d_Report

Reported by User: Roxana Roman (Roxana_Roman)
Acquisition Server Waters7
Project Name: Test
Sample Set Name: Precision 1_Accuracy_PyridineM
Code column: Inertsil ODS-3, 4,6*250 mm, 5 um

SAMPLE INFORMATION

Sample Name:	Methyl-pyridine 30 ug/ mL		
Sample Type:	Standard	Date Acquired:	11.06.2022 18:49:07
Vial:	25	Acq. Method Set:	Precision 1_Accuracy_PyridineM
Injection #:	1	Date Processed:	16.02.2023 13:11:06
Injection Volume:	100,00 ul	Processing Method:	Precision 1_Accuracy_PyridineM
Run Time:	20,0 Minutes	Channel Name:	Extract 275,0
Acquired By:	Roxana_Roman	Proc. Chnl. Descr.:	PDA 275,0 nm, Smoothed by 25

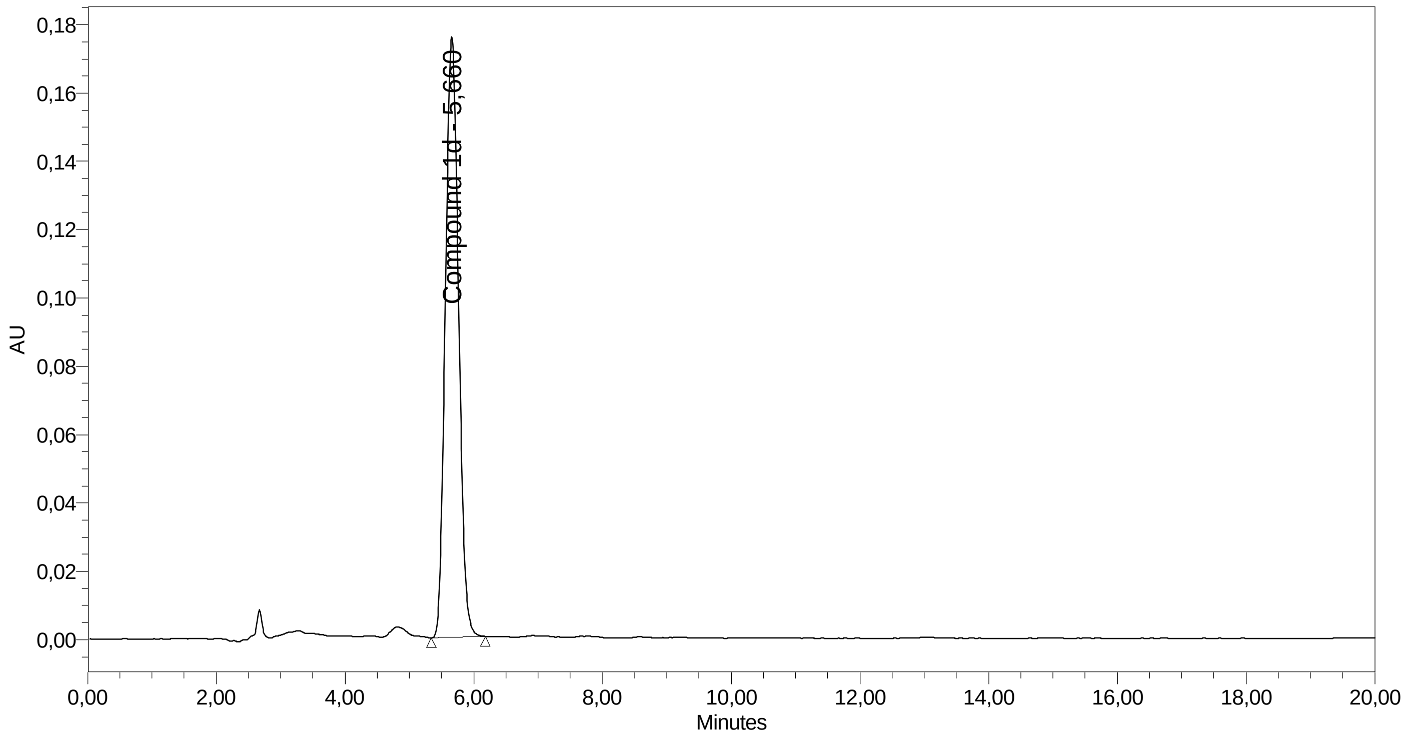

	Peak Name	RT	Area	Height (µV)	Symmetry Factor	Purity1 Angle	Purity1 Threshold	Purity1 Flag
1	Compound 1d	5,660	2504606	175677	1,09	0,082	0,279	No

	USP Plate Count
1	3596

Specificity_1d_Report

Reported by User: Roxana Roman (Roxana_Roman)
Acquisition Server Waters7
Project Name: Test
Sample Set Name: Precision 1_Accuracy_PyridineM
Code column: Inertsil ODS-3, 4,6*250 mm, 5 um

SAMPLE INFORMATION

Sample Name:	Methyl-pyridine 30 ug/ mL		
Sample Type:	Standard	Date Acquired:	11.06.2022 19:09:51
Vial:	25	Acq. Method Set:	Precision 1_Accuracy_PyridineM
Injection #:	2	Date Processed:	16.02.2023 13:11:08
Injection Volume:	100,00 ul	Processing Method:	Precision 1_Accuracy_PyridineM
Run Time:	20,0 Minutes	Channel Name:	Extract 275,0
Acquired By:	Roxana_Roman	Proc. Chnl. Descr.:	PDA 275,0 nm, Smoothed by 25

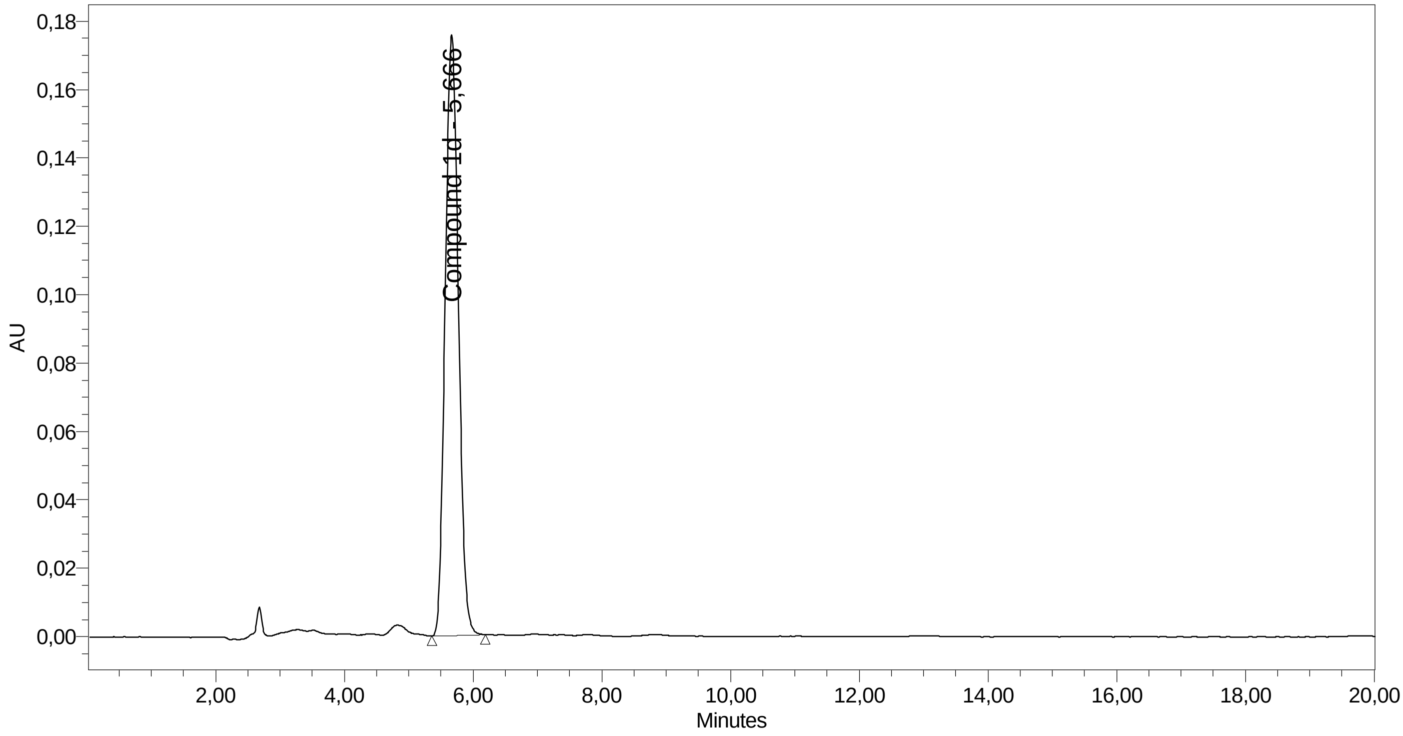

	Peak Name	RT	Area	Height (µV)	Symmetry Factor	Purity1 Angle	Purity1 Threshold	Purity1 Flag
1	Compound 1d	5,666	2511937	175638	1,09	0,077	0,278	No

	USP Plate Count
1	3573

Specificity_1d_Report

Reported by User: Roxana Roman (Roxana_Roman)
Acquisition Server Waters7
Project Name: Test
Sample Set Name: Precision 1_Accuracy_PyridineM
Code column: Inertsil ODS-3, 4,6*250 mm, 5 um

SAMPLE INFORMATION

Sample Name:	Methyl-pyridine 30 ug/ mL		
Sample Type:	Standard	Date Acquired:	11.06.2022 19:30:35
Vial:	25	Acq. Method Set:	Precision 1_Accuracy_PyridineM
Injection #:	3	Date Processed:	16.02.2023 13:11:09
Injection Volume:	100,00 ul	Processing Method:	Precision 1_Accuracy_PyridineM
Run Time:	20,0 Minutes	Channel Name:	Extract 275,0
Acquired By:	Roxana_Roman	Proc. Chnl. Descr.:	PDA 275,0 nm, Smoothed by 25

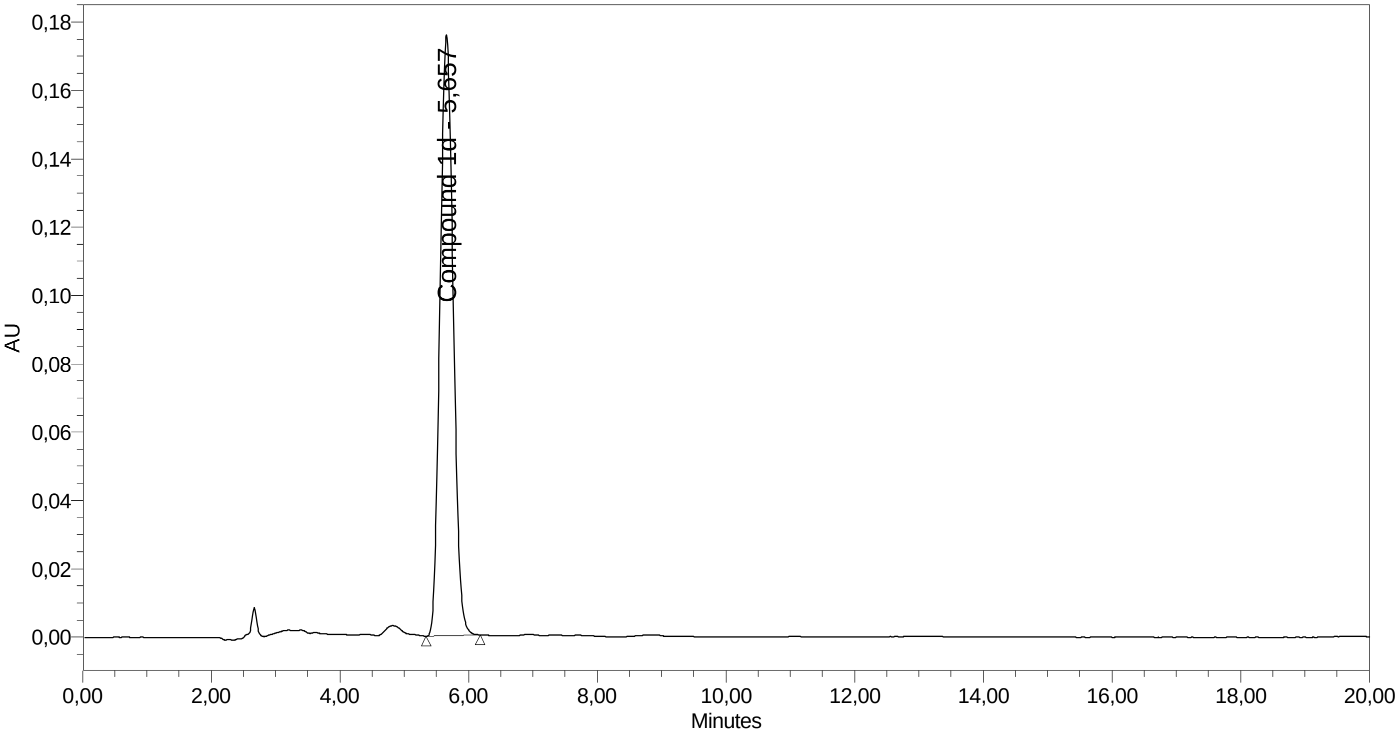

	Peak Name	RT	Area	Height (µV)	Symmetry Factor	Purity1 Angle	Purity1 Threshold	Purity1 Flag
1	Compound 1d	5,657	2512040	175781	1,09	0,085	0,280	No

	USP Plate Count
1	3559

Specificity_1d_Report

Reported by User: Roxana Roman (Roxana_Roman)
Acquisition Server Waters7
Project Name: Test
Sample Set Name: Precision 1_Accuracy_PyridineM
Code column: Inertsil ODS-3, 4,6*250 mm, 5 um

SAMPLE INFORMATION

Sample Name:	Methanol		
Sample Type:	Standard	Date Acquired:	11.06.2022 18:28:06
Vial:	11	Acq. Method Set:	Precision 1_Accuracy_PyridineM
Injection #:	1	Date Processed:	18.02.2023 09:25:05
Injection Volume:	100,00 ul	Processing Method:	Precision 1_Accuracy_PyridineM
Run Time:	20,0 Minutes	Channel Name:	Extract 275,0
Acquired By:	Roxana_Roman	Proc. Chnl. Descr.:	PDA 275,0 nm, Smoothed by 25

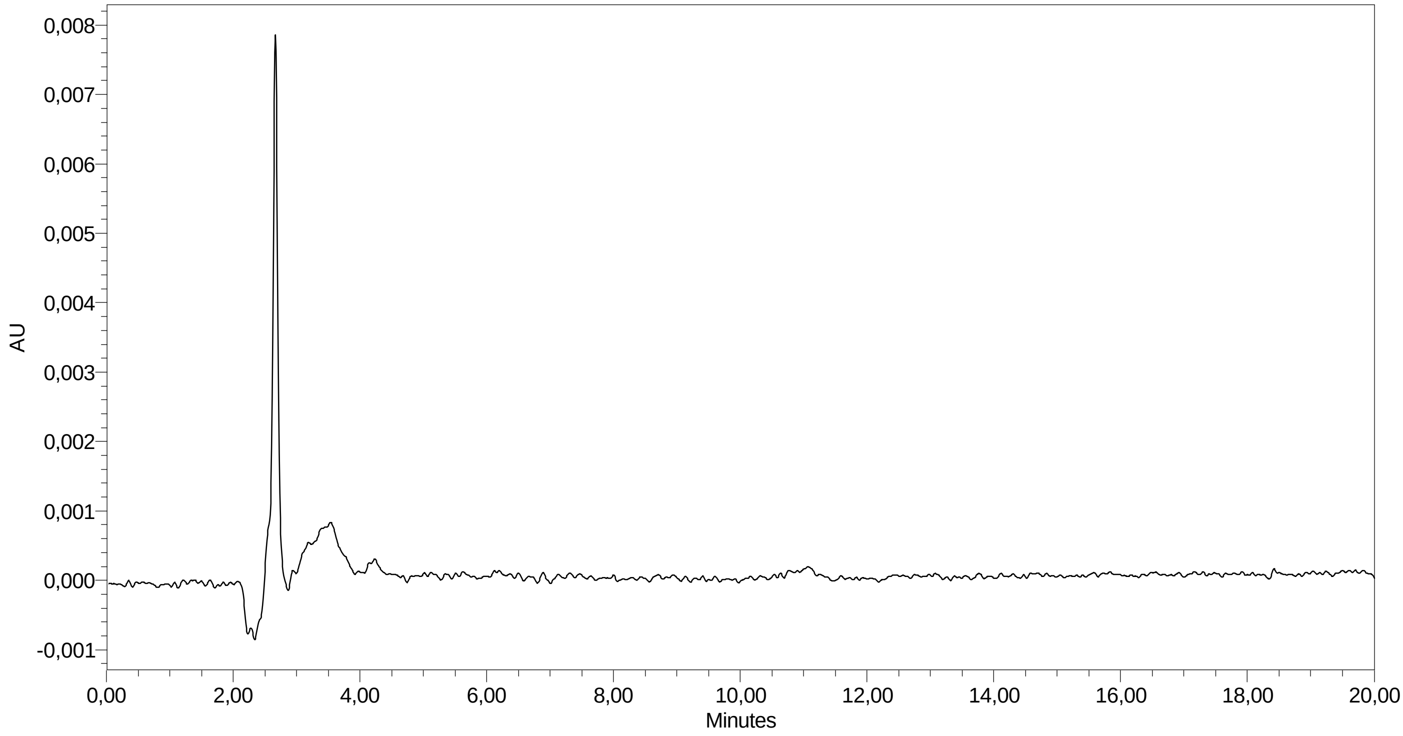

Basic LC Peaks Table group contains no data.

Specificity_1d_Report

Reported by User: Roxana Roman (Roxana_Roman)
Acquisition Server Waters7
Project Name: Test
Sample Set Name: Precision 1_Accuracy_PyridineM
Code column: Inertsil ODS-3, 4,6*250 mm, 5 um

SAMPLE INFORMATION

Sample Name:	Methyl-pyridine 30 ug/ mL		
Sample Type:	Unknown	Date Acquired:	11.06.2022 22:21:21
Vial:	32	Acq. Method Set:	Precision 1_Accuracy_PyridineM
Injection #:	1	Date Processed:	16.02.2023 13:12:52
Injection Volume:	100,00 ul	Processing Method:	Precision 1_Accuracy_PyridineM
Run Time:	20,0 Minutes	Channel Name:	Extract 275,0
Acquired By:	Roxana_Roman	Proc. Chnl. Descr.:	PDA 275,0 nm, Smoothed by 25

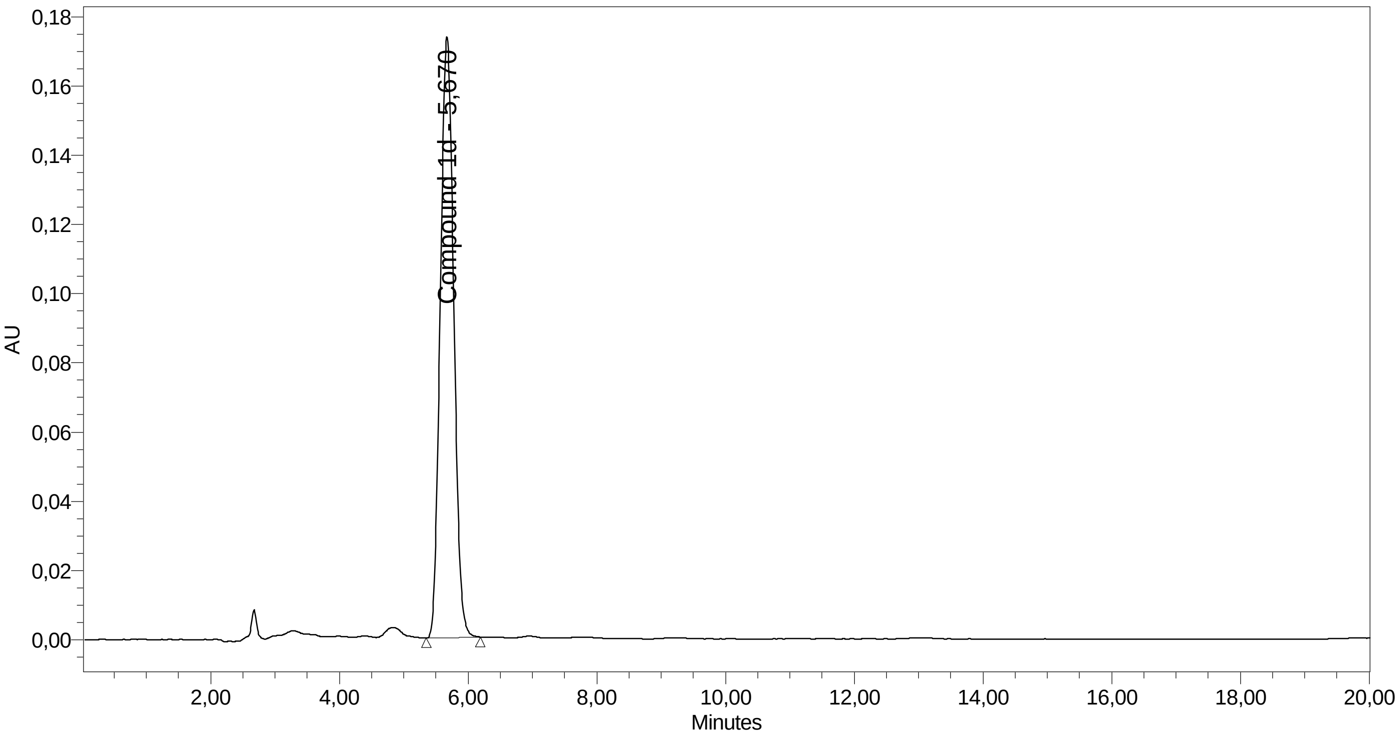

	Peak Name	RT	Area	Height (µV)	Symmetry Factor	Purity1 Angle	Purity1 Threshold	Purity1 Flag
1	Compound 1d	5,670	2518986	173649	1,07	0,081	0,279	No

	USP Plate Count
1	3463

Specificity_1d_Report

Reported by User: Roxana Roman (Roxana_Roman)
Acquisition Server Waters7
Project Name: Test
Sample Set Name: Precision 1_Accuracy_PyridineM
Code column: Inertsil ODS-3, 4,6*250 mm, 5 um

S A M P L E   I N F O R M A T I O N

Sample Name:	Methyl-pyridine 30 ug/ mL		
Sample Type:	Unknown	Date Acquired:	11.06.2022 22:42:09
Vial:	33	Acq. Method Set:	Precision 1_Accuracy_PyridineM
Injection #:	1	Date Processed:	16.02.2023 13:13:03
Injection Volume:	100,00 ul	Processing Method:	Precision 1_Accuracy_PyridineM
Run Time:	20,0 Minutes	Channel Name:	Extract 275,0
Acquired By:	Roxana_Roman	Proc. Chnl. Descr.:	PDA 275,0 nm, Smoothed by 25

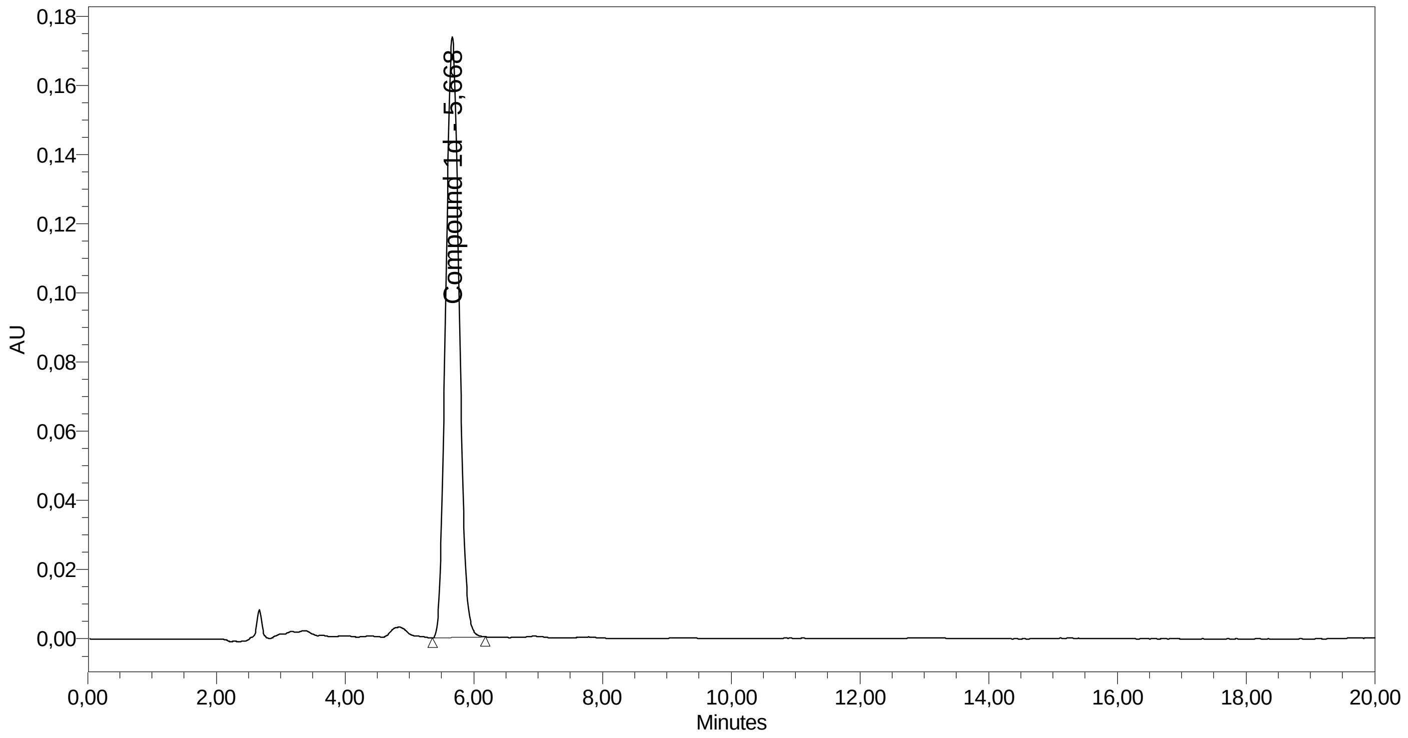

	Peak Name	RT	Area	Height (µV)	Symmetry Factor	Purity1 Angle	Purity1 Threshold	Purity1 Flag
1	Compound 1d	5,668	2510959	173564	1,08	0,080	0,280	No

	USP Plate Count
1	3476

Quantitative determination of

compound 1d

- Validation of the analytical method -

Validation parameters:

- Specificity
- Precision 1
- Precision 2
- **LOD – LOQ**
- Linearity
- Range
- Accuracy

Seria Metoxi_1d_LOD LOQ

Reported by User: Roxana Roman (Roxana_Roman)
Acquisition Server Waters7
Project Name: Test
Sample Set Name: LOD_LOQ_Linearity_Pyridine Met
Code column: Inertsil ODS-3, 4,6*250 mm, 5 um

Calibration Plot group for Solvent peak contains no data.

Calibration Plot

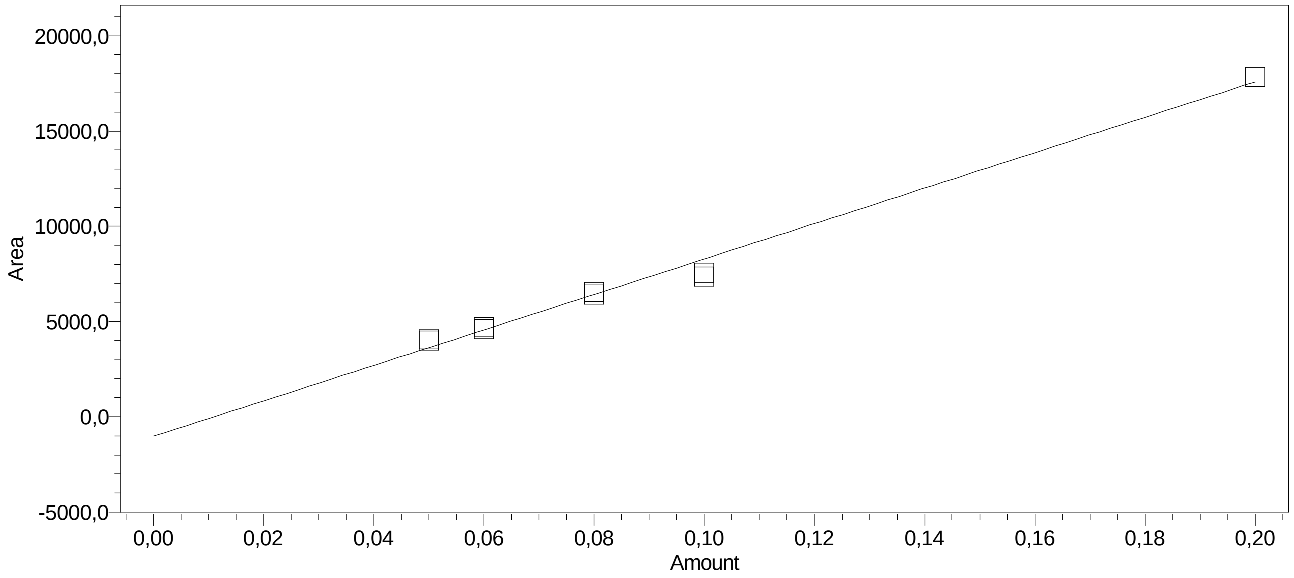

Name: Compound 1d; Processing Method: Linearity_Pyridine_M_Methoxy; Fit Type: Linear (1st Order); Cal Curve Id: 4621; A: -1,028826e+003; B: 9,306863e+004; C: 0,000000e+000; D: 0,000000e+000; R^2: 0,992391; Standard Error 4,903240e+002

Point Information ' Peak: Solvent peak' table contains no data.

Peak: Compound 1d

	Name	Level	X Value	Response	Calc. Value	% Deviation	Manual	Ignore
1	Compound 1d	5	0,050000	4071,743656	0,054804	9,609	No	No
2	Compound 1d	5	0,050000	3997,911266	0,054011	8,022	No	No
3	Compound 1d	6	0,060000	4710,043738	0,061663	2,771	No	No
4	Compound 1d	6	0,060000	4604,067650	0,060524	0,873	No	No
5	Compound 1d	8	0,080000	6552,166667	0,081456	1,820	No	No
6	Compound 1d	8	0,080000	6405,660466	0,079882	-0,148	No	No
7	Compound 1d	10	0,100000	7346,426958	0,089990	-10,010	No	No
8	Compound 1d	10	0,100000	7544,638514	0,092120	-7,880	No	No
9	Compound 1d	20	0,200000	17837,293510	0,202712	1,356	No	No
10	Compound 1d	20	0,200000	17849,038191	0,202838	1,419	No	No

LOD LOQ_1d_Report

Reported by User: Roxana Roman (Roxana_Roman)
Acquisition Server Waters7
Project Name: Test
Sample Set Name: LOD_LOQ_Linearity_Pyridine Met
Code column: Inertsil ODS-3, 4,6*250 mm, 5 um

SAMPLE INFORMATION

Sample Name:	Solution 0.05 ug/ mL		
Sample Type:	Standard	Date Acquired:	11.06.2022 12:45:55
Vial:	3	Acq. Method Set:	LOD_LOQ_Linearity_Pyridine_Met
Injection #:	1	Date Processed:	11.02.2023 13:10:22
Injection Volume:	100,00 ul	Processing Method:	Linearity_Pyridine_M_Methoxy
Run Time:	20,0 Minutes	Channel Name:	Extract 275,0
Acquired By:	Roxana_Roman	Proc. Chnl. Descr.:	PDA 275,0 nm, Smoothed by 25

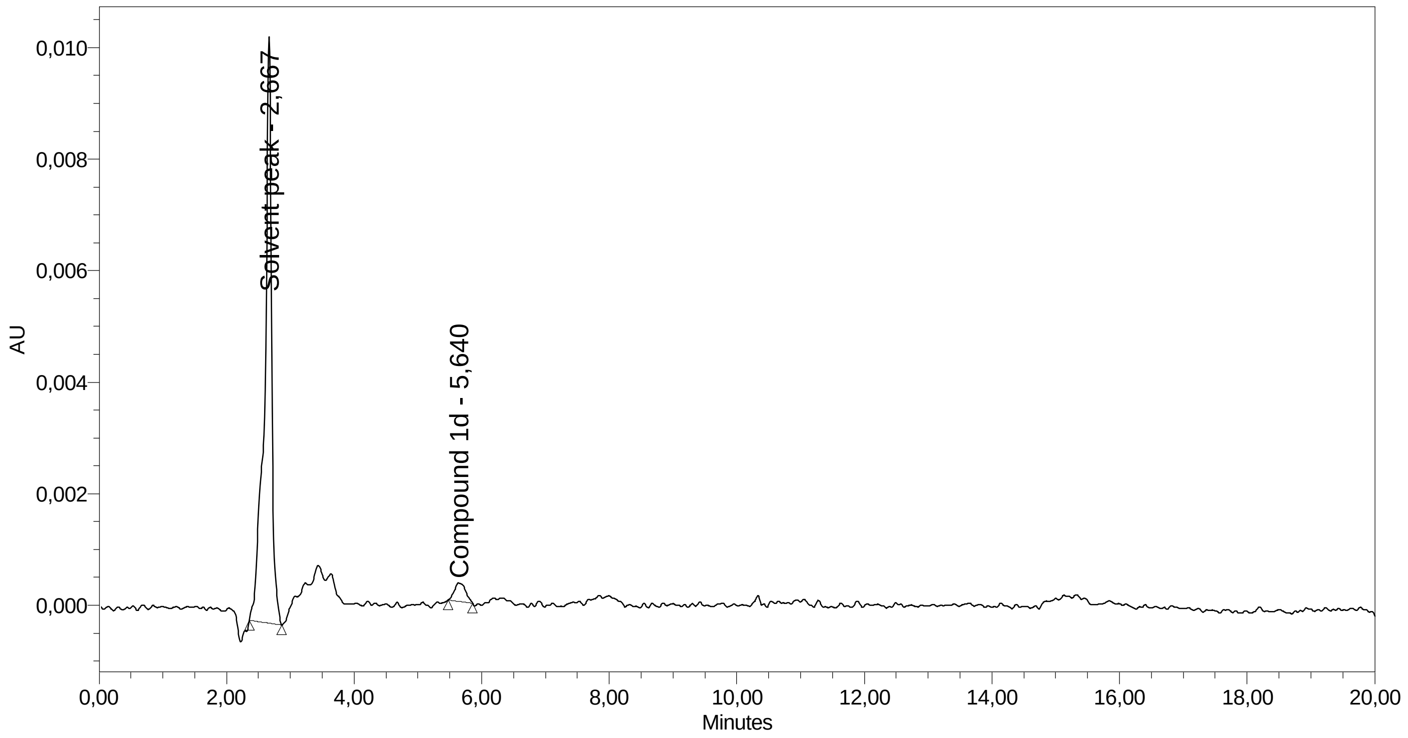

	Peak Name	RT	Area	Height (µV)	Level
1	Solvent peak	2,667	81106	10502	5
2	Compound 1d	5,640	4072	328	5

LOD LOQ_1d_Report

Reported by User: Roxana Roman (Roxana_Roman)
Acquisition Server Waters7
Project Name: Test
Sample Set Name: LOD_LOQ_Linearity_Pyridine Met
Code column: Inertsil ODS-3, 4,6*250 mm, 5 um

S A M P L E I N F O R M A T I O N

Sample Name:	Solution 0.05 ug/ mL		
Sample Type:	Standard	Date Acquired:	11.06.2022 13:06:54
Vial:	3	Acq. Method Set:	LOD_LOQ_Linearity_Pyridine_Met
Injection #:	2	Date Processed:	11.02.2023 13:10:39
Injection Volume:	100,00 ul	Processing Method:	Linearity_Pyridine_M_Methoxy
Run Time:	20,0 Minutes	Channel Name:	Extract 275,0
Acquired By:	Roxana_Roman	Proc. Chnl. Descr.:	PDA 275,0 nm, Smoothed by 25

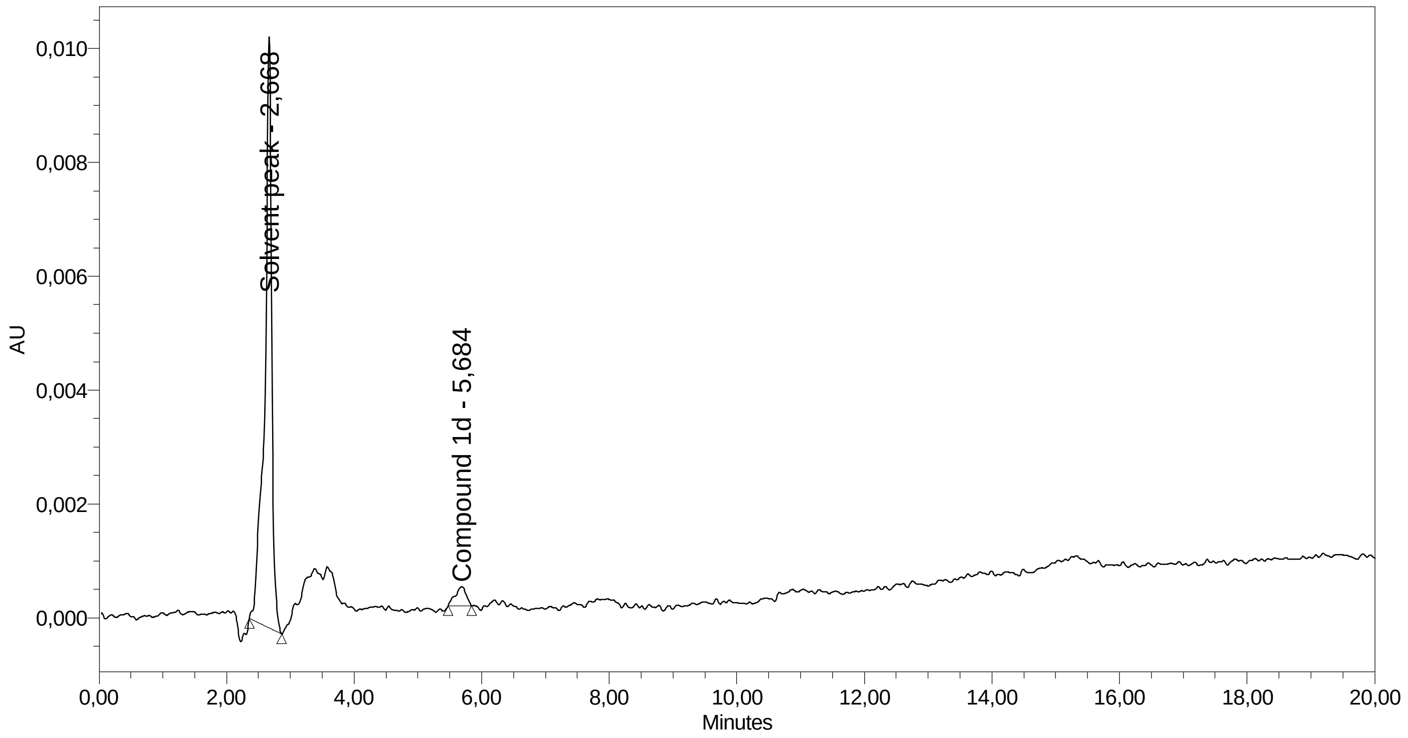

	Peak Name	RT	Area	Height (µV)	Level
1	Solvent peak	2,668	79221	10379	5
2	Compound 1d	5,684	3998	337	5

LOD LOQ_1d_Report

Reported by User: Roxana Roman (Roxana_Roman)
Acquisition Server Waters7
Project Name: Test
Sample Set Name: LOD_LOQ_Linearity_Pyridine Met
Code column: Inertsil ODS-3, 4,6*250 mm, 5 um

SAMPLE INFORMATION

Sample Name:	Solution 0.06 ug/ mL		
Sample Type:	Standard	Date Acquired:	11.06.2022 12:03:47
Vial:	4	Acq. Method Set:	LOD_LOQ_Linearity_Pyridine_Met
Injection #:	1	Date Processed:	11.02.2023 13:09:51
Injection Volume:	100,00 ul	Processing Method:	Linearity_Pyridine_M_Methoxy
Run Time:	20,0 Minutes	Channel Name:	Extract 275,0
Acquired By:	Roxana_Roman	Proc. Chnl. Descr.:	PDA 275,0 nm, Smoothed by 25

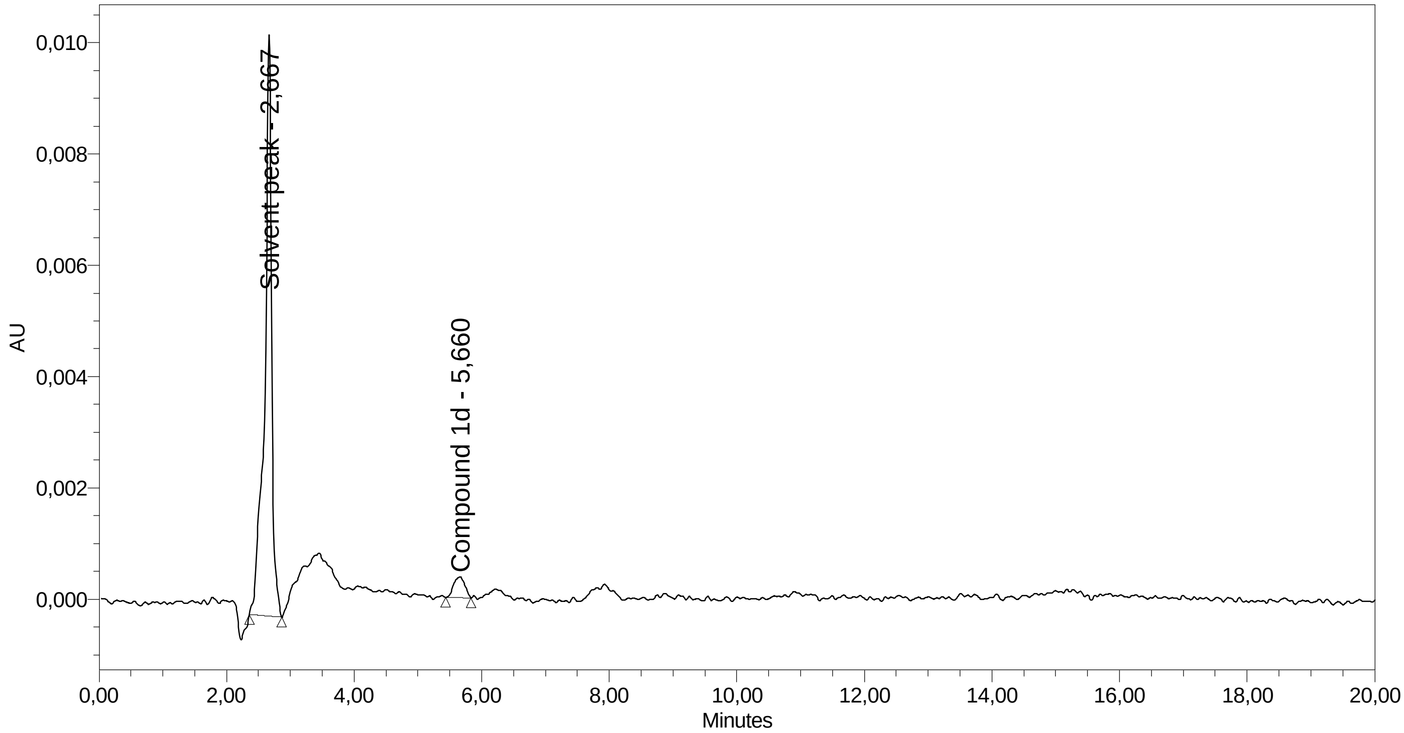

	Peak Name	RT	Area	Height (µV)	Level
1	Solvent peak	2,667	79126	10433	6
2	Compound 1d	5,660	4604	378	6

LOD LOQ_1d_Report

Reported by User: Roxana Roman (Roxana_Roman)
Acquisition Server Waters7
Project Name: Test
Sample Set Name: LOD_LOQ_Linearity_Pyridine Met
Code column: Inertsil ODS-3, 4,6*250 mm, 5 um

SAMPLE INFORMATION

Sample Name:	Solution 0.06 ug/ mL		
Sample Type:	Standard	Date Acquired:	11.06.2022 12:24:32
Vial:	4	Acq. Method Set:	LOD_LOQ_Linearity_Pyridine_Met
Injection #:	2	Date Processed:	11.02.2023 13:09:39
Injection Volume:	100,00 ul	Processing Method:	Linearity_Pyridine_M_Methoxy
Run Time:	20,0 Minutes	Channel Name:	Extract 275,0
Acquired By:	Roxana_Roman	Proc. Chnl. Descr.:	PDA 275,0 nm, Smoothed by 25

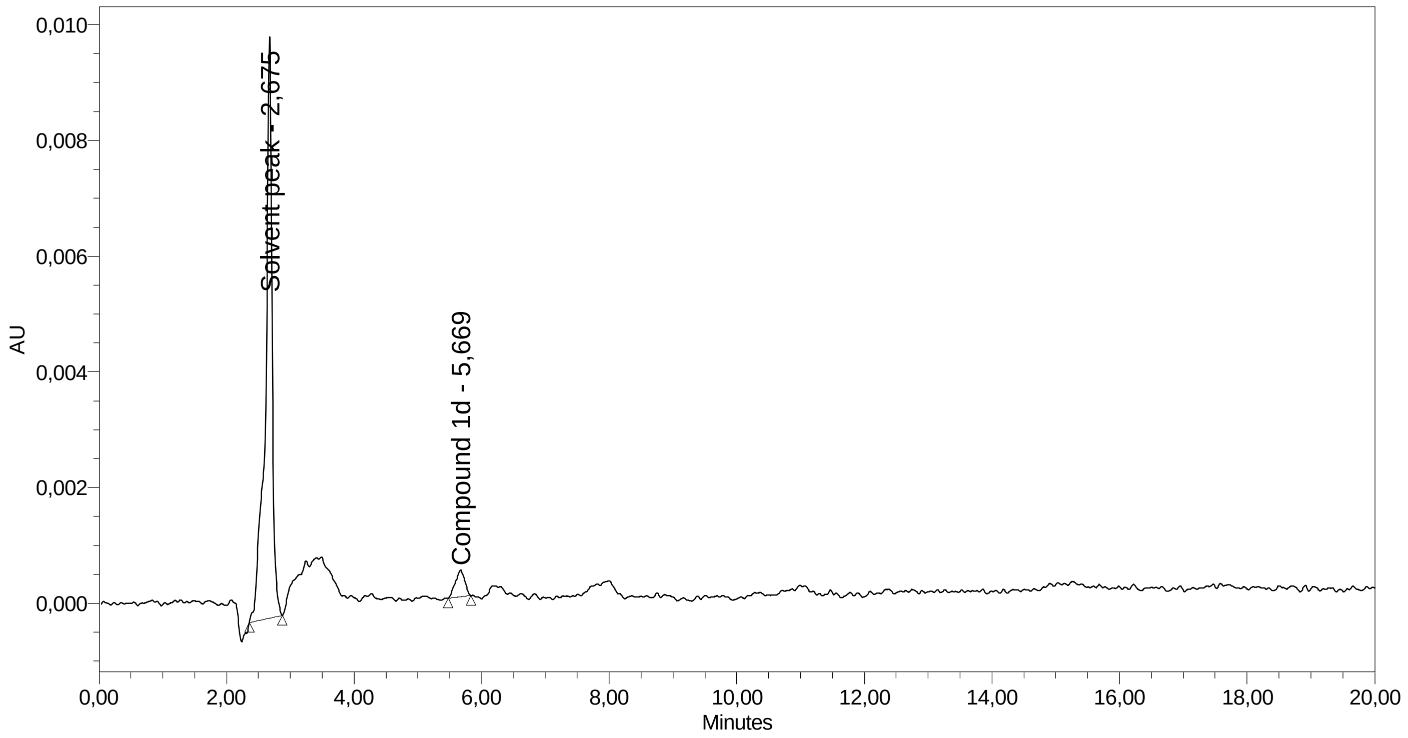

	Peak Name	RT	Area	Height (µV)	Level
1	Solvent peak	2,675	73641	10037	6
2	Compound 1d	5,669	4710	462	6

LOD LOQ_1d_Report

Reported by User: Roxana Roman (Roxana_Roman)
Acquisition Server Waters7
Project Name: Test
Sample Set Name: LOD_LOQ_Linearity_Pyridine Met
Code column: Inertsil ODS-3, 4,6*250 mm, 5 um

S A M P L E   I N F O R M A T I O N

Sample Name:	Solution 0.08 ug/ mL		
Sample Type:	Standard	Date Acquired:	11.06.2022 13:27:42
Vial:	5	Acq. Method Set:	LOD_LOQ_Linearity_Pyridine_Met
Injection #:	1	Date Processed:	11.02.2023 13:09:17
Injection Volume:	100,00 ul	Processing Method:	Linearity_Pyridine_M_Methoxy
Run Time:	20,0 Minutes	Channel Name:	Extract 275,0
Acquired By:	Roxana_Roman	Proc. Chnl. Descr.:	PDA 275,0 nm, Smoothed by 25

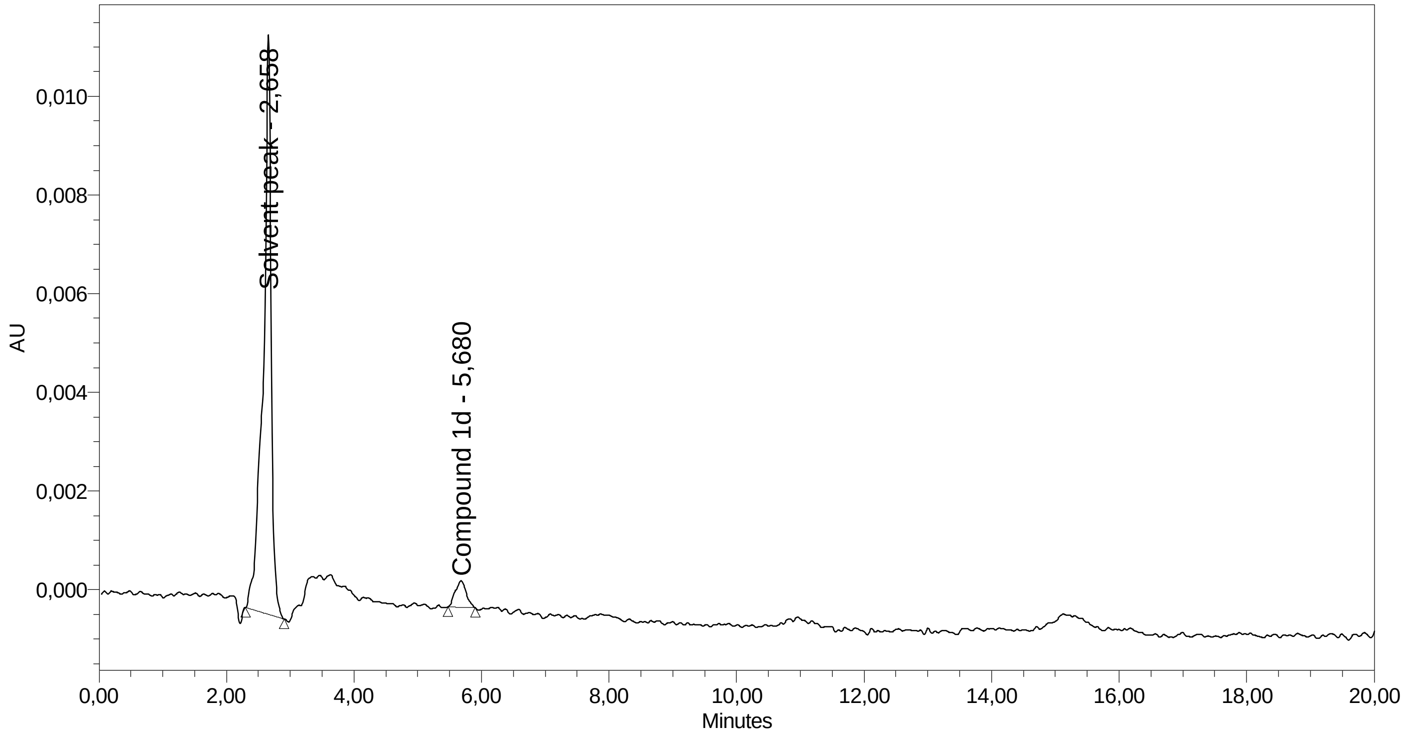

	Peak Name	RT	Area	Height (µV)	Level
1	Solvent peak	2,658	101752	11732	8
2	Compound 1d	5,680	6406	539	8

LOD LOQ_1d_Report

Reported by User: Roxana Roman (Roxana_Roman)
Acquisition Server Waters7
Project Name: Test
Sample Set Name: LOD_LOQ_Linearity_Pyridine Met
Code column: Inertsil ODS-3, 4,6*250 mm, 5 um

SAMPLE INFORMATION

Sample Name:	Solution 0.08 ug/ mL		
Sample Type:	Standard	Date Acquired:	11.06.2022 13:48:26
Vial:	5	Acq. Method Set:	LOD_LOQ_Linearity_Pyridine_Met
Injection #:	2	Date Processed:	11.02.2023 13:09:07
Injection Volume:	100,00 ul	Processing Method:	Linearity_Pyridine_M_Methoxy
Run Time:	20,0 Minutes	Channel Name:	Extract 275,0
Acquired By:	Roxana_Roman	Proc. Chnl. Descr.:	PDA 275,0 nm, Smoothed by 25

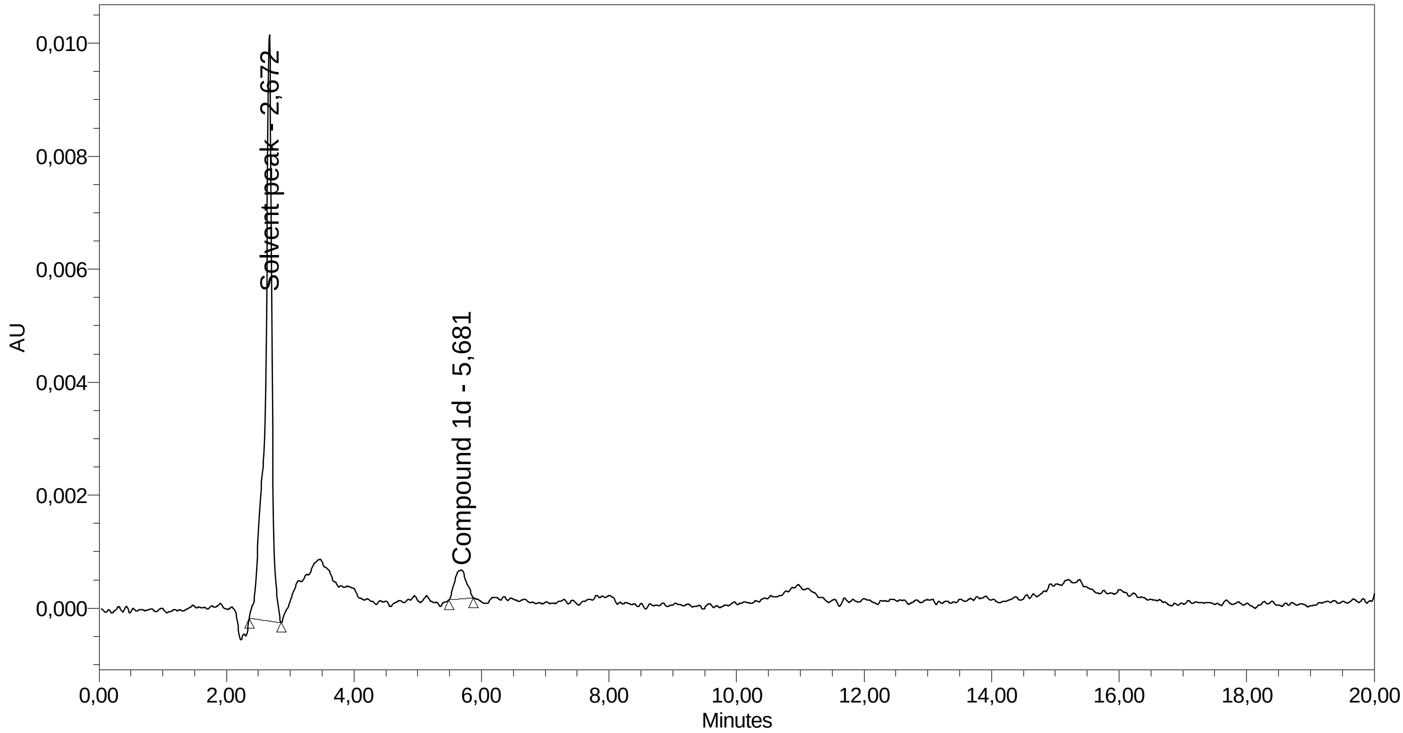

	Peak Name	RT	Area	Height (µV)	Level
1	Solvent peak	2,672	77109	10391	8
2	Compound 1d	5,681	6552	512	8

LOD LOQ_1d_Report

Reported by User: Roxana Roman (Roxana_Roman)
Acquisition Server Waters7
Project Name: Test
Sample Set Name: LOD_LOQ_Linearity_Pyridine Met
Code column: Inertsil ODS-3, 4,6*250 mm, 5 um

SAMPLE INFORMATION

Sample Name:	Solution 0.1 ug/ mL		
Sample Type:	Standard	Date Acquired:	11.06.2022 14:09:14
Vial:	6	Acq. Method Set:	LOD_LOQ_Linearity_Pyridine_Met
Injection #:	1	Date Processed:	11.02.2023 13:12:53
Injection Volume:	100,00 ul	Processing Method:	Linearity_Pyridine_M_Methoxy
Run Time:	20,0 Minutes	Channel Name:	Extract 275,0
Acquired By:	Roxana_Roman	Proc. Chnl. Descr.:	PDA 275,0 nm, Smoothed by 25

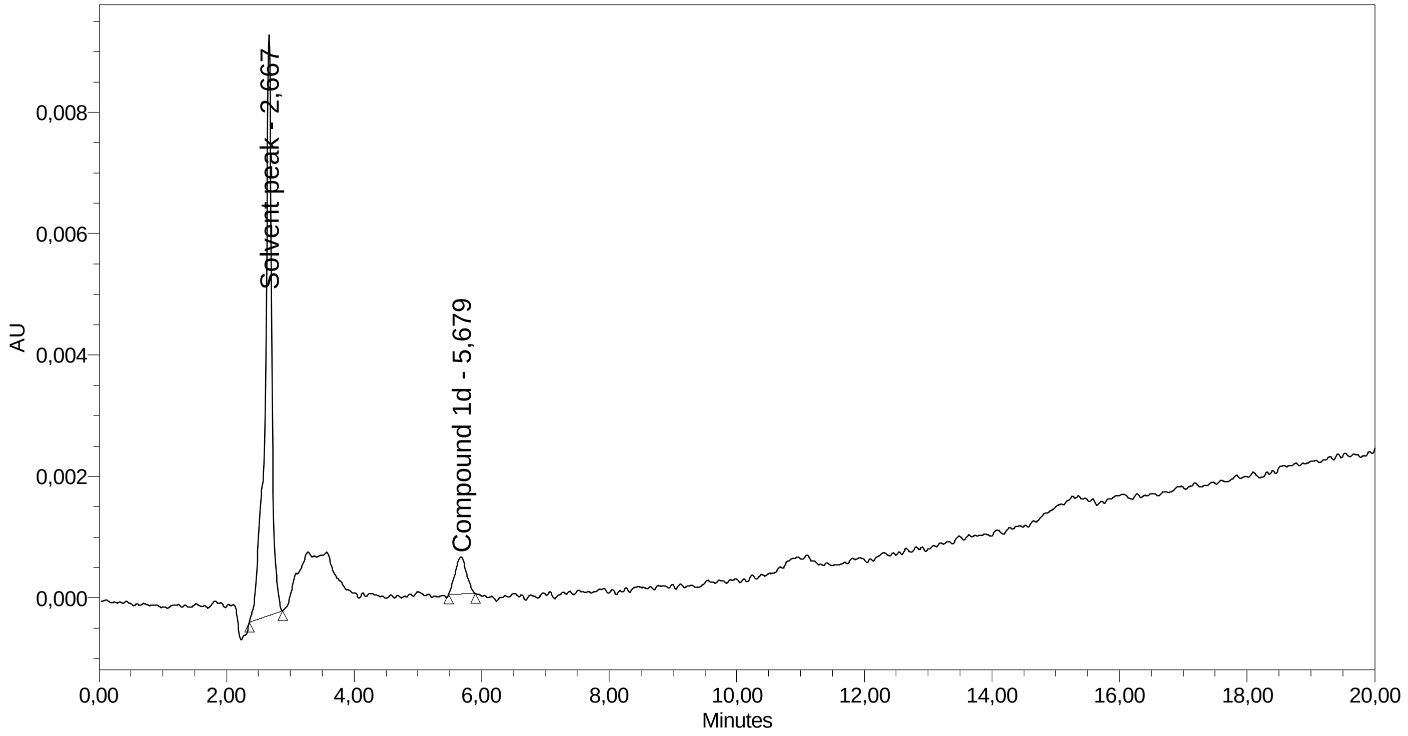

	Peak Name	RT	Area	Height (µV)	Level
1	Solvent peak	2,667	69780	9557	10
2	Compound 1d	5,679	7545	614	10

LOD LOQ_1d_Report

Reported by User: Roxana Roman (Roxana_Roman)
Acquisition Server Waters7
Project Name: Test
Sample Set Name: LOD_LOQ_Linearity_Pyridine Met
Code column: Inertsil ODS-3, 4,6*250 mm, 5 um

SAMPLE INFORMATION

Sample Name:	Solution 0.1 ug/ mL		
Sample Type:	Standard	Date Acquired:	11.06.2022 14:29:58
Vial:	6	Acq. Method Set:	LOD_LOQ_Linearity_Pyridine_Met
Injection #:	2	Date Processed:	11.02.2023 13:08:42
Injection Volume:	100,00 ul	Processing Method:	Linearity_Pyridine_M_Methoxy
Run Time:	20,0 Minutes	Channel Name:	Extract 275,0
Acquired By:	Roxana_Roman	Proc. Chnl. Descr.:	PDA 275,0 nm, Smoothed by 25

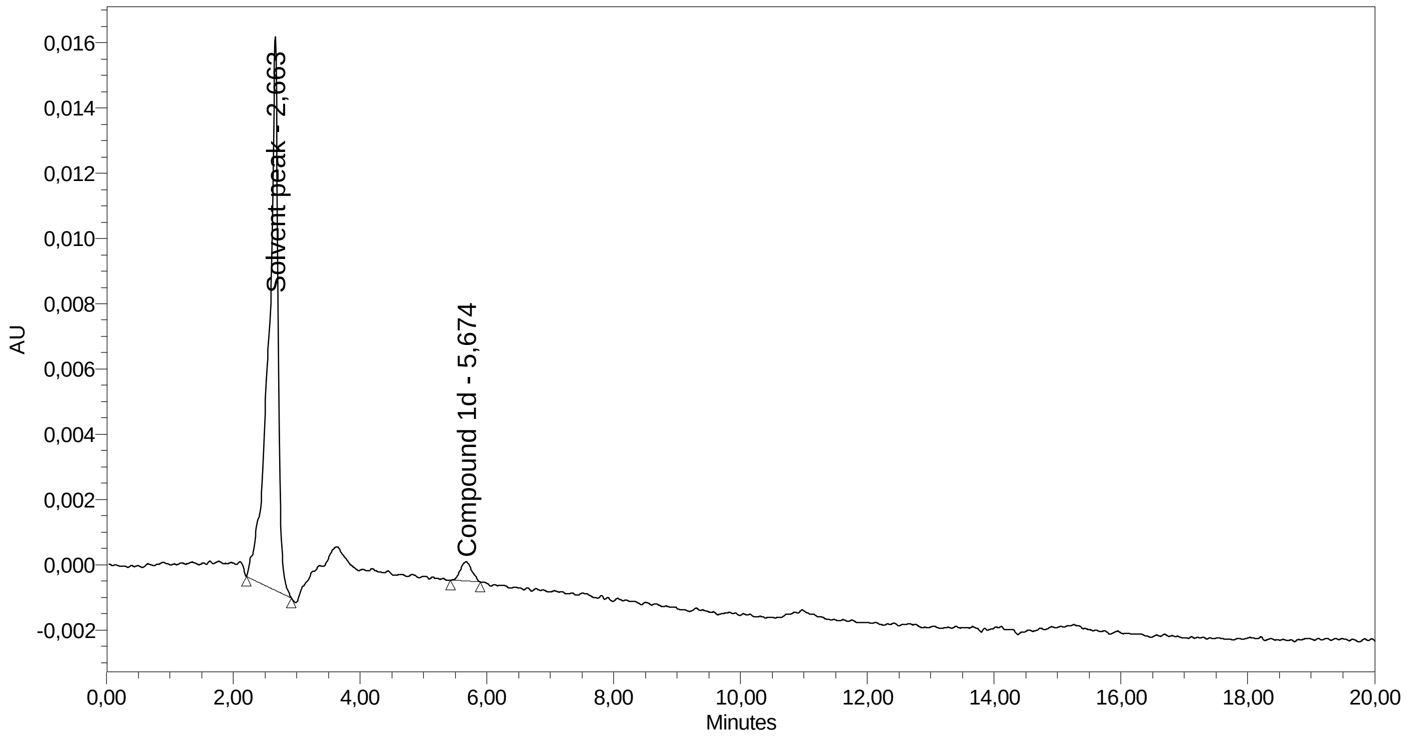

	Peak Name	RT	Area	Height (µV)	Level
1	Solvent peak	2,663	176979	17000	10
2	Compound 1d	5,674	7346	585	10

LOD LOQ_1d_Report

Reported by User: Roxana Roman (Roxana_Roman)
Acquisition Server Waters7
Project Name: Test
Sample Set Name: LOD_LOQ_Linearity_Pyridine Met
Code column: Inertsil ODS-3, 4,6*250 mm, 5 um

SAMPLE INFORMATION

Sample Name:	Solution 0.2 ug/ mL		
Sample Type:	Standard	Date Acquired:	11.06.2022 14:50:45
Vial:	7	Acq. Method Set:	LOD_LOQ_Linearity_Pyridine_Met
Injection #:	1	Date Processed:	11.02.2023 13:08:06
Injection Volume:	100,00 ul	Processing Method:	Linearity_Pyridine_M_Methoxy
Run Time:	20,0 Minutes	Channel Name:	Extract 275,0
Acquired By:	Roxana_Roman	Proc. Chnl. Descr.:	PDA 275,0 nm, Smoothed by 25

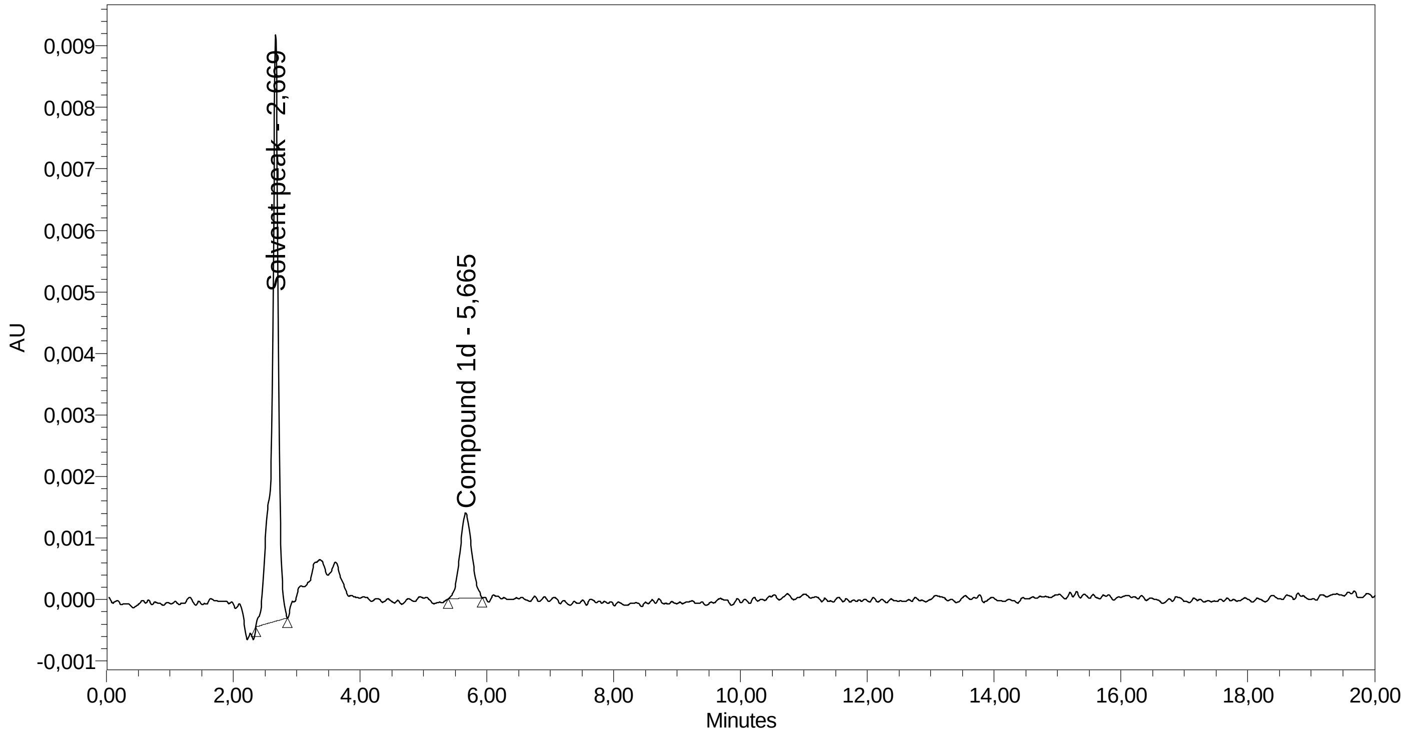

	Peak Name	RT	Area	Height (µV)	Level
1	Solvent peak	2,669	67923	9546	20
2	Compound 1d	5,665	17849	1384	20

LOD LOQ_1d_Report

Reported by User: Roxana Roman (Roxana_Roman)
Acquisition Server Waters7
Project Name: Test
Sample Set Name: LOD_LOQ_Linearity_Pyridine Met
Code column: Inertsil ODS-3, 4,6*250 mm, 5 um

SAMPLE INFORMATION

Sample Name:	Solution 0.2 ug/ mL		
Sample Type:	Standard	Date Acquired:	11.06.2022 15:11:29
Vial:	7	Acq. Method Set:	LOD_LOQ_Linearity_Pyridine_Met
Injection #:	2	Date Processed:	11.02.2023 13:07:55
Injection Volume:	100,00 ul	Processing Method:	Linearity_Pyridine_M_Methoxy
Run Time:	20,0 Minutes	Channel Name:	Extract 275,0
Acquired By:	Roxana_Roman	Proc. Chnl. Descr.:	PDA 275,0 nm, Smoothed by 25

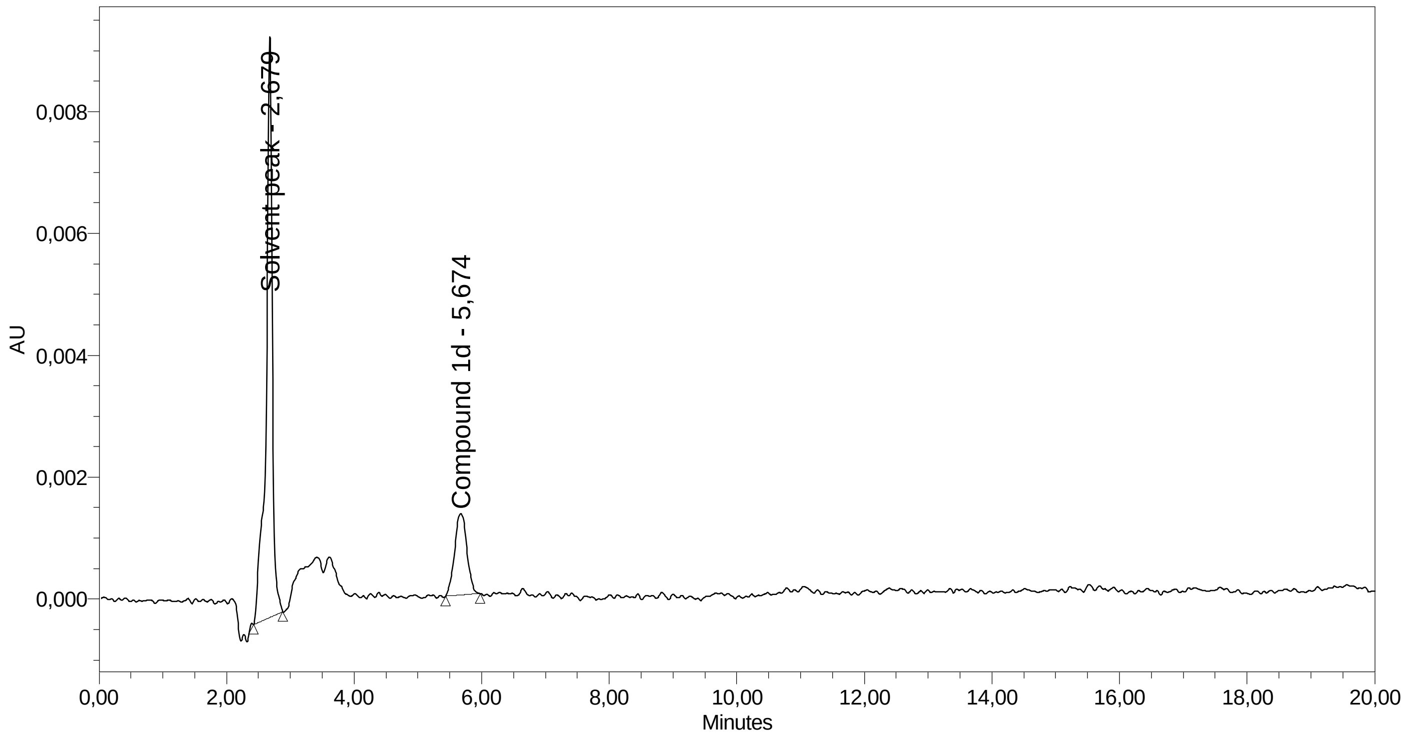

	Peak Name	RT	Area	Height (µV)	Level
1	Solvent peak	2,679	64525	9571	20
2	Compound 1d	5,674	17837	1333	20

Quantitative determination of

compound 1d

- Validation of the analytical method -

Validation parameters:

- Specificity
- Precision 1
- Precision 2
- LOD – LOQ
- **Linearity**
- **Range**
- Accuracy

Empower software

Linearity Calibration Curve

Reported by User: Roxana Roman (Roxana_Roman)
Acquisition Server Waters7
Project Name: Test
Sample Set Name: LOD_LOQ_Linearity_Pyridine Met
Code column: Inertsil ODS-3, 250*4.6 mm, 5 um
[illegible]

Calibration Plot

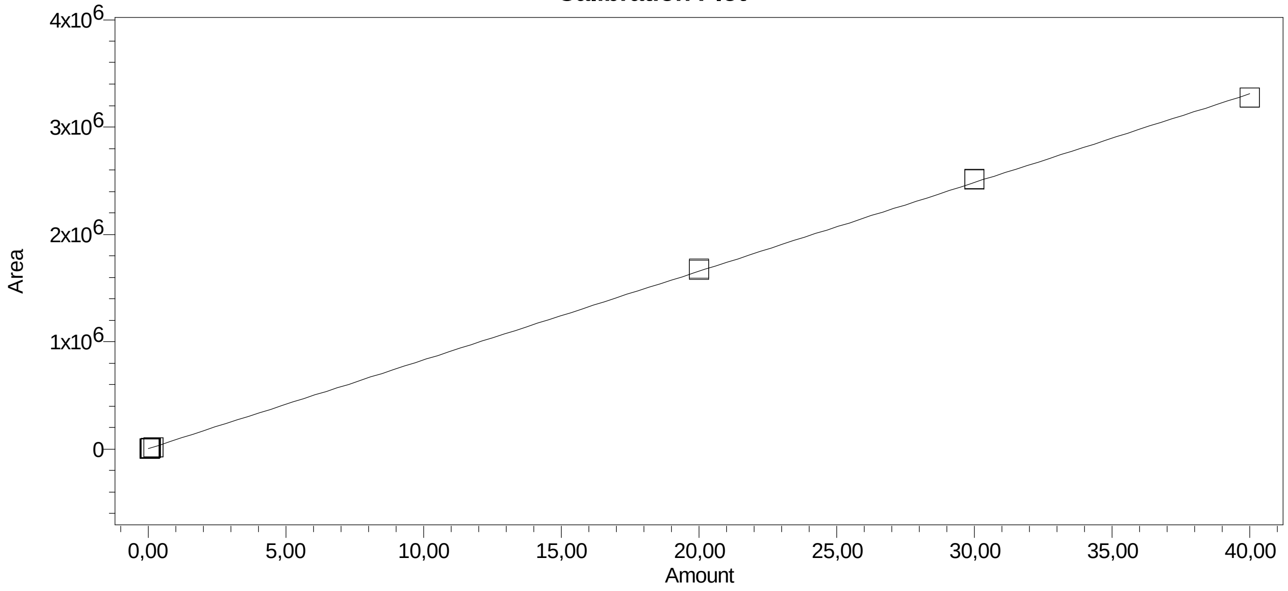

Name: Compound 1d; Processing Method: Linearity_Pyridine_M_Methoxy; Fit Type: Linear (1st Order); Cal Curve Id: 4844; A: 3,663288e+003; B: 8,265387e+004; C: 0,000000e+000; D: 0,000000e+000; R^2: 0,999788; Standard Error 1,972172e+004

Calibration Plot group for Solvent peak contains no data.

Peak: Compound 1d

	Name	Level	X Value	Response	Calc. Value	% Deviation	Manual	Ignore
1	Compound 1d	5	0,050000	4071,743656	0,004942	-90,116	No	No
2	Compound 1d	5	0,050000	3997,911266	0,004048	-91,903	No	No
3	Compound 1d	6	0,060000	4604,067650	0,011382	-81,030	No	No
4	Compound 1d	6	0,060000	4710,043738	0,012664	-78,893	No	No
5	Compound 1d	8	0,080000	6405,660466	0,033179	-58,526	No	No
6	Compound 1d	8	0,080000	6552,166667	0,034952	-56,311	No	No
7	Compound 1d	10	0,100000	7544,638514	0,046959	-53,041	No	No
8	Compound 1d	10	0,100000	7346,426958	0,044561	-55,439	No	No
9	Compound 1d	20	0,200000	17849,038191	0,171628	-14,186	No	No
10	Compound 1d	20	0,200000	17837,293510	0,171486	-14,257	No	No
11	Compound 1d	2000	20,000000	1671583,544206	20,179577	0,898	No	No
12	Compound 1d	2000	20,000000	1683469,718450	20,323384	1,617	No	No
13	Compound 1d	3000	30,000000	2510959,078862	30,334885	1,116	No	No
14	Compound 1d	3000	30,000000	2518985,651199	30,431996	1,440	No	No
15	Compound 1d	4000	40,000000	3274980,767505	39,578513	-1,054	No	No
16	Compound 1d	4000	40,000000	3276413,074307	39,595842	-1,010	No	No

Linearity Calibration Curve

Reported by User: Roxana Roman (Roxana_Roman)
Acquisition Server Waters7
Project Name: Test
Sample Set Name: LOD_LOQ_Linearity_Pyridine Met
Code column: Inertsil ODS-3, 250*4.6 mm, 5 um
[illegible]

Point Information ' Peak: Solvent peak' table contains no data.

Seria Metoxi_1d_Linearity

Reported by User: Roxana Roman (Roxana_Roman)
Acquisition Server Waters7
Project Name: Test
Sample Set Name: LOD_LOQ_Linearity_Pyridine Met
Code column: Inertsil ODS-3, 4,6*250 mm, 5 um

SAMPLE INFORMATION

Sample Name:	Solution 0.05 ug/ mL		
Sample Type:	Standard	Date Acquired:	11.06.2022 12:45:55
Vial:	3	Acq. Method Set:	LOD_LOQ_Linearity_Pyridine_Met
Injection #:	1	Date Processed:	20.02.2023 10:58:16
Injection Volume:	100,00 ul	Processing Method:	Linearity_Pyridine_M_Methoxy
Run Time:	20,0 Minutes	Channel Name:	Extract 275,0
Acquired By:	Roxana_Roman	Proc. Chnl. Descr.:	PDA 275,0 nm, Smoothed by 25

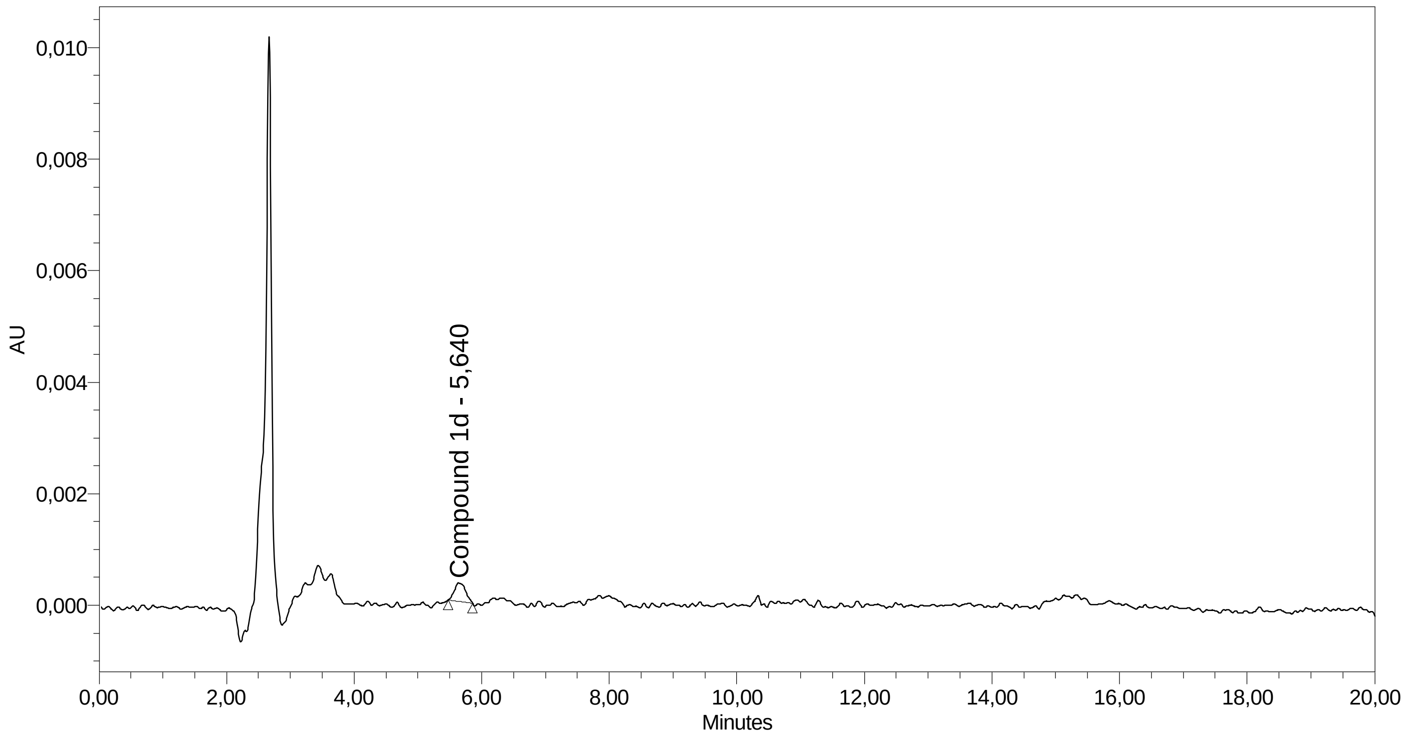

	Peak Name	RT	Area	Height (µV)	Level
1	Compound 1d	5,640	4072	328	5

Seria Metoxi_1d_Linearity

Reported by User: Roxana Roman (Roxana_Roman)
Acquisition Server Waters7
Project Name: Test
Sample Set Name: LOD_LOQ_Linearity_Pyridine Met
Code column: Inertsil ODS-3, 4,6*250 mm, 5 um

SAMPLE INFORMATION

Sample Name:	Solution 0.05 ug/ mL		
Sample Type:	Standard	Date Acquired:	11.06.2022 13:06:54
Vial:	3	Acq. Method Set:	LOD_LOQ_Linearity_Pyridine_Met
Injection #:	2	Date Processed:	20.02.2023 10:58:43
Injection Volume:	100,00 ul	Processing Method:	Linearity_Pyridine_M_Methoxy
Run Time:	20,0 Minutes	Channel Name:	Extract 275,0
Acquired By:	Roxana_Roman	Proc. Chnl. Descr.:	PDA 275,0 nm, Smoothed by 25

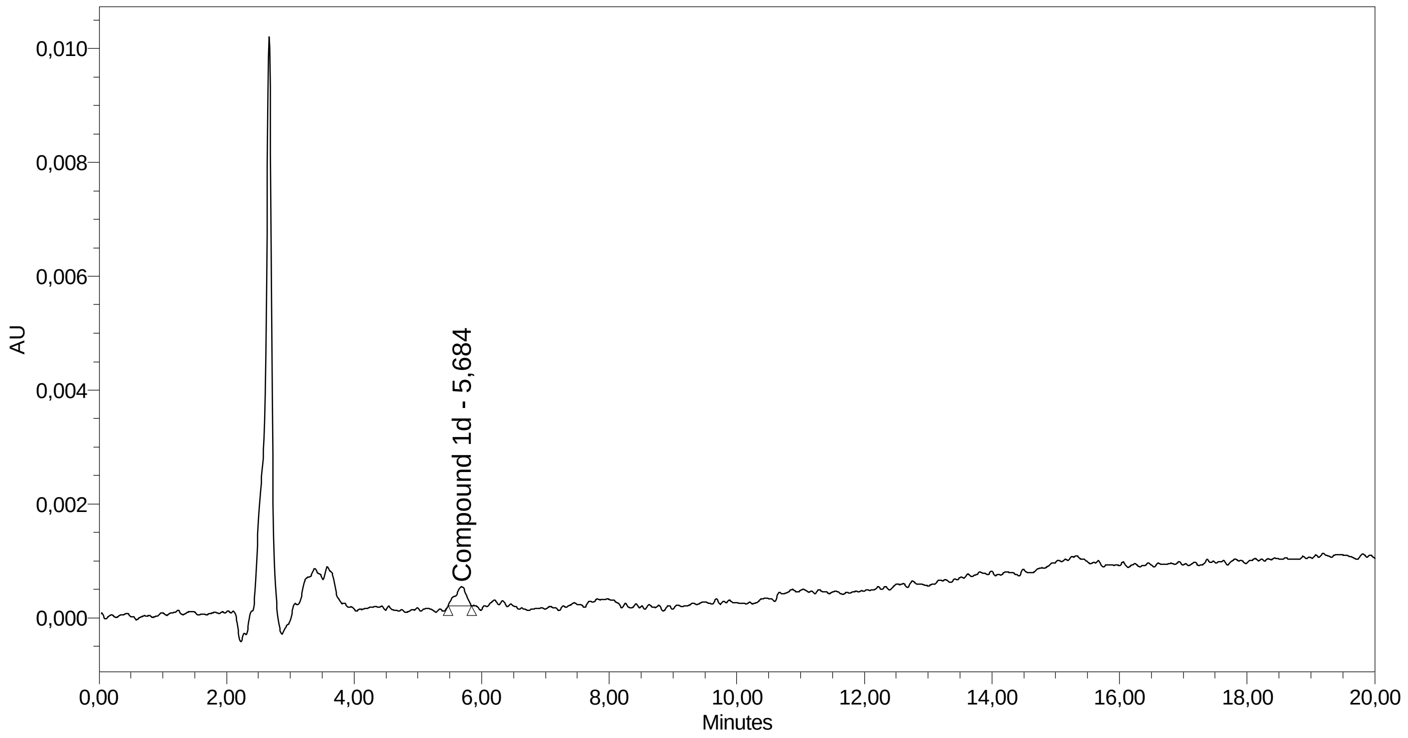

	Peak Name	RT	Area	Height (µV)	Level
1	Compound 1d	5,684	3998	337	5

Seria Metoxi_1d_Linearity

Reported by User: Roxana Roman (Roxana_Roman)
Acquisition Server Waters7
Project Name: Test
Sample Set Name: LOD_LOQ_Linearity_Pyridine Met
Code column: Inertsil ODS-3, 4,6*250 mm, 5 um

SAMPLE INFORMATION

Sample Name:	Solution 0.06 ug/ mL		
Sample Type:	Standard	Date Acquired:	11.06.2022 12:03:47
Vial:	4	Acq. Method Set:	LOD_LOQ_Linearity_Pyridine_Met
Injection #:	1	Date Processed:	20.02.2023 10:59:18
Injection Volume:	100,00 ul	Processing Method:	Linearity_Pyridine_M_Methoxy
Run Time:	20,0 Minutes	Channel Name:	Extract 275,0
Acquired By:	Roxana_Roman	Proc. Chnl. Descr.:	PDA 275,0 nm, Smoothed by 25

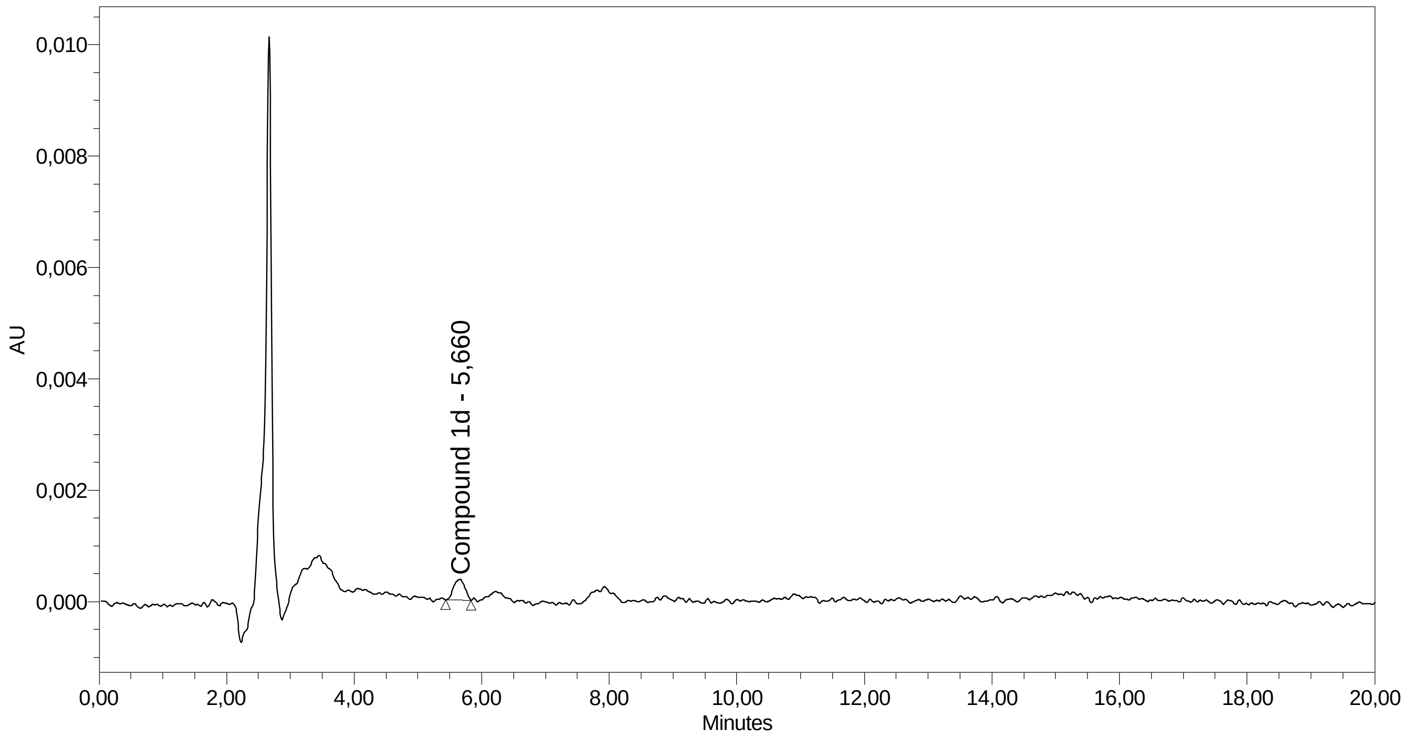

	Peak Name	RT	Area	Height (µV)	Level
1	Compound 1d	5,660	4604	378	6

Seria Metoxi_1d_Linearity

Reported by User: Roxana Roman (Roxana_Roman)
Acquisition Server Waters7
Project Name: Test
Sample Set Name: LOD_LOQ_Linearity_Pyridine Met
Code column: Inertsil ODS-3, 4,6*250 mm, 5 um

S A M P L E   I N F O R M A T I O N

Sample Name:	Solution 0.06 ug/ mL		
Sample Type:	Standard	Date Acquired:	11.06.2022 12:24:32
Vial:	4	Acq. Method Set:	LOD_LOQ_Linearity_Pyridine_Met
Injection #:	2	Date Processed:	20.02.2023 10:59:39
Injection Volume:	100,00 ul	Processing Method:	Linearity_Pyridine_M_Methoxy
Run Time:	20,0 Minutes	Channel Name:	Extract 275,0
Acquired By:	Roxana_Roman	Proc. Chnl. Descr.:	PDA 275,0 nm, Smoothed by 25

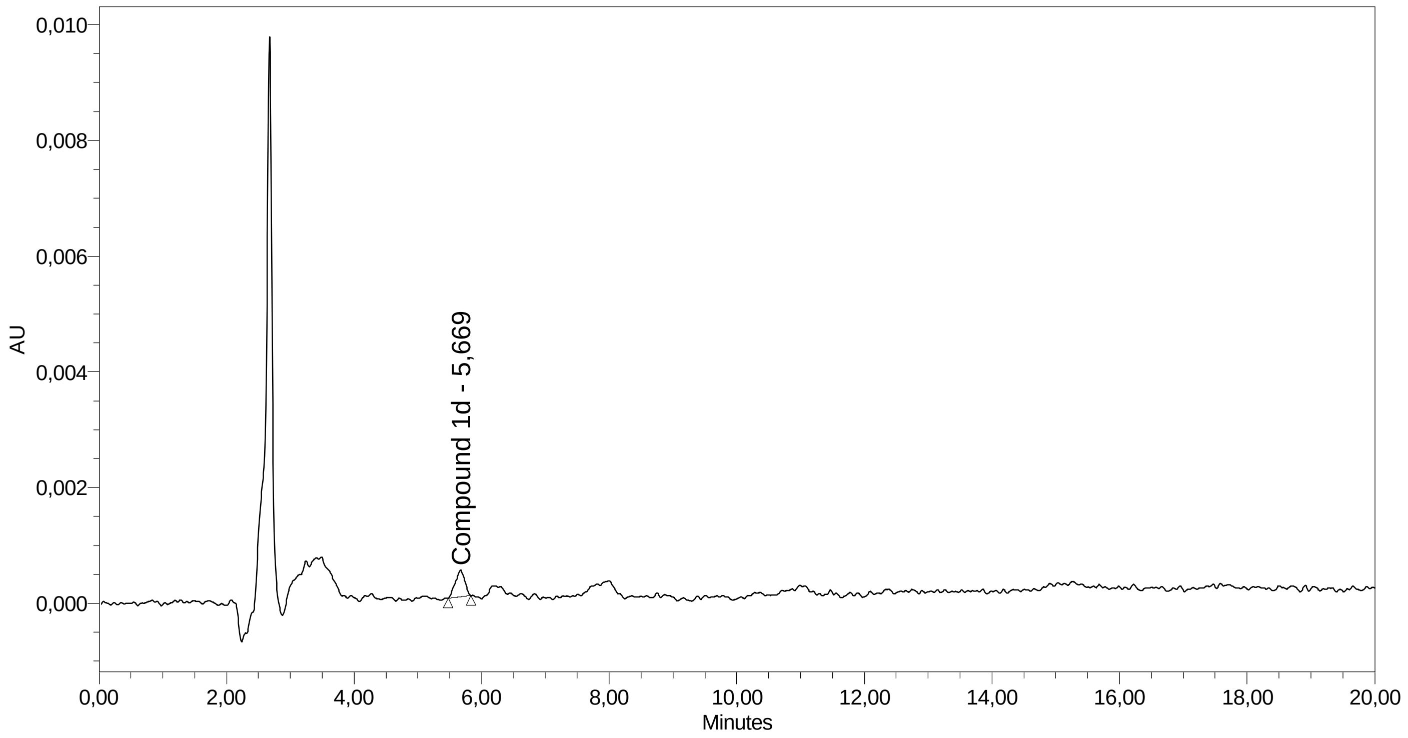

	Peak Name	RT	Area	Height (µV)	Level
1	Compound 1d	5,669	4710	462	6

Seria Metoxi_1d_Linearity

Reported by User: Roxana Roman (Roxana_Roman)
Acquisition Server Waters7
Project Name: Test
Sample Set Name: LOD_LOQ_Linearity_Pyridine Met
Code column: Inertsil ODS-3, 4,6*250 mm, 5 um

SAMPLE INFORMATION

Sample Name:	Solution 0.08 ug/ mL		
Sample Type:	Standard	Date Acquired:	11.06.2022 13:27:42
Vial:	5	Acq. Method Set:	LOD_LOQ_Linearity_Pyridine_Met
Injection #:	1	Date Processed:	20.02.2023 11:00:40
Injection Volume:	100,00 ul	Processing Method:	Linearity_Pyridine_M_Methoxy
Run Time:	20,0 Minutes	Channel Name:	Extract 275,0
Acquired By:	Roxana_Roman	Proc. Chnl. Descr.:	PDA 275,0 nm, Smoothed by 25

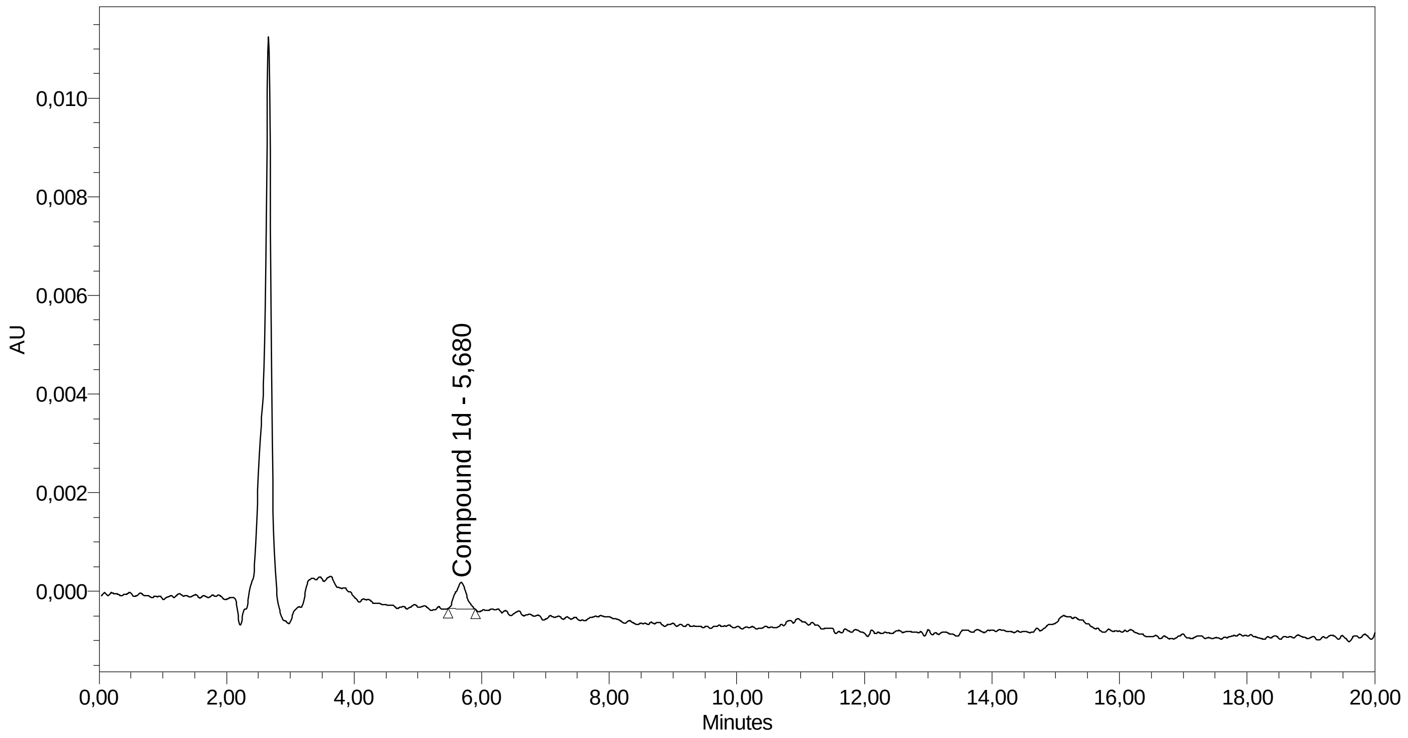

	Peak Name	RT	Area	Height (µV)	Level
1	Compound 1d	5,680	6406	539	8

Seria Metoxi_1d_Linearity

Reported by User: Roxana Roman (Roxana_Roman)
Acquisition Server Waters7
Project Name: Test
Sample Set Name: LOD_LOQ_Linearity_Pyridine Met
Code column: Inertsil ODS-3, 4,6*250 mm, 5 um

SAMPLE INFORMATION

Sample Name:	Solution 0.08 ug/ mL		
Sample Type:	Standard	Date Acquired:	11.06.2022 13:48:26
Vial:	5	Acq. Method Set:	LOD_LOQ_Linearity_Pyridine_Met
Injection #:	2	Date Processed:	20.02.2023 11:02:22
Injection Volume:	100,00 ul	Processing Method:	Linearity_Pyridine_M_Methoxy
Run Time:	20,0 Minutes	Channel Name:	Extract 275,0
Acquired By:	Roxana_Roman	Proc. Chnl. Descr.:	PDA 275,0 nm, Smoothed by 25

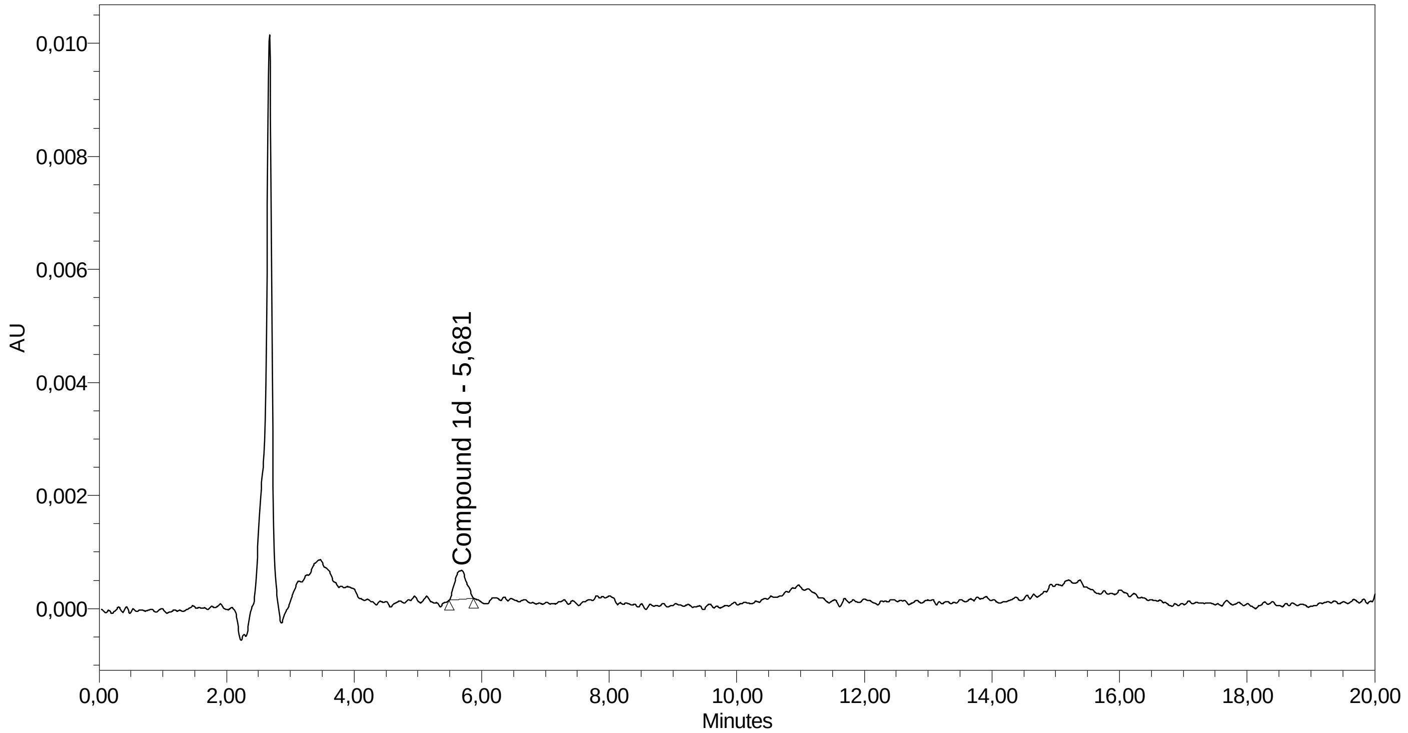

	Peak Name	RT	Area	Height (µV)	Level
1	Compound 1d	5,681	6552	512	8

Seria Metoxi_1d_Linearity

Reported by User: Roxana Roman (Roxana_Roman)
Acquisition Server Waters7
Project Name: Test
Sample Set Name: LOD_LOQ_Linearity_Pyridine Met
Code column: Inertsil ODS-3, 4,6*250 mm, 5 um

S A M P L E   I N F O R M A T I O N

Sample Name:	Solution 0.1 ug/ mL		
Sample Type:	Standard	Date Acquired:	11.06.2022 14:09:14
Vial:	6	Acq. Method Set:	LOD_LOQ_Linearity_Pyridine_Met
Injection #:	1	Date Processed:	20.02.2023 10:54:39
Injection Volume:	100,00 ul	Processing Method:	Linearity_Pyridine_M_Methoxy
Run Time:	20,0 Minutes	Channel Name:	Extract 275,0
Acquired By:	Roxana_Roman	Proc. Chnl. Descr.:	PDA 275,0 nm, Smoothed by 25

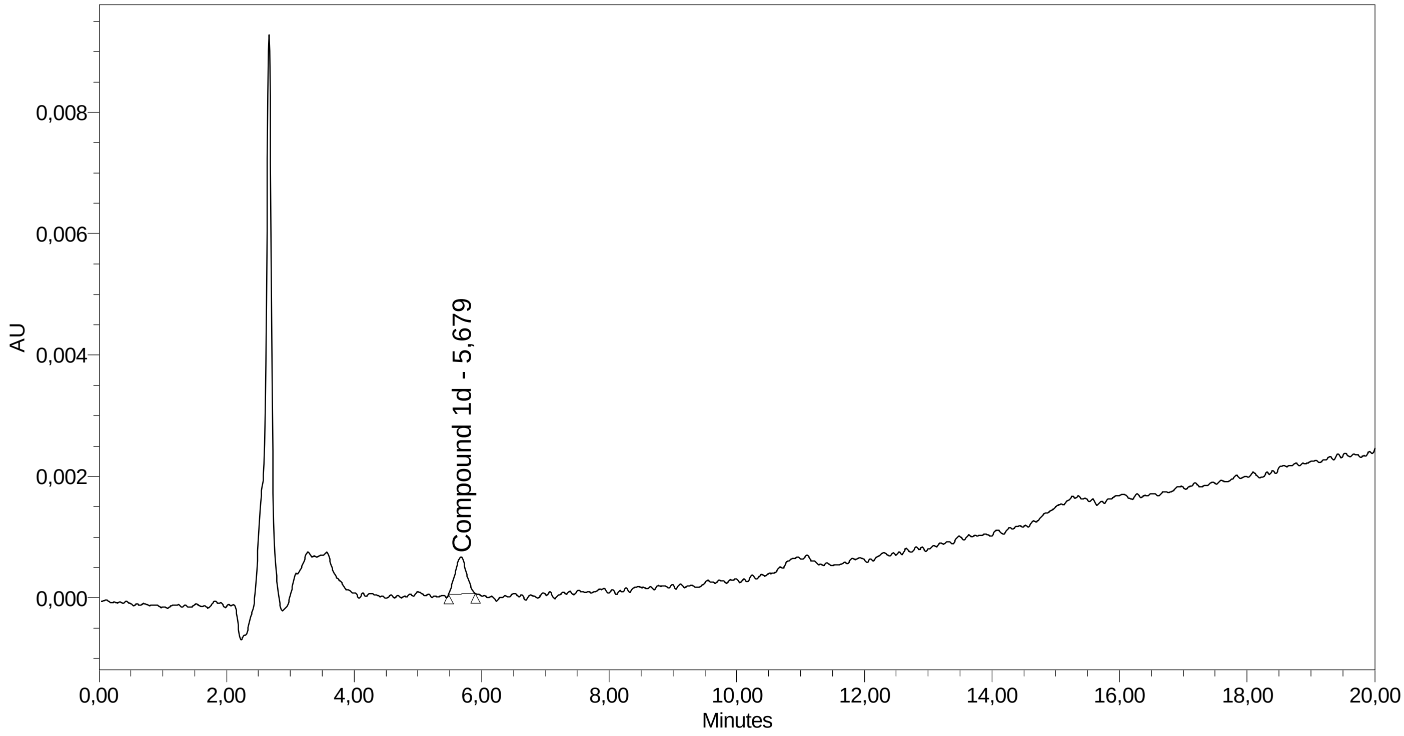

	Peak Name	RT	Area	Height (µV)	Level
1	Compound 1d	5,679	7545	614	10

Seria Metoxi_1d_Linearity

Reported by User: Roxana Roman (Roxana_Roman)
Acquisition Server Waters7
Project Name: Test
Sample Set Name: LOD_LOQ_Linearity_Pyridine Met
Code column: Inertsil ODS-3, 4,6*250 mm, 5 um

SAMPLE INFORMATION

Sample Name:	Solution 0.1 ug/ mL		
Sample Type:	Standard	Date Acquired:	11.06.2022 14:29:58
Vial:	6	Acq. Method Set:	LOD_LOQ_Linearity_Pyridine_Met
Injection #:	2	Date Processed:	20.02.2023 10:55:32
Injection Volume:	100,00 ul	Processing Method:	Linearity_Pyridine_M_Methoxy
Run Time:	20,0 Minutes	Channel Name:	Extract 275,0
Acquired By:	Roxana_Roman	Proc. Chnl. Descr.:	PDA 275,0 nm, Smoothed by 25

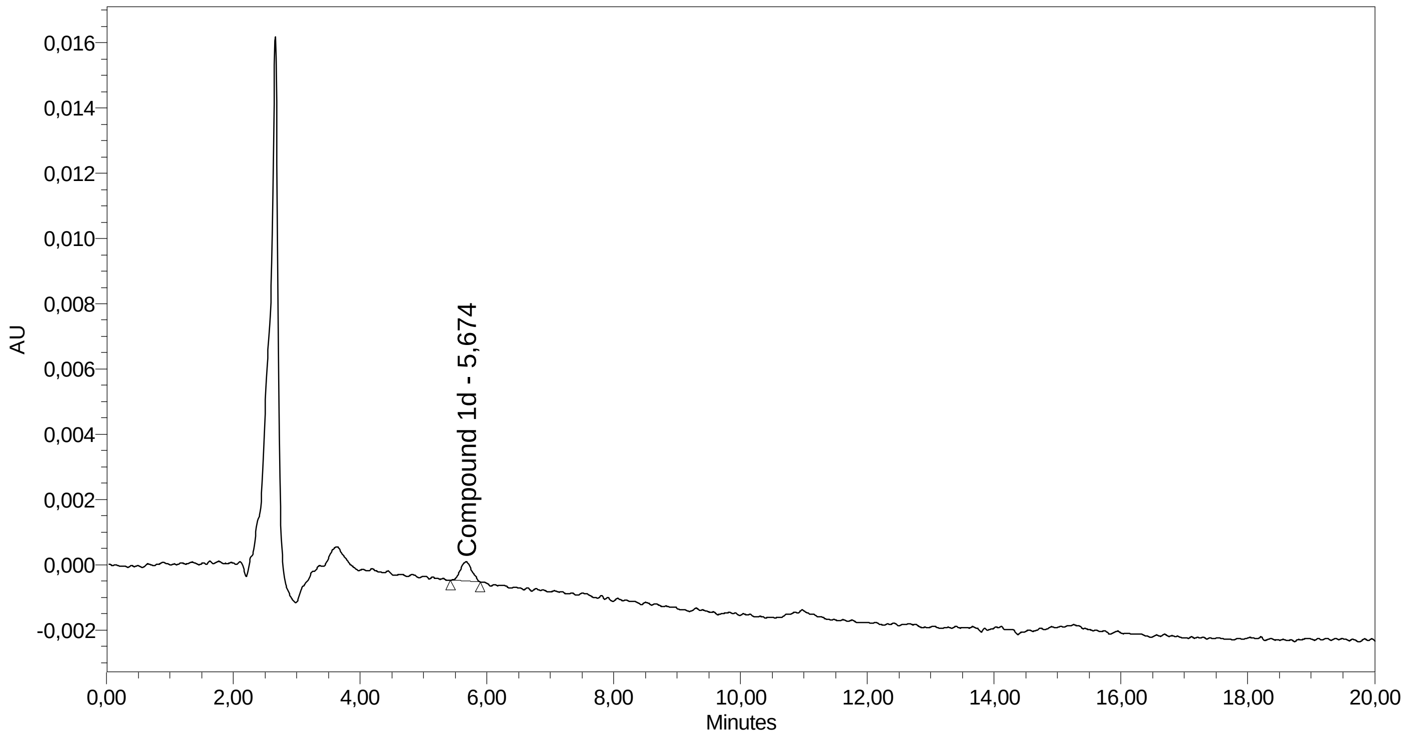

	Peak Name	RT	Area	Height (µV)	Level
1	Compound 1d	5,674	7346	585	10

Seria Metoxi_1d_Linearity

Reported by User: Roxana Roman (Roxana_Roman)
Acquisition Server Waters7
Project Name: Test
Sample Set Name: LOD_LOQ_Linearity_Pyridine Met
Code column: Inertsil ODS-3, 4,6*250 mm, 5 um

SAMPLE INFORMATION

Sample Name:	Solution 0.2 ug/ mL		
Sample Type:	Standard	Date Acquired:	11.06.2022 14:50:45
Vial:	7	Acq. Method Set:	LOD_LOQ_Linearity_Pyridine_Met
Injection #:	1	Date Processed:	20.02.2023 10:56:45
Injection Volume:	100,00 ul	Processing Method:	Linearity_Pyridine_M_Methoxy
Run Time:	20,0 Minutes	Channel Name:	Extract 275,0
Acquired By:	Roxana_Roman	Proc. Chnl. Descr.:	PDA 275,0 nm, Smoothed by 25

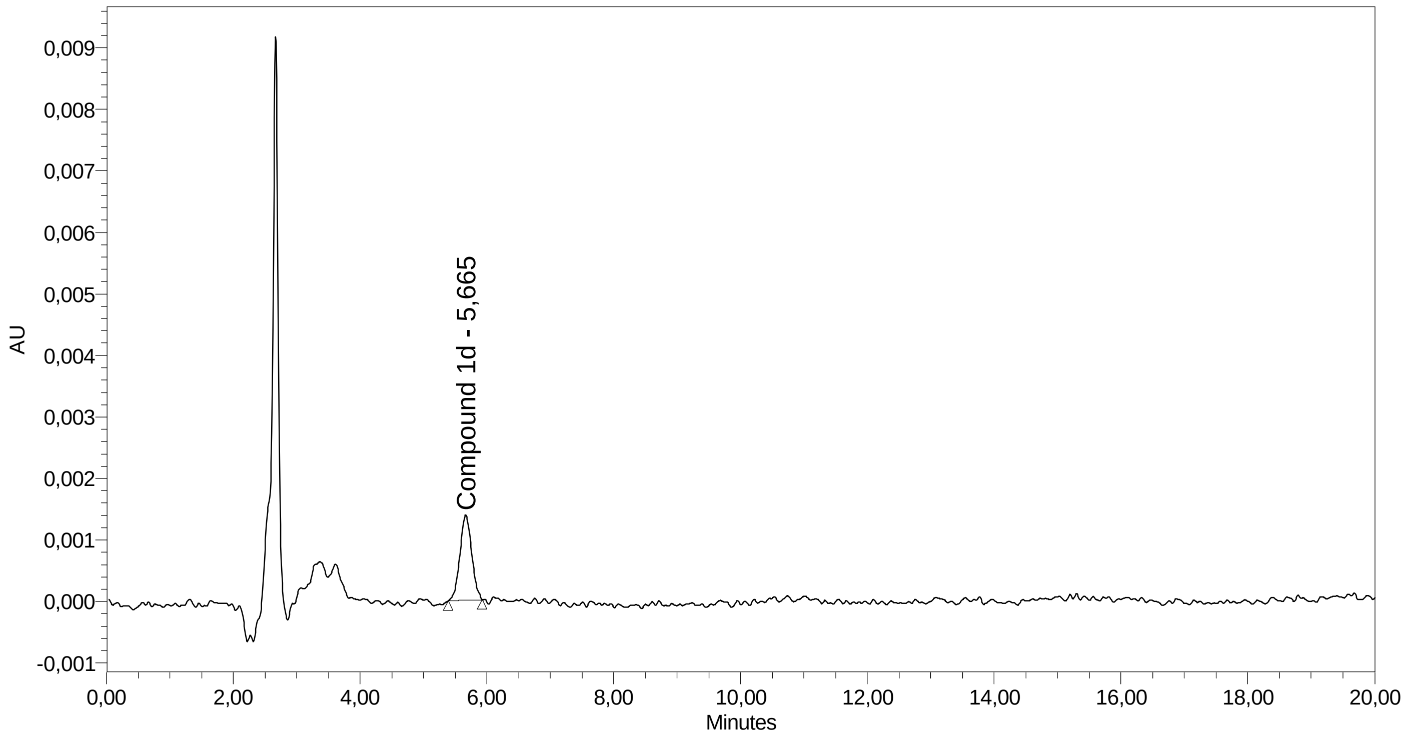

	Peak Name	RT	Area	Height (µV)	Level
1	Compound 1d	5,665	17849	1384	20

Seria Metoxi_1d_Linearity

Reported by User: Roxana Roman (Roxana_Roman)
Acquisition Server Waters7
Project Name: Test
Sample Set Name: LOD_LOQ_Linearity_Pyridine Met
Code column: Inertsil ODS-3, 4,6*250 mm, 5 um

SAMPLE INFORMATION

Sample Name:	Solution 0.2 ug/ mL		
Sample Type:	Standard	Date Acquired:	11.06.2022 15:11:29
Vial:	7	Acq. Method Set:	LOD_LOQ_Linearity_Pyridine_Met
Injection #:	2	Date Processed:	20.02.2023 10:57:34
Injection Volume:	100,00 ul	Processing Method:	Linearity_Pyridine_M_Methoxy
Run Time:	20,0 Minutes	Channel Name:	Extract 275,0
Acquired By:	Roxana_Roman	Proc. Chnl. Descr.:	PDA 275,0 nm, Smoothed by 25

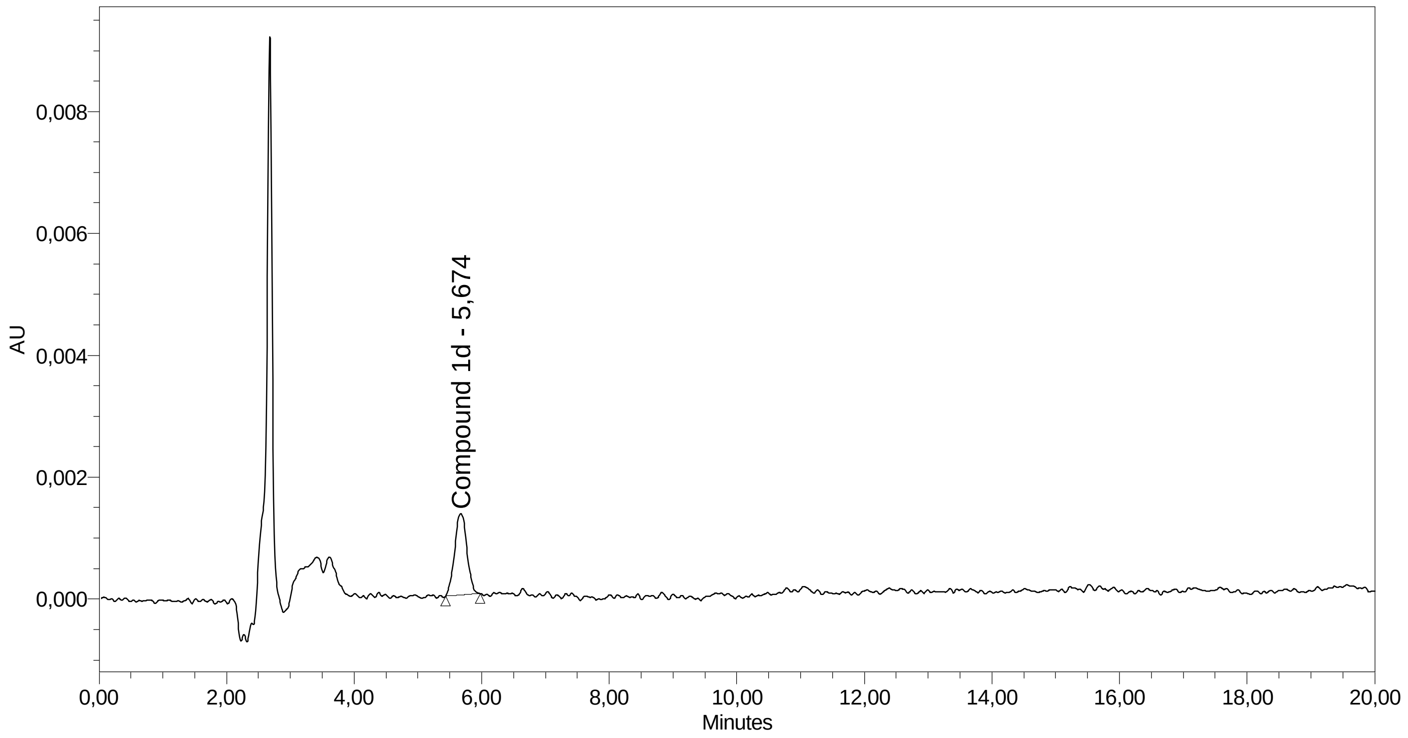

	Peak Name	RT	Area	Height (µV)	Level
1	Compound 1d	5,674	17837	1333	20

Seria Metoxi_1d_Linearity

Reported by User: Roxana Roman (Roxana_Roman)
Acquisition Server Waters7
Project Name: Test
Sample Set Name: Precision 1_Accuracy_PyridineM
Code column: Inertsil ODS-3, 4,6*250 mm, 5 um

SAMPLE INFORMATION

Sample Name:	Methyl-pyridine 20 ug/ mL		
Sample Type:	Standard	Date Acquired:	11.06.2022 20:16:34
Vial:	26	Acq. Method Set:	Precision 1_Accuracy_PyridineM
Injection #:	1	Date Processed:	20.02.2023 10:53:56
Injection Volume:	100,00 ul	Processing Method:	Linearity_Pyridine_M_Methoxy
Run Time:	20,0 Minutes	Channel Name:	Extract 275,0
Acquired By:	Roxana_Roman	Proc. Chnl. Descr.:	PDA 275,0 nm, Smoothed by 25

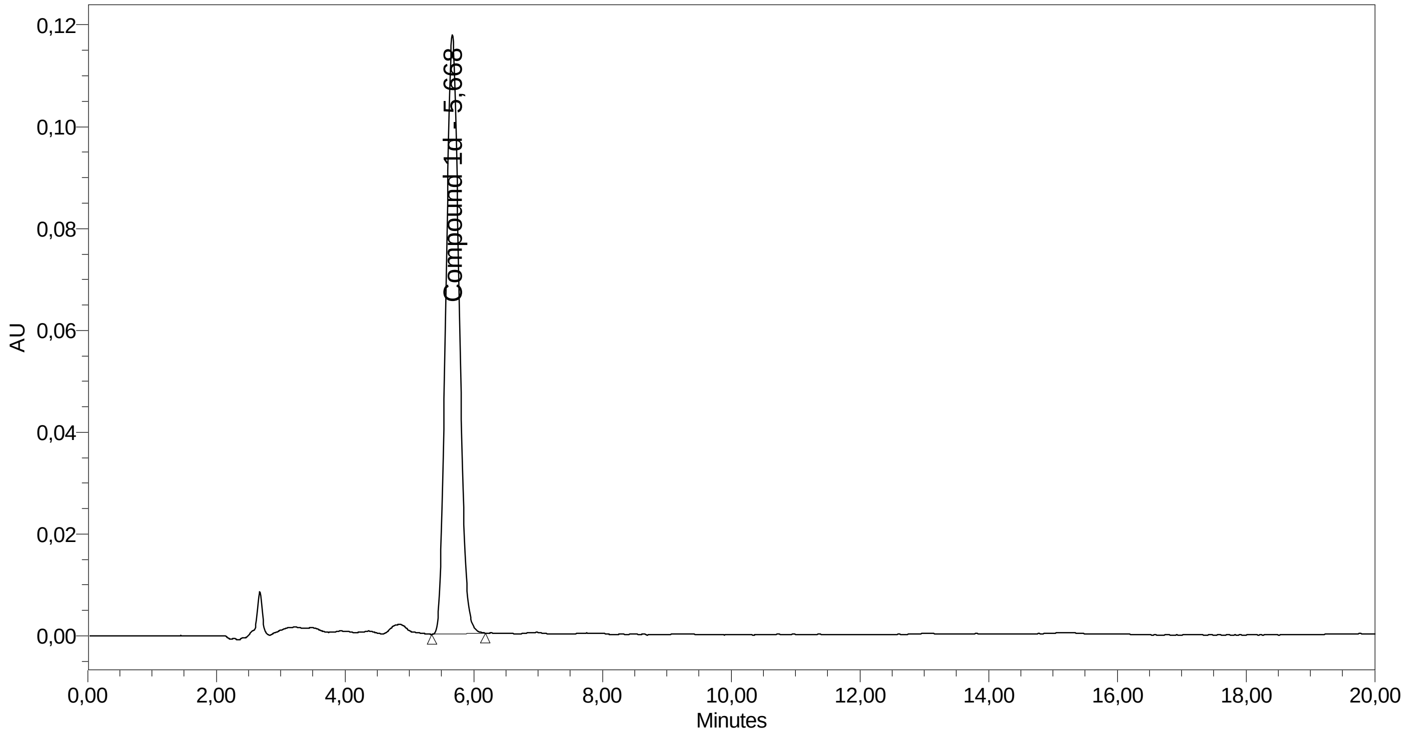

	Peak Name	RT	Area	Height (µV)	Level
1	Compound 1d	5,668	1683470	117640	2000

Seria Metoxi_1d_Linearity

Reported by User: Roxana Roman (Roxana_Roman)
Acquisition Server Waters7
Project Name: Test
Sample Set Name: Precision 1_Accuracy_PyridineM
Code column: Inertsil ODS-3, 4,6*250 mm, 5 um

SAMPLE INFORMATION

Sample Name:	Methyl-pyridine 20 ug/ mL		
Sample Type:	Standard	Date Acquired:	11.06.2022 20:37:21
Vial:	27	Acq. Method Set:	Precision 1_Accuracy_PyridineM
Injection #:	1	Date Processed:	20.02.2023 10:53:57
Injection Volume:	100,00 ul	Processing Method:	Linearity_Pyridine_M_Methoxy
Run Time:	20,0 Minutes	Channel Name:	Extract 275,0
Acquired By:	Roxana_Roman	Proc. Chnl. Descr.:	PDA 275,0 nm, Smoothed by 25

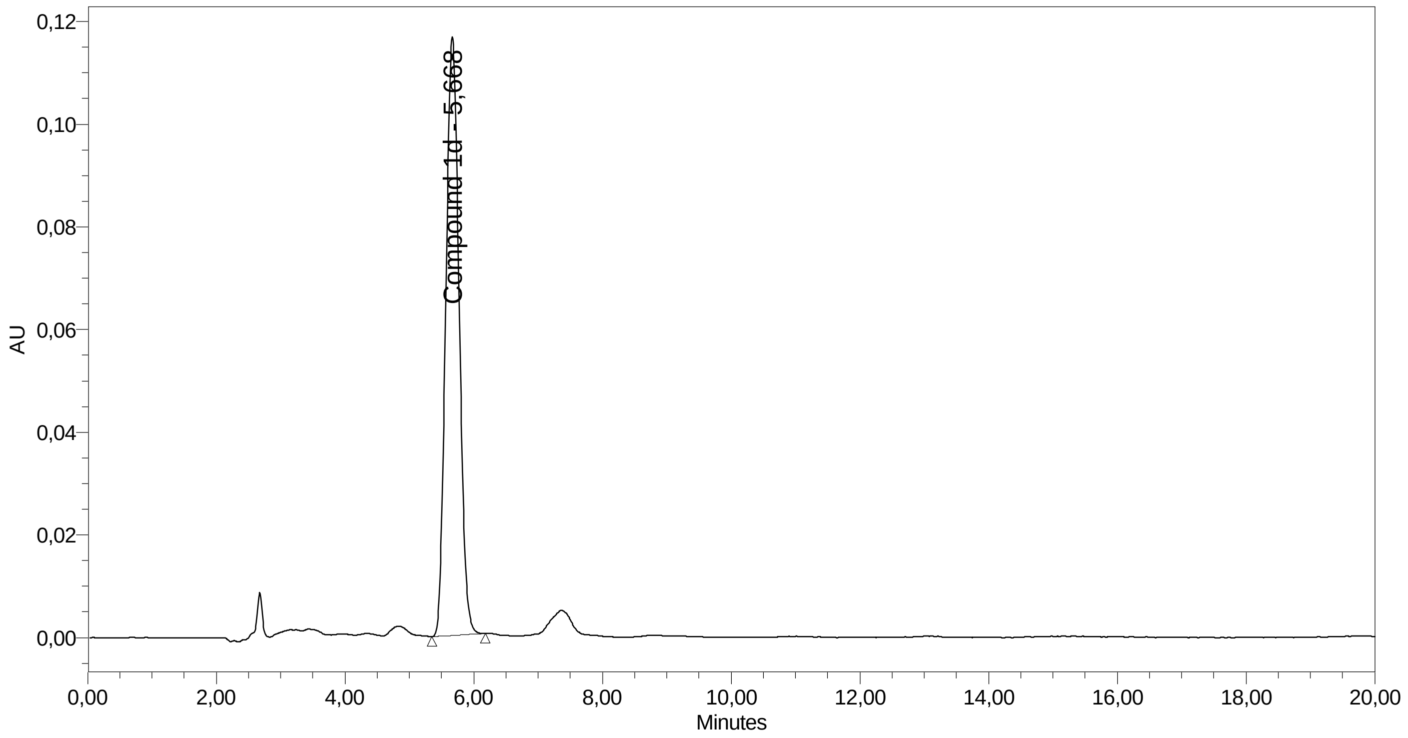

	Peak Name	RT	Area	Height (µV)	Level
1	Compound 1d	5,668	1671584	116577	2000

Seria Metoxi_1d_Linearity

Reported by User: Roxana Roman (Roxana_Roman)
Acquisition Server Waters7
Project Name: Test
Sample Set Name: Precision 1_Accuracy_PyridineM
Code column: Inertsil ODS-3, 4,6*250 mm, 5 um

SAMPLE INFORMATION

Sample Name:	Methyl-pyridine 30 ug/ mL		
Sample Type:	Standard	Date Acquired:	11.06.2022 22:21:21
Vial:	32	Acq. Method Set:	Precision 1_Accuracy_PyridineM
Injection #:	1	Date Processed:	20.02.2023 10:54:00
Injection Volume:	100,00 ul	Processing Method:	Linearity_Pyridine_M_Methoxy
Run Time:	20,0 Minutes	Channel Name:	Extract 275,0
Acquired By:	Roxana_Roman	Proc. Chnl. Descr.:	PDA 275,0 nm, Smoothed by 25

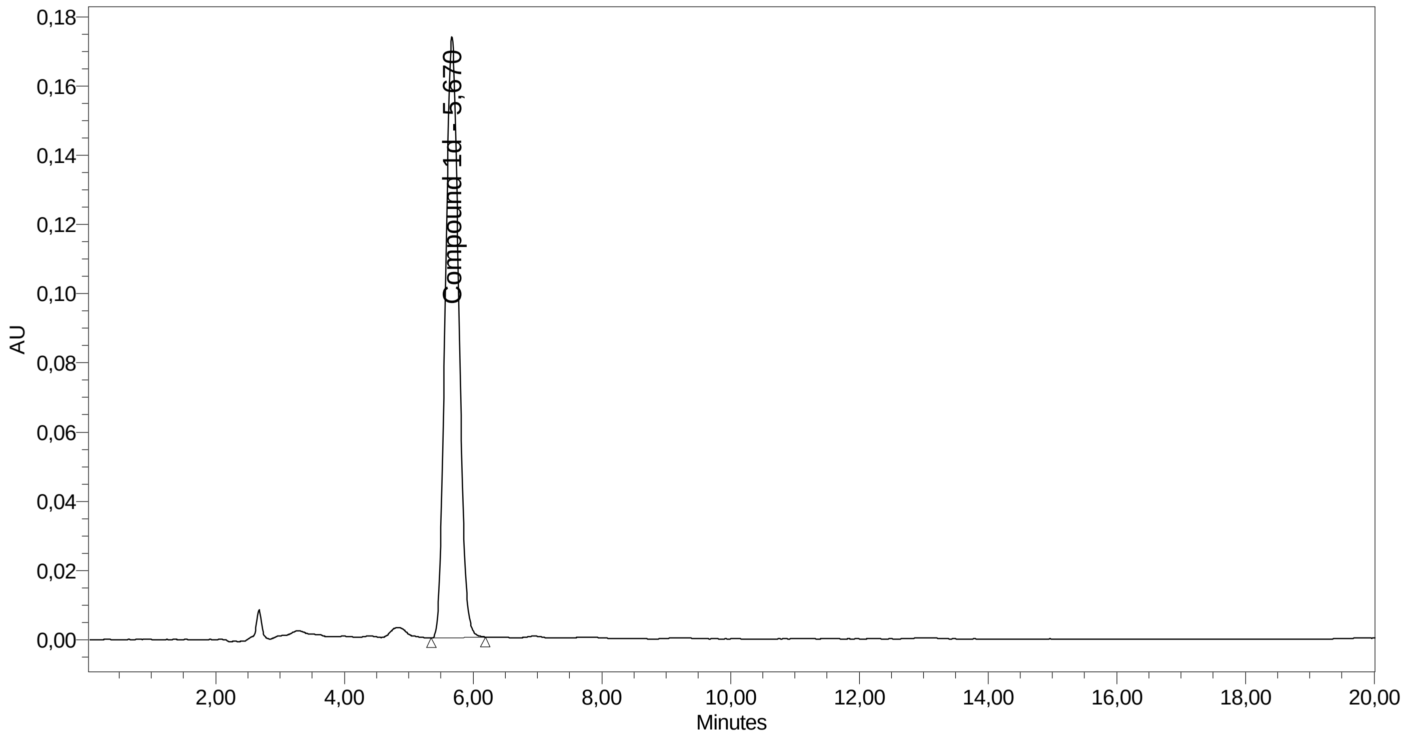

	Peak Name	RT	Area	Height (µV)	Level
1	Compound 1d	5,670	2518986	173649	3000

Seria Metoxi_1d_Linearity

Reported by User: Roxana Roman (Roxana_Roman)
Acquisition Server Waters7
Project Name: Test
Sample Set Name: Precision 1_Accuracy_PyridineM
Code column: Inertsil ODS-3, 4,6*250 mm, 5 um

SAMPLE INFORMATION

Sample Name:	Methyl-pyridine 30 ug/ mL		
Sample Type:	Standard	Date Acquired:	11.06.2022 22:42:09
Vial:	33	Acq. Method Set:	Precision 1_Accuracy_PyridineM
Injection #:	1	Date Processed:	20.02.2023 10:54:03
Injection Volume:	100,00 ul	Processing Method:	Linearity_Pyridine_M_Methoxy
Run Time:	20,0 Minutes	Channel Name:	Extract 275,0
Acquired By:	Roxana_Roman	Proc. Chnl. Descr.:	PDA 275,0 nm, Smoothed by 25

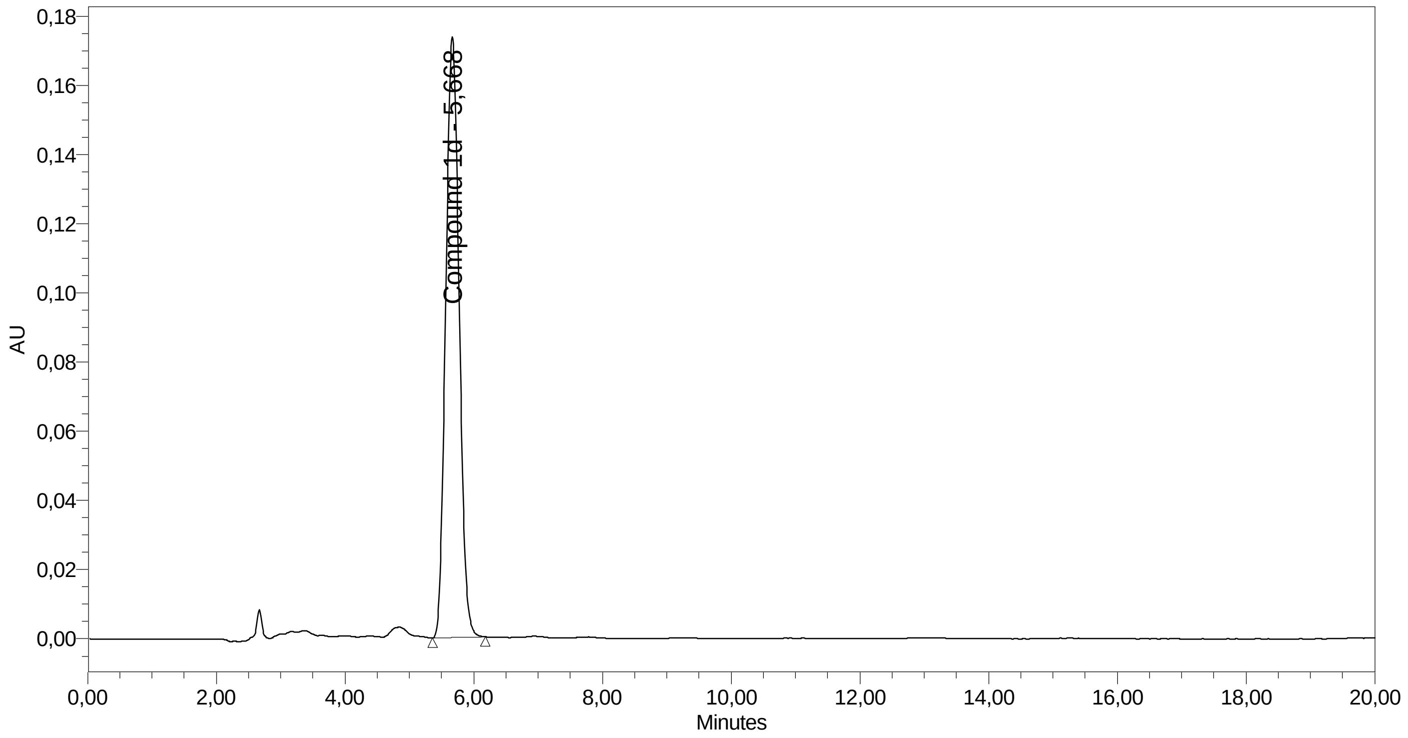

	Peak Name	RT	Area	Height (µV)	Level
1	Compound 1d	5,668	2510959	173564	3000

Seria Metoxi_1d_Linearity

Reported by User: Roxana Roman (Roxana_Roman)
Acquisition Server Waters7
Project Name: Test
Sample Set Name: Precision 1_Accuracy_PyridineM
Code column: Inertsil ODS-3, 4,6*250 mm, 5 um

SAMPLE INFORMATION

Sample Name:	Methyl-pyridine 40 ug/ mL		
Sample Type:	Standard	Date Acquired:	12.06.2022 06:15:06
Vial:	38	Acq. Method Set:	Precision 1_Accuracy_PyridineM
Injection #:	1	Date Processed:	20.02.2023 11:03:38
Injection Volume:	100,00 ul	Processing Method:	Linearity_Pyridine_M_Methoxy
Run Time:	20,0 Minutes	Channel Name:	Extract 275,0
Acquired By:	Roxana_Roman	Proc. Chnl. Descr.:	PDA 275,0 nm, Smoothed by 25

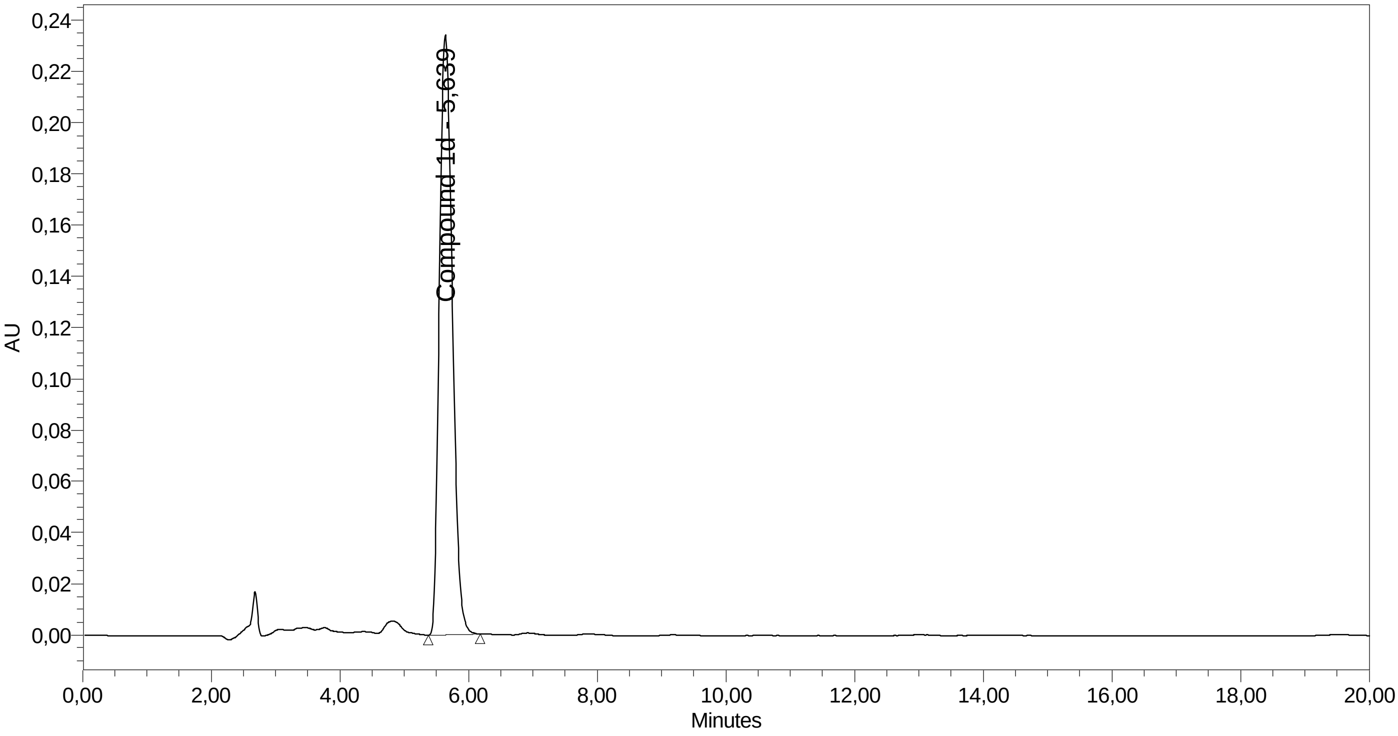

	Peak Name	RT	Area	Height (µV)	Level
1	Compound 1d	5,639	3274981	234313	4000

Seria Metoxi_1d_Linearity

Reported by User: Roxana Roman (Roxana_Roman)
Acquisition Server Waters7
Project Name: Test
Sample Set Name: Precision 1_Accuracy_PyridineM
Code column: Inertsil ODS-3, 4,6*250 mm, 5 um

S A M P L E   I N F O R M A T I O N

Sample Name:	Methyl-pyridine 40 ug/ mL		
Sample Type:	Standard	Date Acquired:	12.06.2022 06:36:09
Vial:	39	Acq. Method Set:	Precision 1_Accuracy_PyridineM
Injection #:	1	Date Processed:	20.02.2023 11:03:52
Injection Volume:	100,00 ul	Processing Method:	Linearity_Pyridine_M_Methoxy
Run Time:	20,0 Minutes	Channel Name:	Extract 275,0
Acquired By:	Roxana_Roman	Proc. Chnl. Descr.:	PDA 275,0 nm, Smoothed by 25

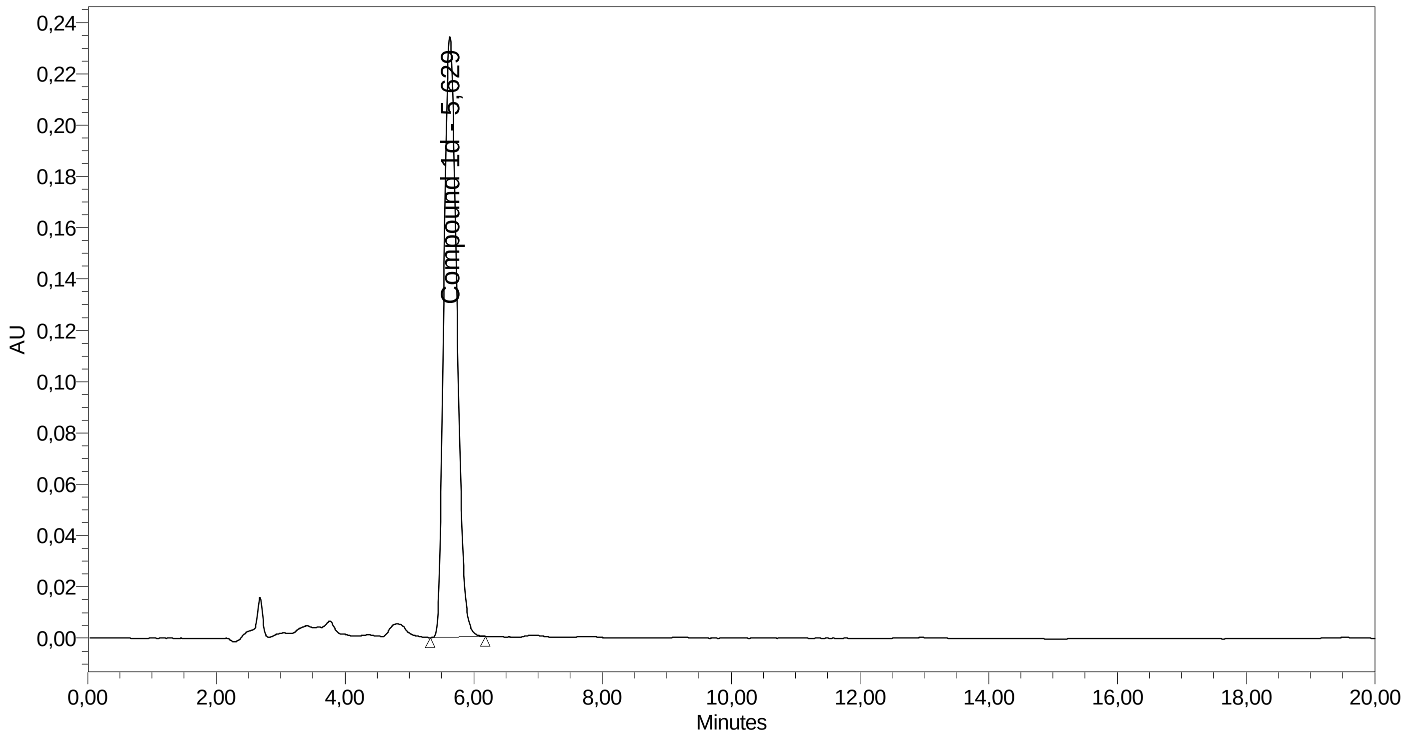

	Peak Name	RT	Area	Height (µV)	Level
1	Compound 1d	5,629	3276413	234016	4000

Quantitative determination of

compound 1d

- Validation of the analytical method -

Validation parameters:

- Specificity
- **Precision 1**
- Precision 2
- LOD – LOQ
- Linearity
- Range
- **Accuracy**

Precision 1_Accuracy_1d_Report

Reported by User: Roxana Roman (Roxana_Roman)
Acquisition Server Waters7
Project Name: Test
Sample Set Name: Precision 1_Accuracy_PyridineM
Code column: Inertsil ODS-3, 4,6*250 mm, 5 um

S A M P L E   I N F O R M A T I O N

Sample Name:	Methyl-pyridine 30 ug/ mL		
Sample Type:	Standard	Date Acquired:	11.06.2022 18:49:07
Vial:	25	Acq. Method Set:	Precision 1_Accuracy_PyridineM
Injection #:	1	Date Processed:	16.02.2023 13:11:06
Injection Volume:	100,00 ul	Processing Method:	Precision 1_Accuracy_PyridineM
Run Time:	20,0 Minutes	Channel Name:	Extract 275,0
Acquired By:	Roxana_Roman	Proc. Chnl. Descr.:	PDA 275,0 nm, Smoothed by 25

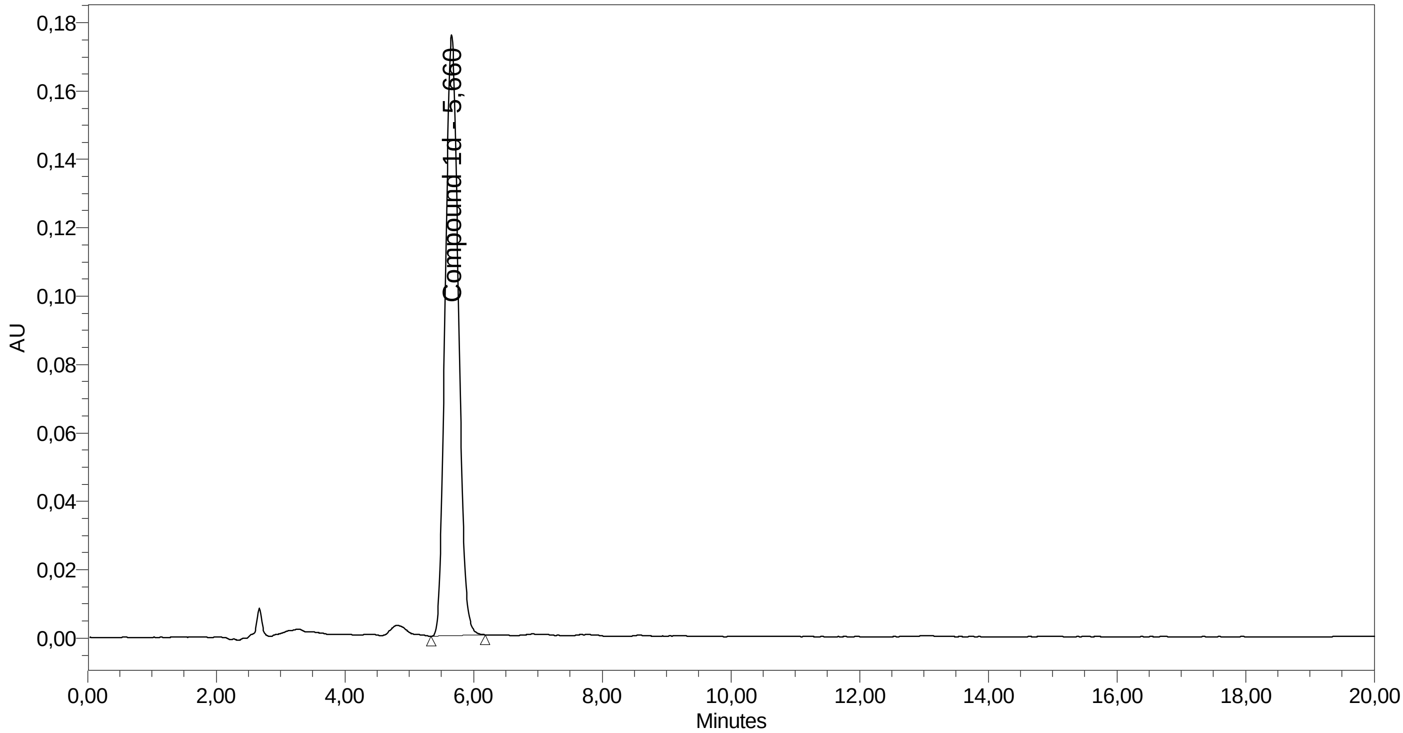

	Peak Name	RT	Area	Height (µV)	Purity1 Angle	Purity1 Threshold	Purity1 Flag
1	Compound 1d	5,660	2504606	175677	0,082	0,279	No

Precision 1_Accuracy_1d_Report

Reported by User: Roxana Roman (Roxana_Roman)
Acquisition Server Waters7
Project Name: Test
Sample Set Name: Precision 1_Accuracy_PyridineM
Code column: Inertsil ODS-3, 4,6*250 mm, 5 um

SAMPLE INFORMATION

Sample Name:	Methyl-pyridine 30 ug/ mL		
Sample Type:	Standard	Date Acquired:	11.06.2022 19:09:51
Vial:	25	Acq. Method Set:	Precision 1_Accuracy_PyridineM
Injection #:	2	Date Processed:	16.02.2023 13:11:08
Injection Volume:	100,00 ul	Processing Method:	Precision 1_Accuracy_PyridineM
Run Time:	20,0 Minutes	Channel Name:	Extract 275,0
Acquired By:	Roxana_Roman	Proc. Chnl. Descr.:	PDA 275,0 nm, Smoothed by 25

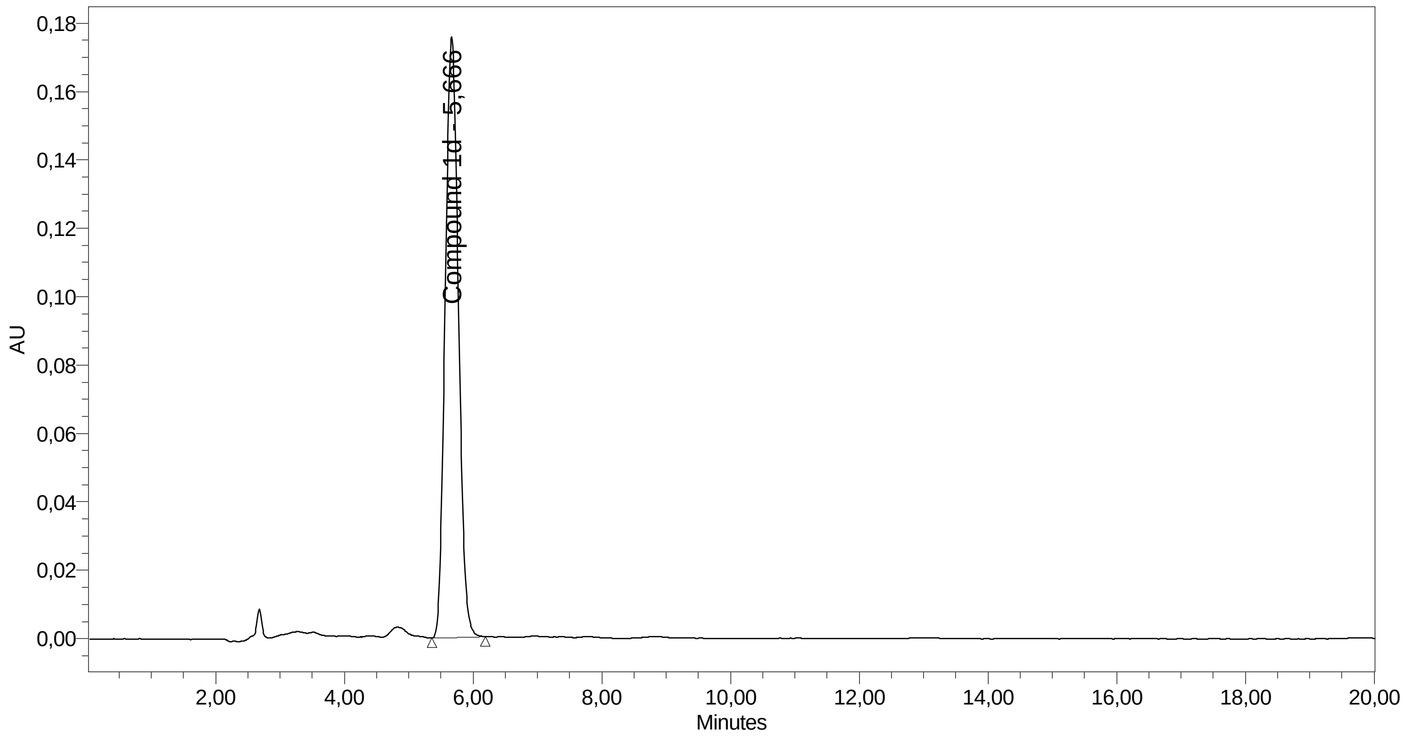

	Peak Name	RT	Area	Height (µV)	Purity1 Angle	Purity1 Threshold	Purity1 Flag
1	Compound 1d	5,666	2511937	175638	0,077	0,278	No

Precision 1_Accuracy_1d_Report

Reported by User: Roxana Roman (Roxana_Roman)
Acquisition Server Waters7
Project Name: Test
Sample Set Name: Precision 1_Accuracy_PyridineM
Code column: Inertsil ODS-3, 4,6*250 mm, 5 um

SAMPLE INFORMATION

Sample Name:	Methyl-pyridine 30 ug/ mL		
Sample Type:	Standard	Date Acquired:	11.06.2022 19:30:35
Vial:	25	Acq. Method Set:	Precision 1_Accuracy_PyridineM
Injection #:	3	Date Processed:	16.02.2023 13:11:09
Injection Volume:	100,00 ul	Processing Method:	Precision 1_Accuracy_PyridineM
Run Time:	20,0 Minutes	Channel Name:	Extract 275,0
Acquired By:	Roxana_Roman	Proc. Chnl. Descr.:	PDA 275,0 nm, Smoothed by 25

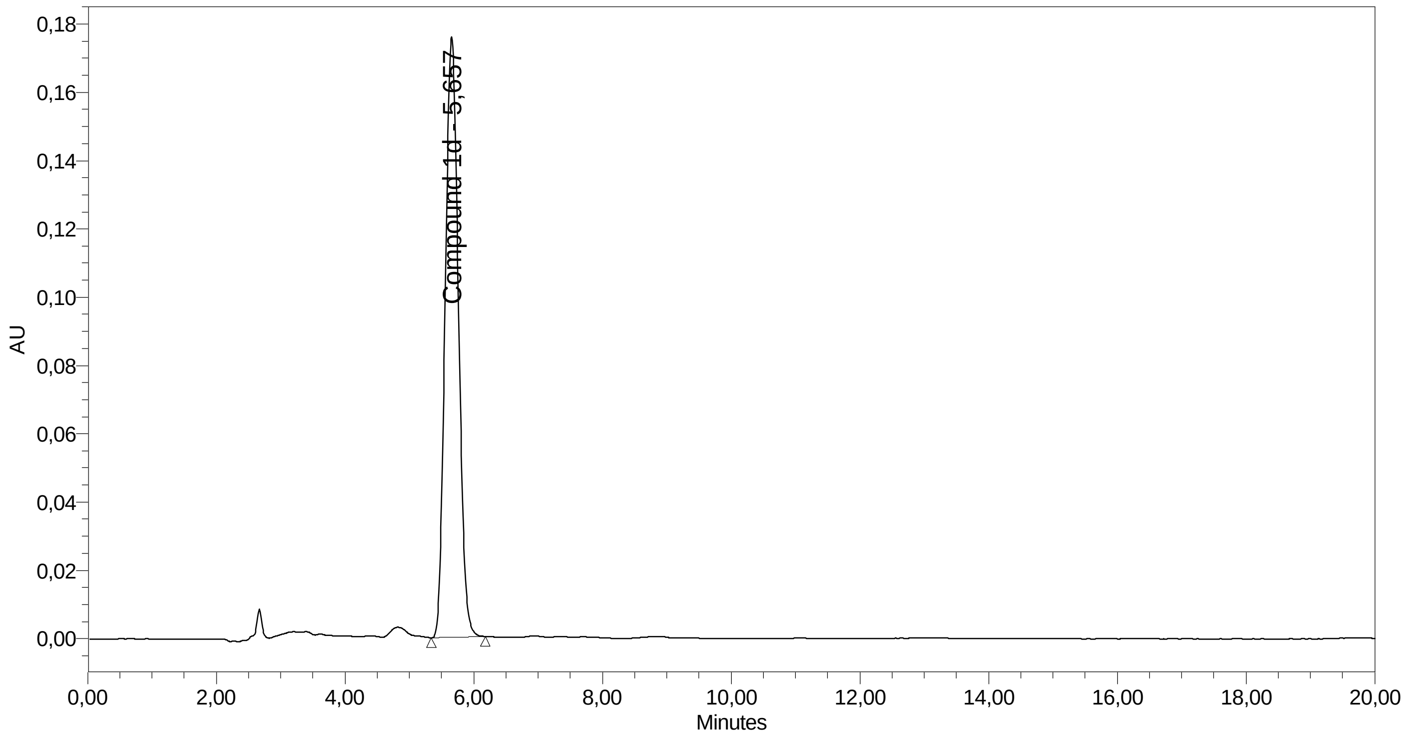

	Peak Name	RT	Area	Height (µV)	Purity1 Angle	Purity1 Threshold	Purity1 Flag
1	Compound 1d	5,657	2512040	175781	0,085	0,280	No

Component Summary Area Time

Reported by User: Roxana Roman (Roxana_Roman)
Acquisition Server Waters7
Project Name: Test
Sample Set Name: Precision 1_Accuracy_PyridineM
Code column: Inertsil ODS-3, 4,6*250 mm, 5 um

Component Summary For Retention Time
Channel: W2996

	SampleName	Inj	Channel	Vial	Compound 1d
1	Methyl-pyridine 30 ug/ mL	1	W2996	25	5,660
2	Methyl-pyridine 30 ug/ mL	2	W2996	25	5,666
3	Methyl-pyridine 30 ug/ mL	3	W2996	25	5,657
Mean					5,661
Std. Dev.					0,004
% RSD					0,08

Component Summary For Area
Channel: W2996

	SampleName	Inj	Channel	Vial	Compound 1d
1	Methyl-pyridine 30 ug/ mL	1	W2996	25	2504606
2	Methyl-pyridine 30 ug/ mL	2	W2996	25	2511937
3	Methyl-pyridine 30 ug/ mL	3	W2996	25	2512040
Mean					2509528
Std. Dev.					4263
% RSD					0,17

Precision 1_Accuracy_1d_Report

Reported by User: Roxana Roman (Roxana_Roman)
Acquisition Server Waters7
Project Name: Test
Sample Set Name: Precision 1_Accuracy_PyridineM
Code column: Inertsil ODS-3, 4,6*250 mm, 5 um

SAMPLE INFORMATION

Sample Name:	Methanol		
Sample Type:	Standard	Date Acquired:	11.06.2022 18:28:06
Vial:	11	Acq. Method Set:	Precision 1_Accuracy_PyridineM
Injection #:	1	Date Processed:	18.02.2023 09:25:05
Injection Volume:	100,00 ul	Processing Method:	Precision 1_Accuracy_PyridineM
Run Time:	20,0 Minutes	Channel Name:	Extract 275,0
Acquired By:	Roxana_Roman	Proc. Chnl. Descr.:	PDA 275,0 nm, Smoothed by 25

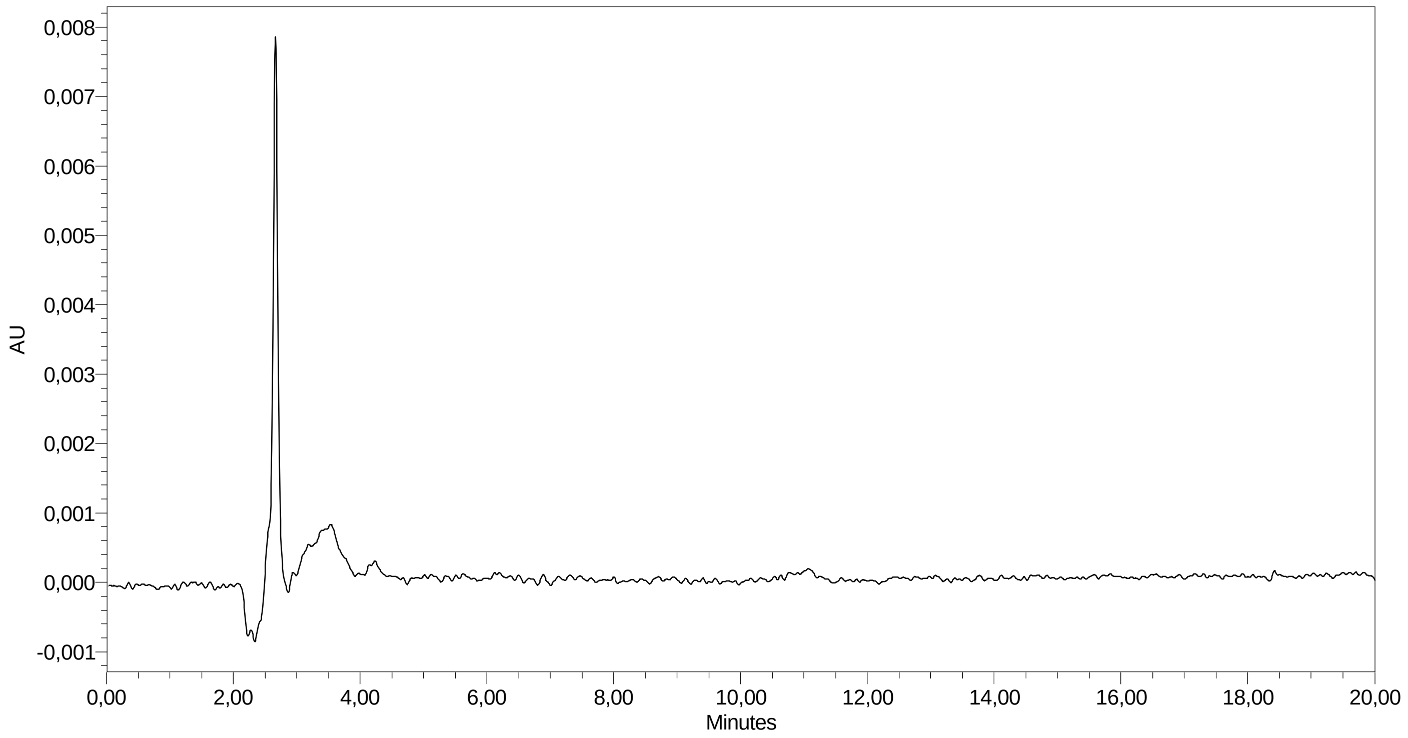

Basic LC Peaks Table group contains no data.

Precision 1_Accuracy_1d_Report

Reported by User: Roxana Roman (Roxana_Roman)
Acquisition Server Waters7
Project Name: Test
Sample Set Name: Precision 1_Accuracy_PyridineM
Code column: Inertsil ODS-3, 4,6*250 mm, 5 um

SAMPLE INFORMATION

Sample Name:	Methyl-pyridine 20 ug/ mL		
Sample Type:	Unknown	Date Acquired:	11.06.2022 20:16:34
Vial:	26	Acq. Method Set:	Precision 1_Accuracy_PyridineM
Injection #:	1	Date Processed:	16.02.2023 13:11:12
Injection Volume:	100,00 ul	Processing Method:	Precision 1_Accuracy_PyridineM
Run Time:	20,0 Minutes	Channel Name:	Extract 275,0
Acquired By:	Roxana_Roman	Proc. Chnl. Descr.:	PDA 275,0 nm, Smoothed by 25

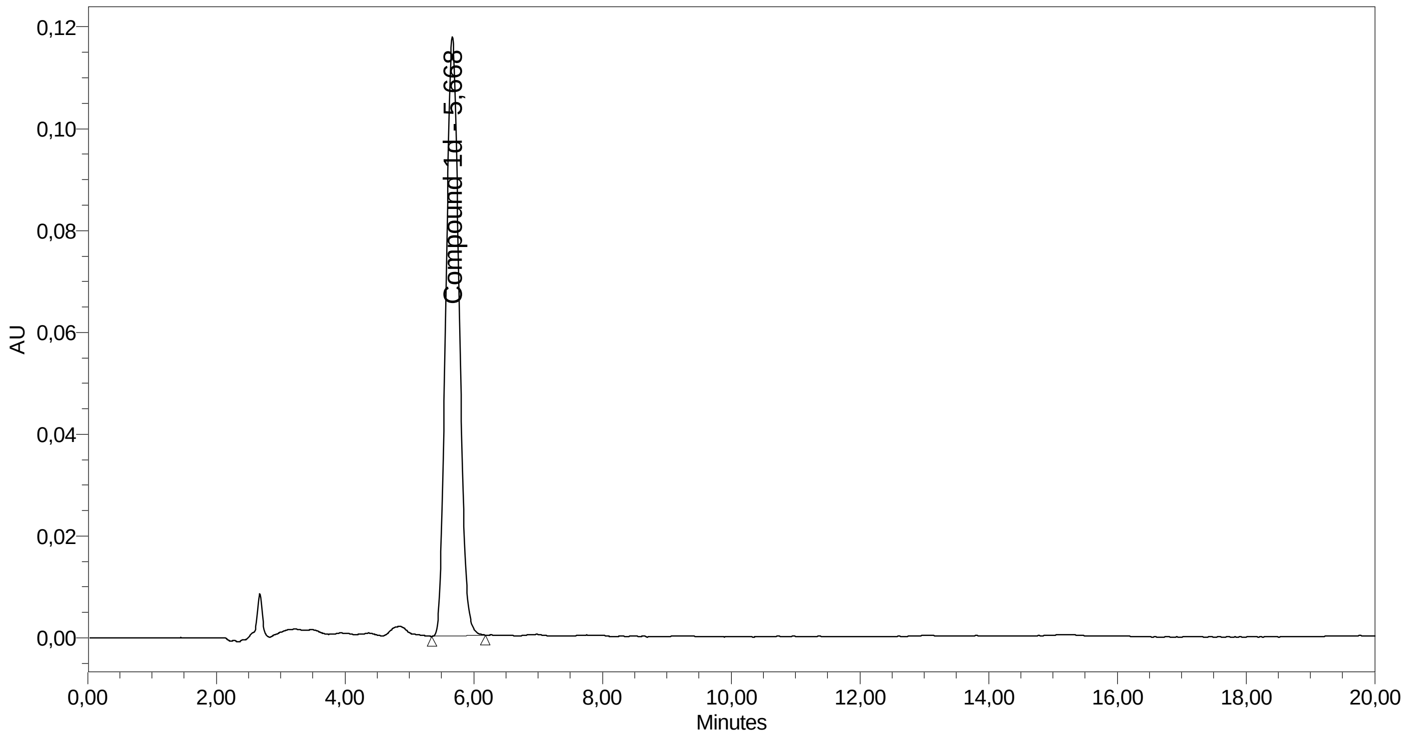

	Peak Name	RT	Area	Height (µV)	Purity1 Angle	Purity1 Threshold	Purity1 Flag
1	Compound 1d	5,668	1683470	117640	0,107	0,281	No

Precision 1_Accuracy_1d_Report

Reported by User: Roxana Roman (Roxana_Roman)
Acquisition Server Waters7
Project Name: Test
Sample Set Name: Precision 1_Accuracy_PyridineM
Code column: Inertsil ODS-3, 4,6*250 mm, 5 um

SAMPLE INFORMATION

Sample Name:	Methyl-pyridine 20 ug/ mL		
Sample Type:	Unknown	Date Acquired:	11.06.2022 20:37:21
Vial:	27	Acq. Method Set:	Precision 1_Accuracy_PyridineM
Injection #:	1	Date Processed:	16.02.2023 13:11:13
Injection Volume:	100,00 ul	Processing Method:	Precision 1_Accuracy_PyridineM
Run Time:	20,0 Minutes	Channel Name:	Extract 275,0
Acquired By:	Roxana_Roman	Proc. Chnl. Descr.:	PDA 275,0 nm, Smoothed by 25

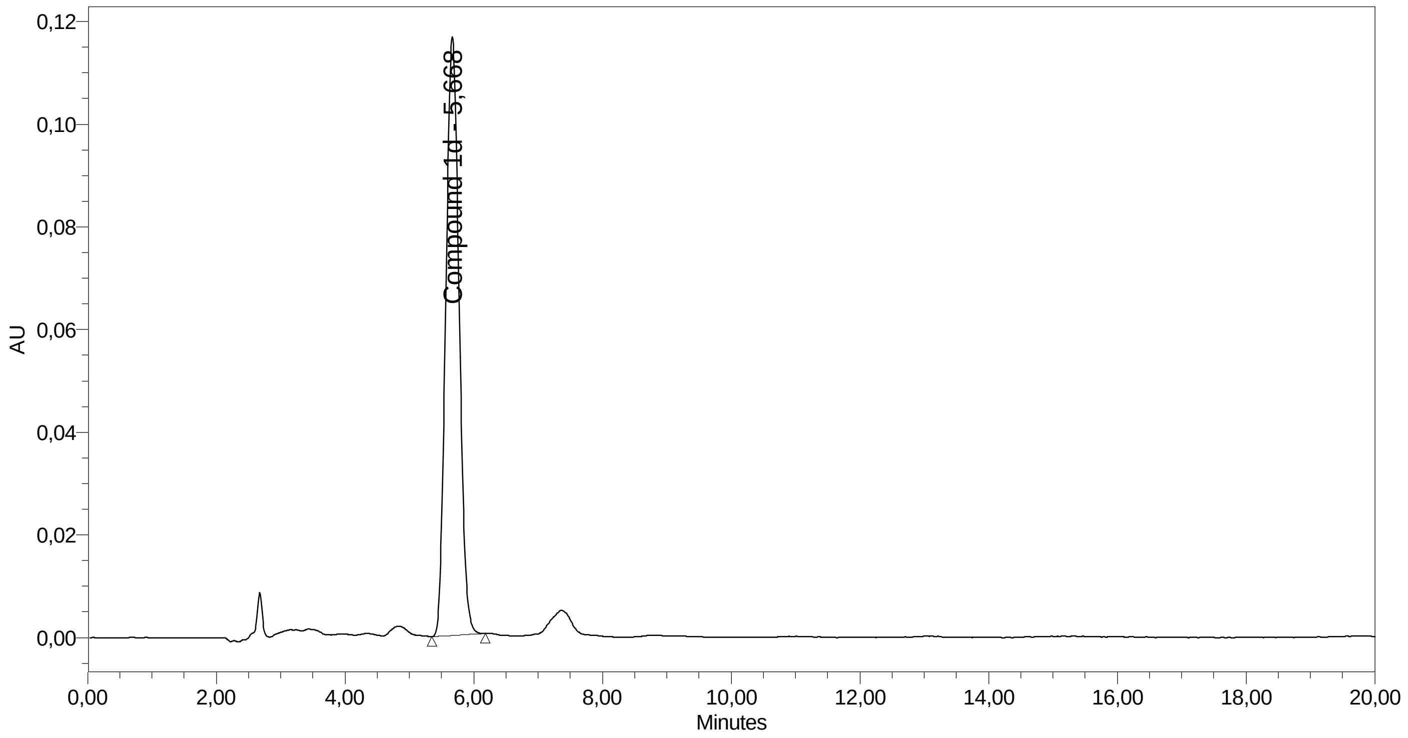

	Peak Name	RT	Area	Height (µV)	Purity1 Angle	Purity1 Threshold	Purity1 Flag
1	Compound 1d	5,668	1671584	116577	0,158	0,289	No

Precision 1_Accuracy_1d_Report

Reported by User: Roxana Roman (Roxana_Roman)
Acquisition Server Waters7
Project Name: Test
Sample Set Name: Precision 1_Accuracy_PyridineM
Code column: Inertsil ODS-3, 4,6*250 mm, 5 um

SAMPLE INFORMATION

Sample Name:	Methyl-pyridine 20 ug/ mL		
Sample Type:	Unknown	Date Acquired:	11.06.2022 20:58:09
Vial:	28	Acq. Method Set:	Precision 1_Accuracy_PyridineM
Injection #:	1	Date Processed:	16.02.2023 13:12:01
Injection Volume:	100,00 ul	Processing Method:	Precision 1_Accuracy_PyridineM
Run Time:	20,0 Minutes	Channel Name:	Extract 275,0
Acquired By:	Roxana_Roman	Proc. Chnl. Descr.:	PDA 275,0 nm, Smoothed by 25

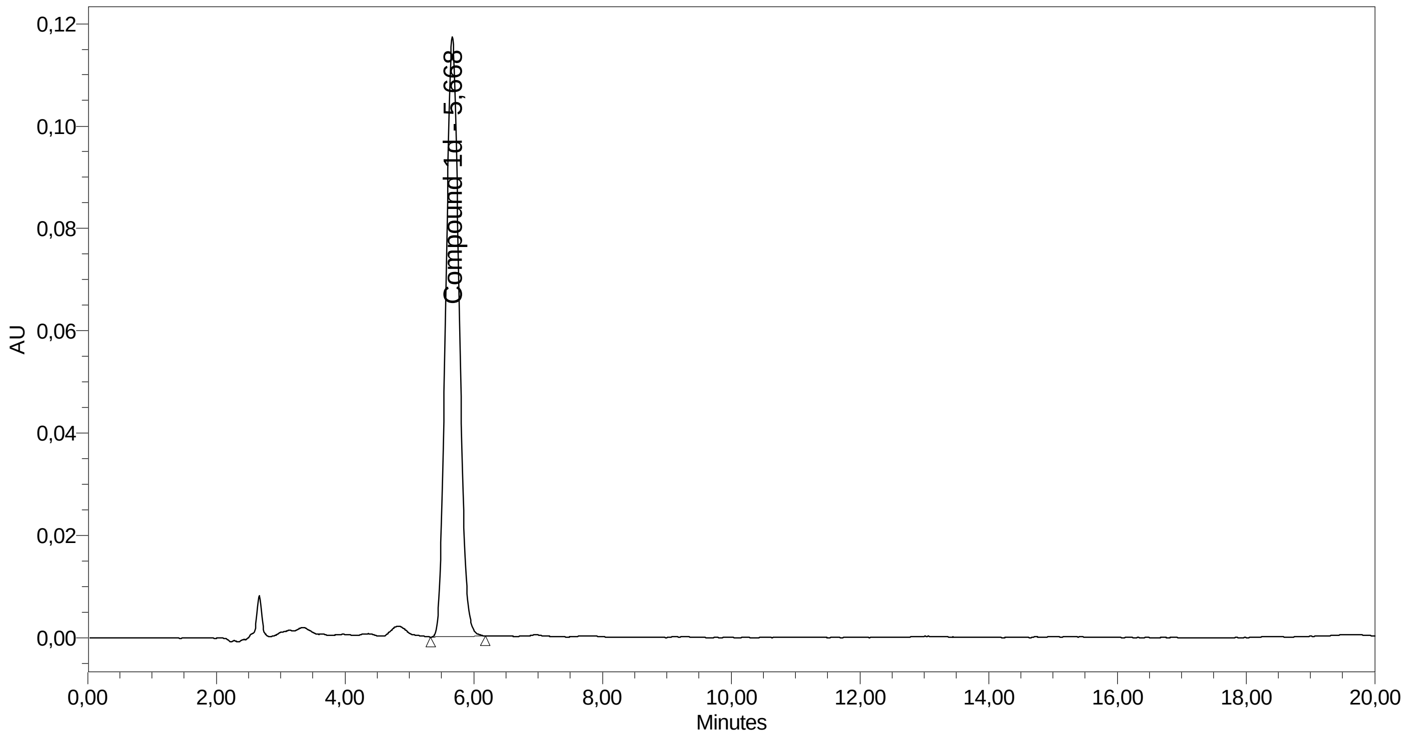

	Peak Name	RT	Area	Height (µV)	Purity1 Angle	Purity1 Threshold	Purity1 Flag
1	Compound 1d	5,668	1692685	117186	0,111	0,278	No

Precision 1_Accuracy_1d_Report

Reported by User: Roxana Roman (Roxana_Roman)
Acquisition Server Waters7
Project Name: Test
Sample Set Name: Precision 1_Accuracy_PyridineM
Code column: Inertsil ODS-3, 4,6*250 mm, 5 um

S A M P L E   I N F O R M A T I O N

Sample Name:	Methyl-pyridine 20 ug/ mL		
Sample Type:	Unknown	Date Acquired:	11.06.2022 21:18:57
Vial:	29	Acq. Method Set:	Precision 1_Accuracy_PyridineM
Injection #:	1	Date Processed:	16.02.2023 13:12:15
Injection Volume:	100,00 ul	Processing Method:	Precision 1_Accuracy_PyridineM
Run Time:	20,0 Minutes	Channel Name:	Extract 275,0
Acquired By:	Roxana_Roman	Proc. Chnl. Descr.:	PDA 275,0 nm, Smoothed by 25

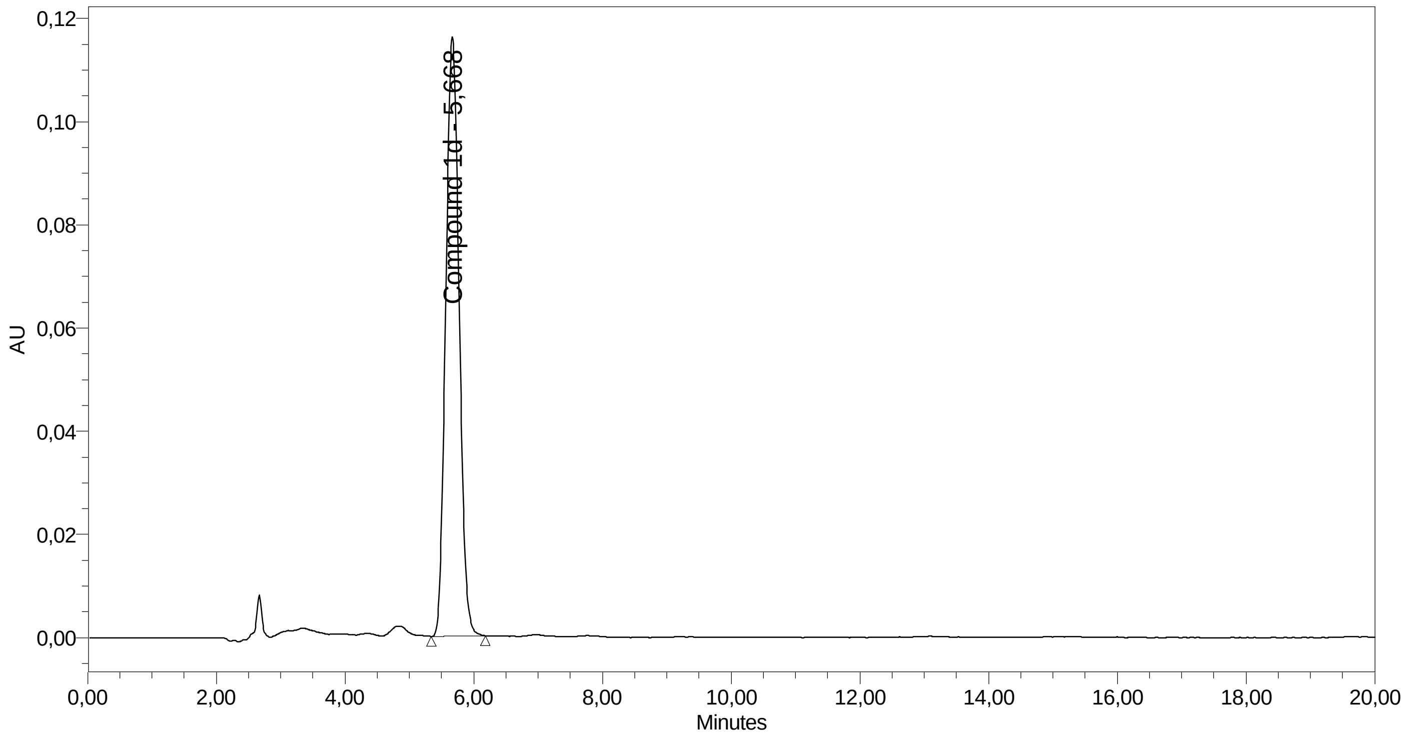

	Peak Name	RT	Area	Height (µV)	Purity1 Angle	Purity1 Threshold	Purity1 Flag
1	Compound 1d	5,668	1676085	116109	0,114	0,285	No

Precision 1_Accuracy_1d_Report

Reported by User: Roxana Roman (Roxana_Roman)
Acquisition Server Waters7
Project Name: Test
Sample Set Name: Precision 1_Accuracy_PyridineM
Code column: Inertsil ODS-3, 4,6*250 mm, 5 um

SAMPLE INFORMATION

Sample Name:	Methyl-pyridine 20 ug/ mL		
Sample Type:	Unknown	Date Acquired:	11.06.2022 21:39:46
Vial:	30	Acq. Method Set:	Precision 1_Accuracy_PyridineM
Injection #:	1	Date Processed:	16.02.2023 13:12:25
Injection Volume:	100,00 ul	Processing Method:	Precision 1_Accuracy_PyridineM
Run Time:	20,0 Minutes	Channel Name:	Extract 275,0
Acquired By:	Roxana_Roman	Proc. Chnl. Descr.:	PDA 275,0 nm, Smoothed by 25

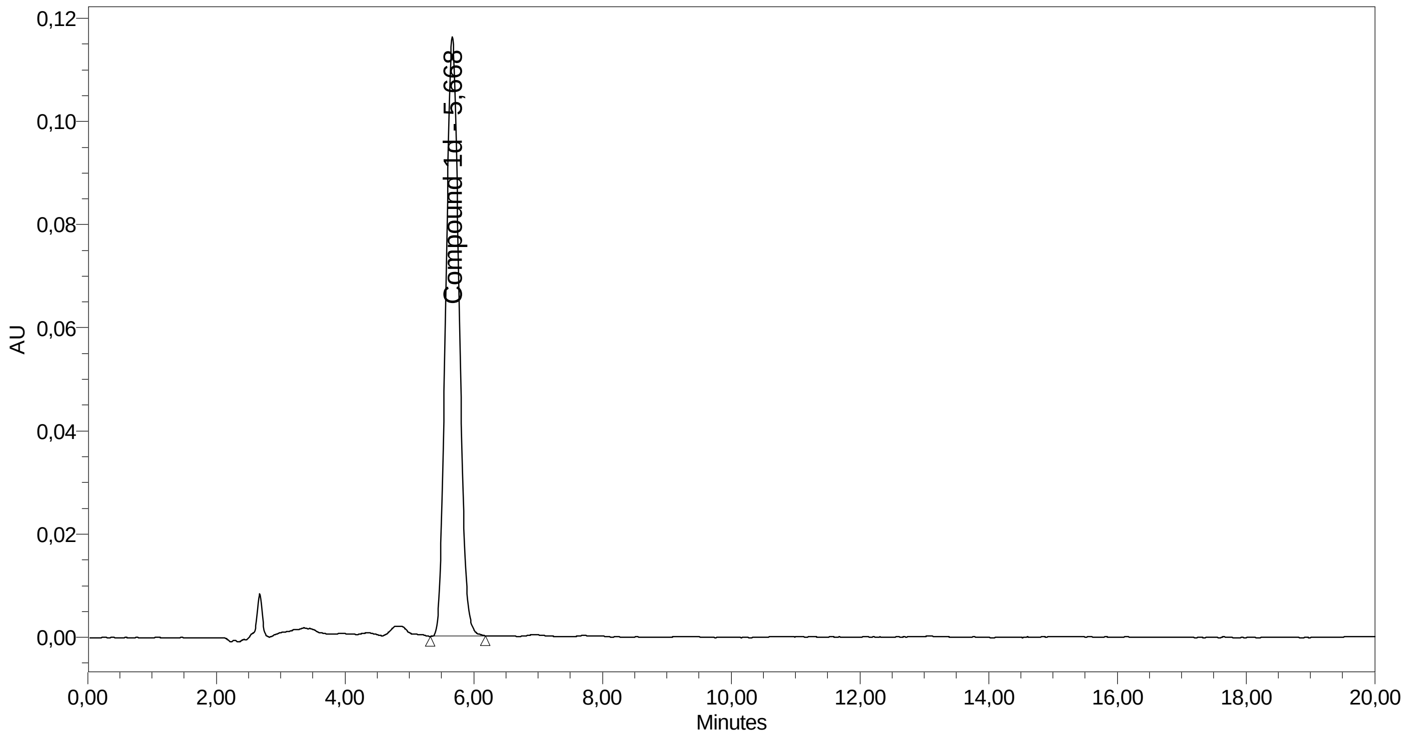

	Peak Name	RT	Area	Height (µV)	Purity1 Angle	Purity1 Threshold	Purity1 Flag
1	Compound 1d	5,668	1674530	116125	0,126	0,285	No

Precision 1_Accuracy_1d_Report

Reported by User: Roxana Roman (Roxana_Roman)
Acquisition Server Waters7
Project Name: Test
Sample Set Name: Precision 1_Accuracy_PyridineM
Code column: Inertsil ODS-3, 4,6*250 mm, 5 um

SAMPLE INFORMATION

Sample Name:	Methyl-pyridine 20 ug/ mL		
Sample Type:	Unknown	Date Acquired:	11.06.2022 22:00:33
Vial:	31	Acq. Method Set:	Precision 1_Accuracy_PyridineM
Injection #:	1	Date Processed:	16.02.2023 13:12:35
Injection Volume:	100,00 ul	Processing Method:	Precision 1_Accuracy_PyridineM
Run Time:	20,0 Minutes	Channel Name:	Extract 275,0
Acquired By:	Roxana_Roman	Proc. Chnl. Descr.:	PDA 275,0 nm, Smoothed by 25

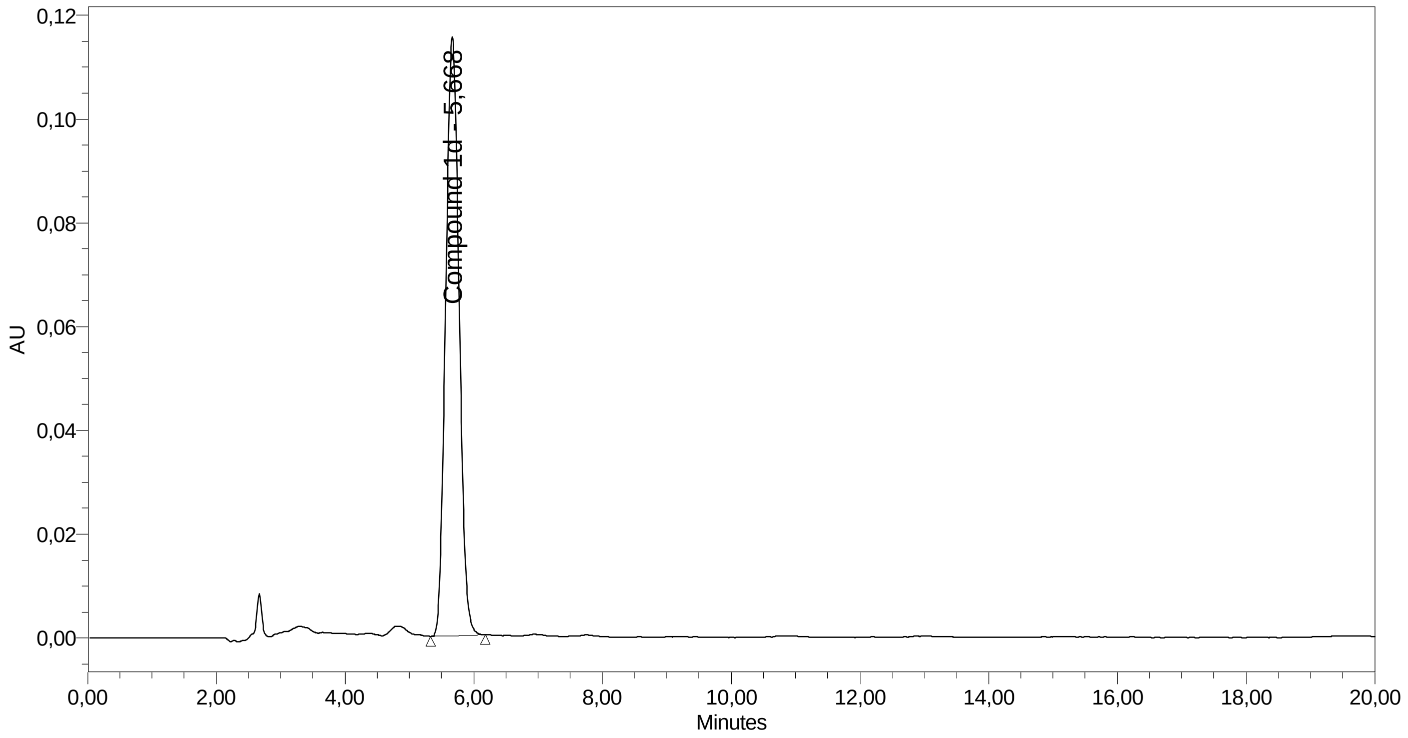

	Peak Name	RT	Area	Height (µV)	Purity1 Angle	Purity1 Threshold	Purity1 Flag
1	Compound 1d	5,668	1675112	115410	0,117	0,281	No

Precision 1_Accuracy_1d_Report

Reported by User: Roxana Roman (Roxana_Roman)
Acquisition Server Waters7
Project Name: Test
Sample Set Name: Precision 1_Accuracy_PyridineM
Code column: Inertsil ODS-3, 4,6*250 mm, 5 um

SAMPLE INFORMATION

Sample Name:	Methyl-pyridine 30 ug/ mL		
Sample Type:	Unknown	Date Acquired:	11.06.2022 22:21:21
Vial:	32	Acq. Method Set:	Precision 1_Accuracy_PyridineM
Injection #:	1	Date Processed:	16.02.2023 13:12:52
Injection Volume:	100,00 ul	Processing Method:	Precision 1_Accuracy_PyridineM
Run Time:	20,0 Minutes	Channel Name:	Extract 275,0
Acquired By:	Roxana_Roman	Proc. Chnl. Descr.:	PDA 275,0 nm, Smoothed by 25

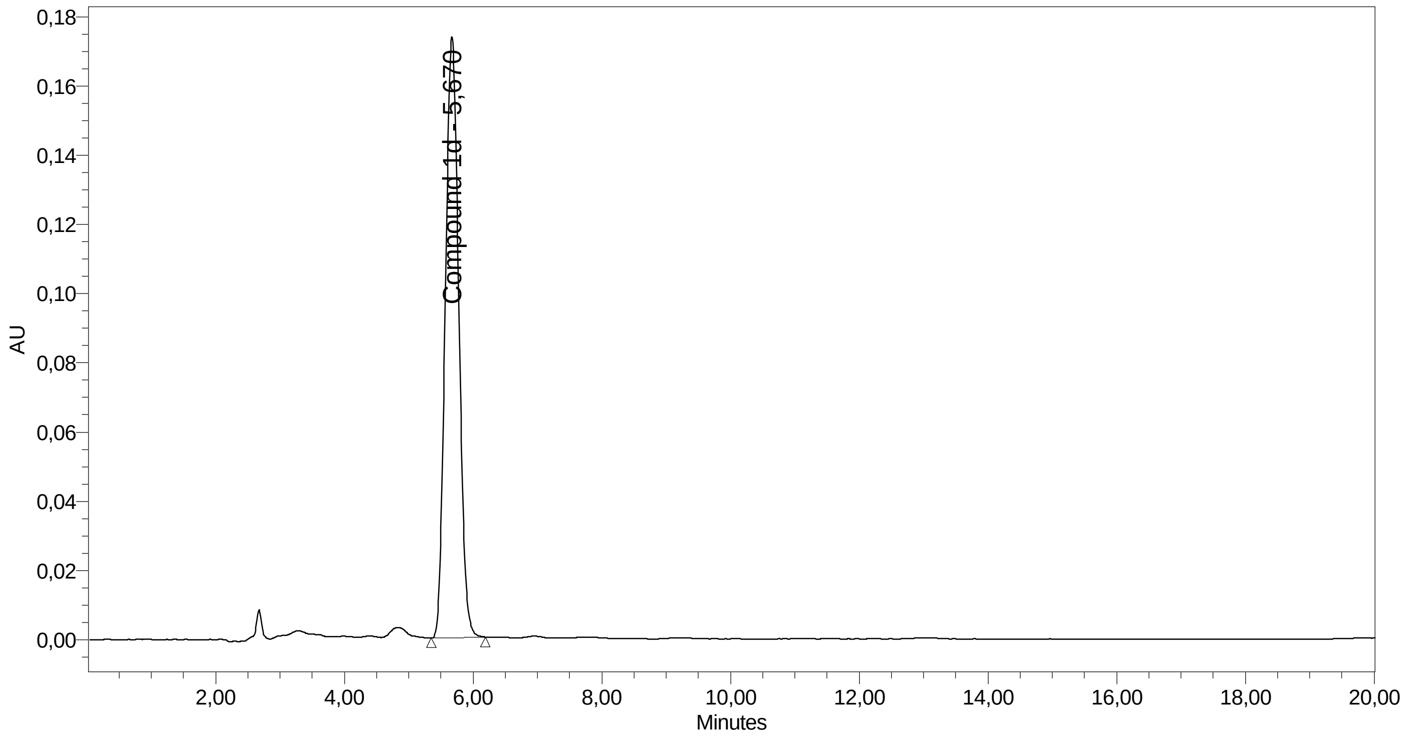

	Peak Name	RT	Area	Height (µV)	Purity1 Angle	Purity1 Threshold	Purity1 Flag
1	Compound 1d	5,670	2518986	173649	0,081	0,279	No

Precision 1_Accuracy_1d_Report

Reported by User: Roxana Roman (Roxana_Roman)
Acquisition Server Waters7
Project Name: Test
Sample Set Name: Precision 1_Accuracy_PyridineM
Code column: Inertsil ODS-3, 4,6*250 mm, 5 um

SAMPLE INFORMATION

Sample Name:	Methyl-pyridine 30 ug/ mL		
Sample Type:	Unknown	Date Acquired:	11.06.2022 22:42:09
Vial:	33	Acq. Method Set:	Precision 1_Accuracy_PyridineM
Injection #:	1	Date Processed:	16.02.2023 13:13:03
Injection Volume:	100,00 ul	Processing Method:	Precision 1_Accuracy_PyridineM
Run Time:	20,0 Minutes	Channel Name:	Extract 275,0
Acquired By:	Roxana_Roman	Proc. Chnl. Descr.:	PDA 275,0 nm, Smoothed by 25

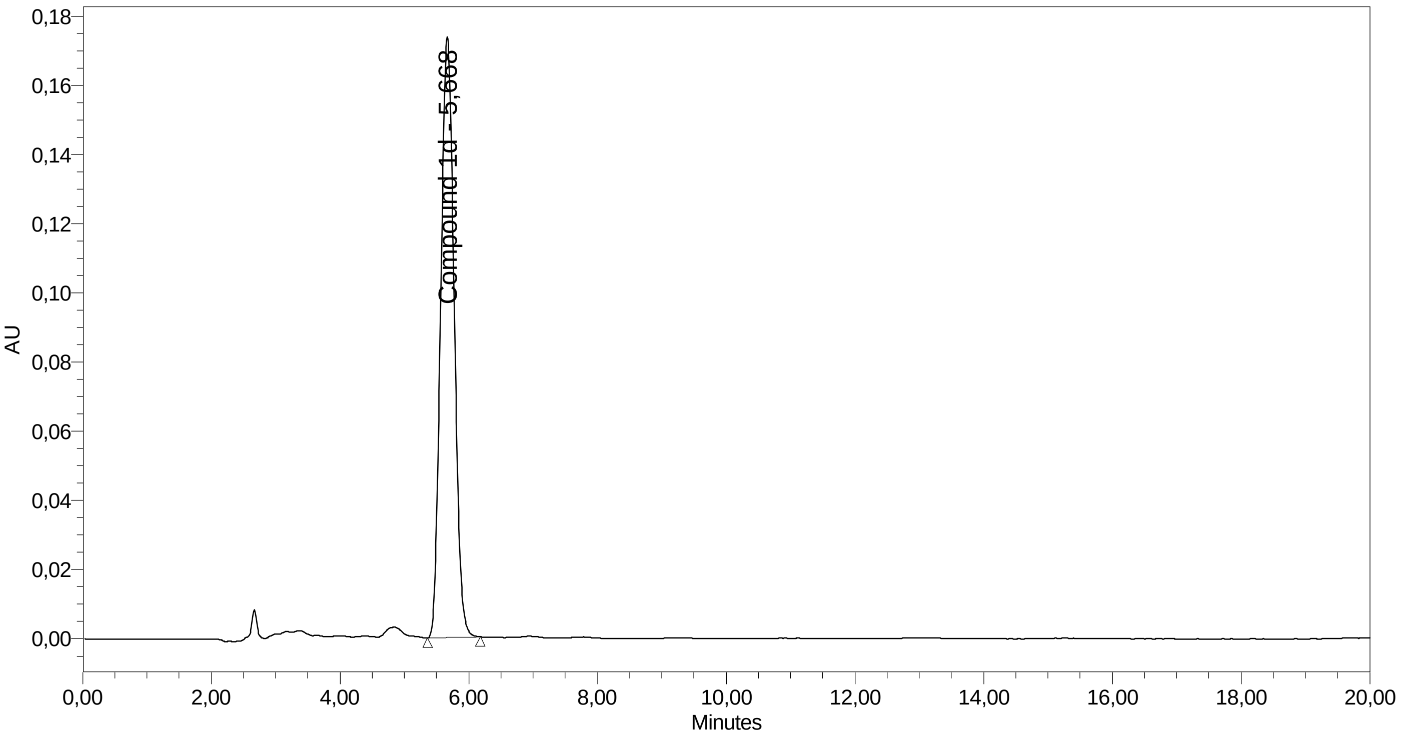

	Peak Name	RT	Area	Height (µV)	Purity1 Angle	Purity1 Threshold	Purity1 Flag
1	Compound 1d	5,668	2510959	173564	0,080	0,280	No

Precision 1_Accuracy_1d_Report

Reported by User: Roxana Roman (Roxana_Roman)
Acquisition Server Waters7
Project Name: Test
Sample Set Name: Precision 1_Accuracy_PyridineM
Code column: Inertsil ODS-3, 4,6*250 mm, 5 um

SAMPLE INFORMATION

Sample Name:	Methyl-pyridine 30 ug/ mL		
Sample Type:	Unknown	Date Acquired:	11.06.2022 23:02:57
Vial:	34	Acq. Method Set:	Precision 1_Accuracy_PyridineM
Injection #:	1	Date Processed:	16.02.2023 13:13:11
Injection Volume:	100,00 ul	Processing Method:	Precision 1_Accuracy_PyridineM
Run Time:	20,0 Minutes	Channel Name:	Extract 275,0
Acquired By:	Roxana_Roman	Proc. Chnl. Descr.:	PDA 275,0 nm, Smoothed by 25

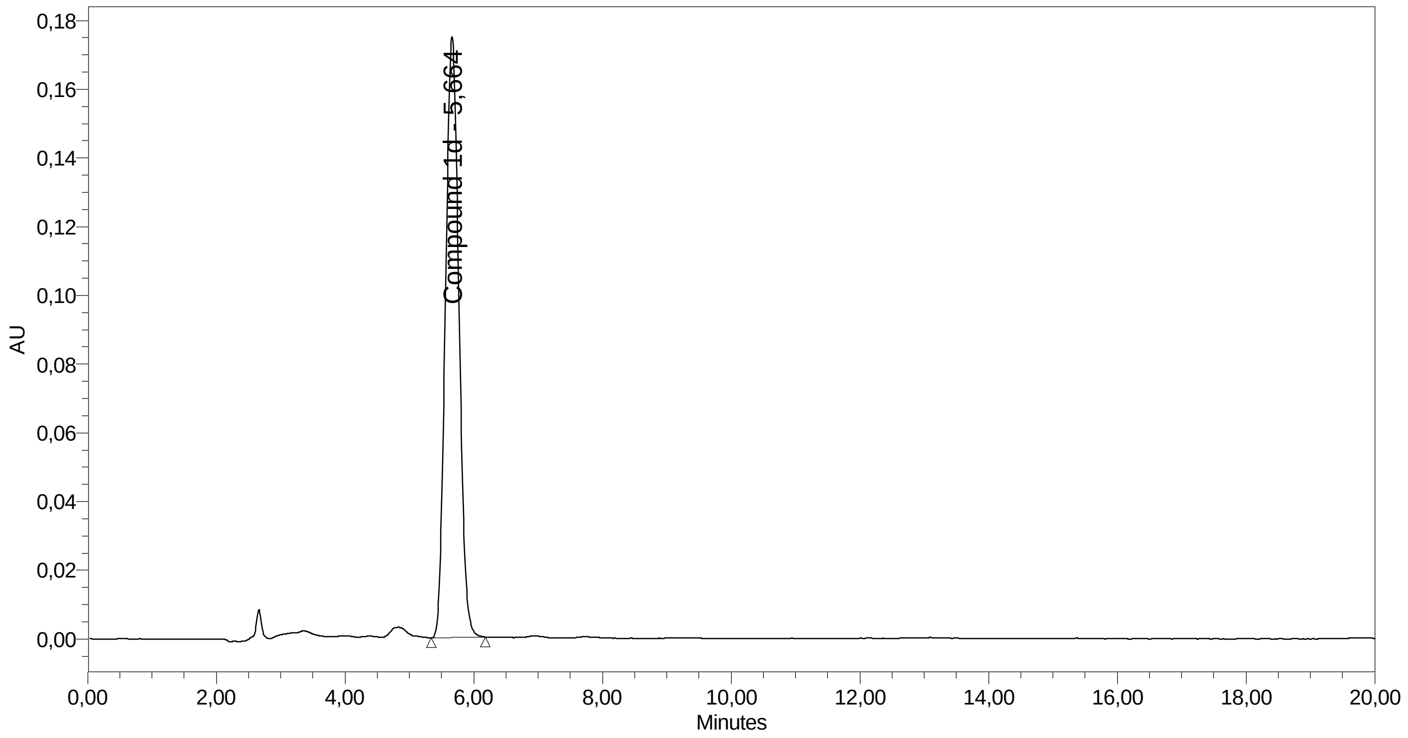

	Peak Name	RT	Area	Height (µV)	Purity1 Angle	Purity1 Threshold	Purity1 Flag
1	Compound 1d	5,664	2537030	174826	0,082	0,284	No

Precision 1_Accuracy_1d_Report

Reported by User: Roxana Roman (Roxana_Roman)
Acquisition Server Waters7
Project Name: Test
Sample Set Name: Precision 1_Accuracy_PyridineM
Code column: Inertsil ODS-3, 4,6*250 mm, 5 um

SAMPLE INFORMATION

Sample Name:	Methyl-pyridine 30 ug/ mL		
Sample Type:	Unknown	Date Acquired:	11.06.2022 23:23:45
Vial:	35	Acq. Method Set:	Precision 1_Accuracy_PyridineM
Injection #:	1	Date Processed:	16.02.2023 13:13:19
Injection Volume:	100,00 ul	Processing Method:	Precision 1_Accuracy_PyridineM
Run Time:	20,0 Minutes	Channel Name:	Extract 275,0
Acquired By:	Roxana_Roman	Proc. Chnl. Descr.:	PDA 275,0 nm, Smoothed by 25

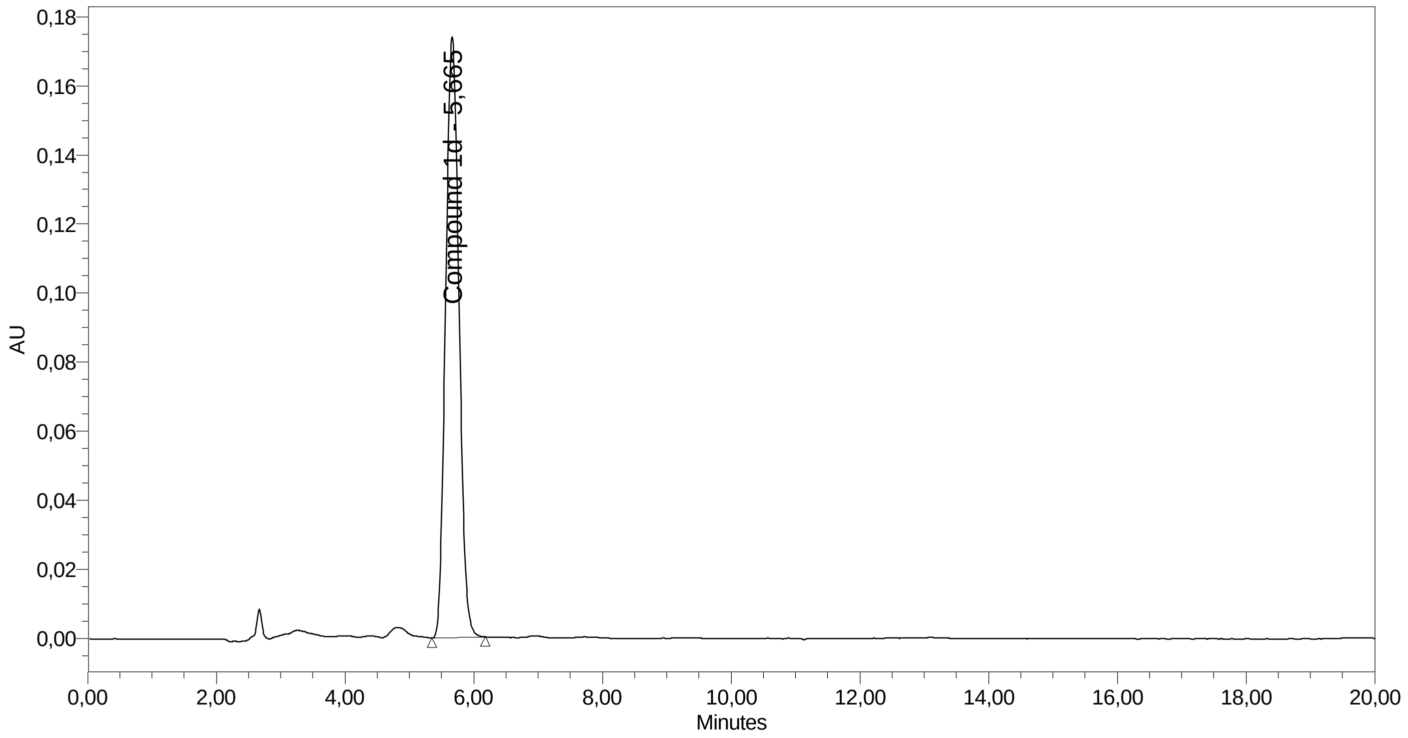

	Peak Name	RT	Area	Height (µV)	Purity1 Angle	Purity1 Threshold	Purity1 Flag
1	Compound 1d	5,665	2510454	173931	0,079	0,280	No

Precision 1_Accuracy_1d_Report

Reported by User: Roxana Roman (Roxana_Roman)
Acquisition Server Waters7
Project Name: Test
Sample Set Name: Precision 1_Accuracy_PyridineM
Code column: Inertsil ODS-3, 4,6*250 mm, 5 um

SAMPLE INFORMATION

Sample Name:	Methyl-pyridine 30 ug/ mL		
Sample Type:	Unknown	Date Acquired:	11.06.2022 23:44:33
Vial:	36	Acq. Method Set:	Precision 1_Accuracy_PyridineM
Injection #:	1	Date Processed:	16.02.2023 13:13:28
Injection Volume:	100,00 ul	Processing Method:	Precision 1_Accuracy_PyridineM
Run Time:	20,0 Minutes	Channel Name:	Extract 275,0
Acquired By:	Roxana_Roman	Proc. Chnl. Descr.:	PDA 275,0 nm, Smoothed by 25

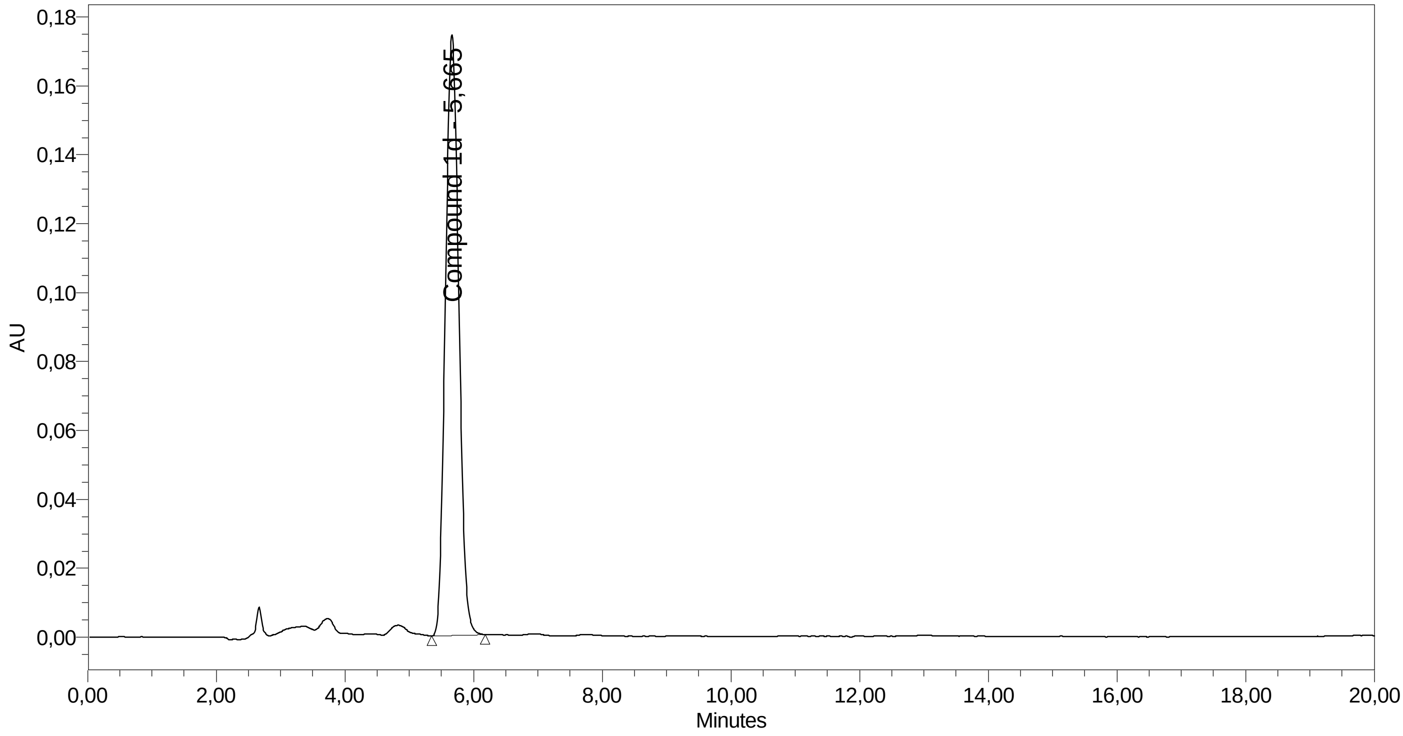

	Peak Name	RT	Area	Height (µV)	Purity1 Angle	Purity1 Threshold	Purity1 Flag
1	Compound 1d	5,665	2518396	174269	0,080	0,279	No

Precision 1_Accuracy_1d_Report

Reported by User: Roxana Roman (Roxana_Roman)
Acquisition Server Waters7
Project Name: Test
Sample Set Name: Precision 1_Accuracy_PyridineM
Code column: Inertsil ODS-3, 4,6*250 mm, 5 um

S A M P L E   I N F O R M A T I O N

Sample Name:	Methyl-pyridine 30 ug/ mL		
Sample Type:	Unknown	Date Acquired:	12.06.2022 00:05:21
Vial:	37	Acq. Method Set:	Precision 1_Accuracy_PyridineM
Injection #:	1	Date Processed:	16.02.2023 13:13:38
Injection Volume:	100,00 ul	Processing Method:	Precision 1_Accuracy_PyridineM
Run Time:	20,0 Minutes	Channel Name:	Extract 275,0
Acquired By:	Roxana_Roman	Proc. Chnl. Descr.:	PDA 275,0 nm, Smoothed by 25

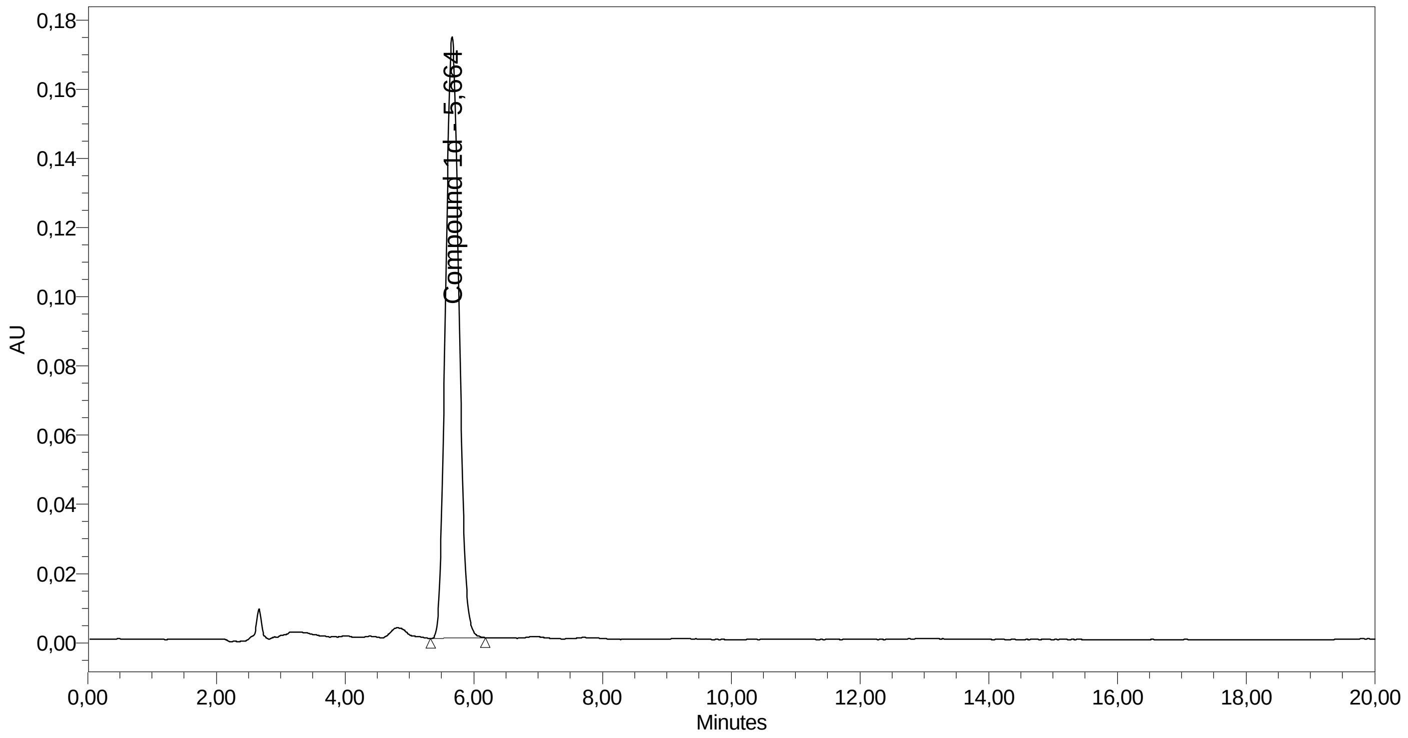

	Peak Name	RT	Area	Height (µV)	Purity1 Angle	Purity1 Threshold	Purity1 Flag
1	Compound 1d	5,664	2514887	173812	0,086	0,282	No

Precision 1_Accuracy_1d_Report

Reported by User: Roxana Roman (Roxana_Roman)
Acquisition Server Waters7
Project Name: Test
Sample Set Name: Precision 1_Accuracy_PyridineM
Code column: Inertsil ODS-3, 4,6*250 mm, 5 um

SAMPLE INFORMATION

Sample Name:	Methyl-pyridine 40 ug/ mL		
Sample Type:	Unknown	Date Acquired:	12.06.2022 06:15:06
Vial:	38	Acq. Method Set:	Precision 1_Accuracy_PyridineM
Injection #:	1	Date Processed:	18.02.2023 09:26:31
Injection Volume:	100,00 ul	Processing Method:	Precision 1_Accuracy_PyridineM
Run Time:	20,0 Minutes	Channel Name:	Extract 275,0
Acquired By:	Roxana_Roman	Proc. Chnl. Descr.:	PDA 275,0 nm, Smoothed by 25

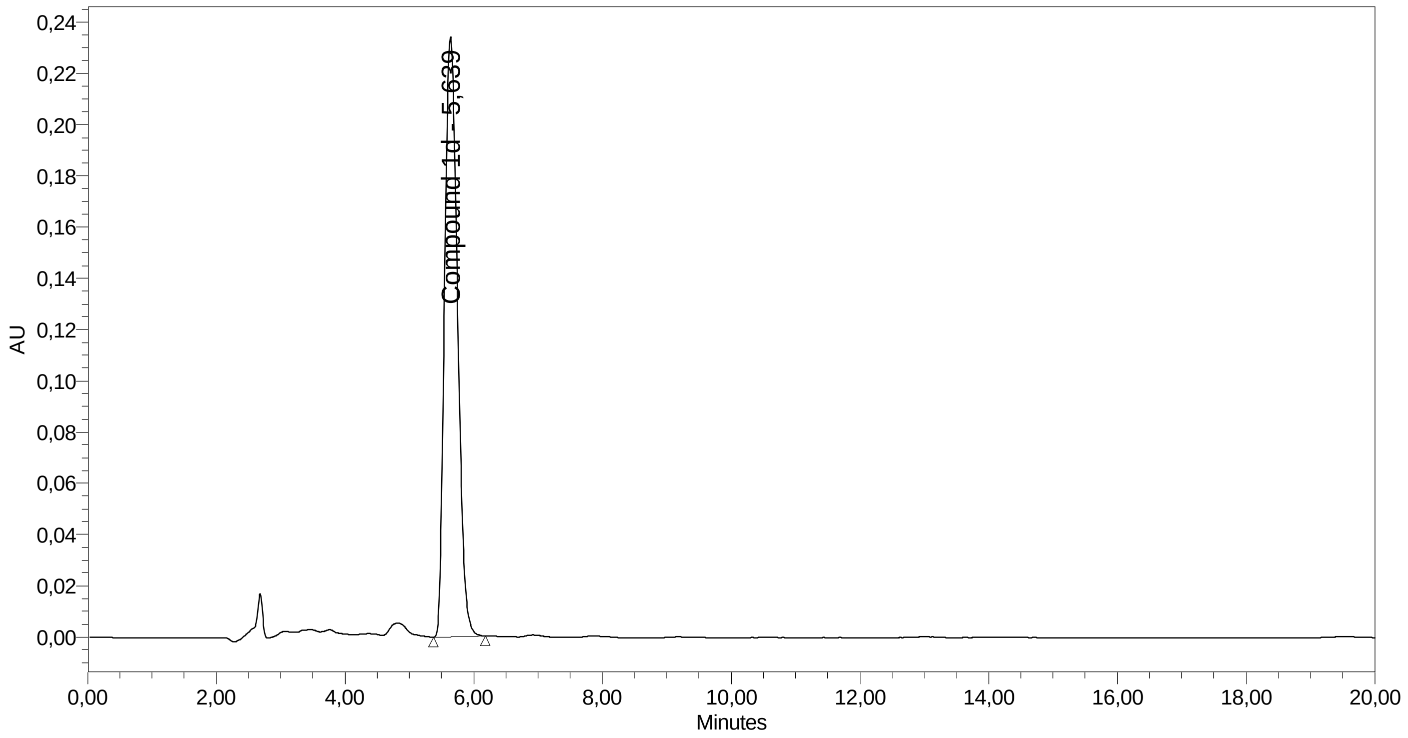

	Peak Name	RT	Area	Height (µV)	Purity1 Angle	Purity1 Threshold	Purity1 Flag
1	Compound 1d	5,639	3274981	234313	0,084	0,320	No

Precision 1_Accuracy_1d_Report

Reported by User: Roxana Roman (Roxana_Roman)
Acquisition Server Waters7
Project Name: Test
Sample Set Name: Precision 1_Accuracy_PyridineM
Code column: Inertsil ODS-3, 4,6*250 mm, 5 um

SAMPLE INFORMATION

Sample Name:	Methyl-pyridine 40 ug/ mL		
Sample Type:	Unknown	Date Acquired:	12.06.2022 06:36:09
Vial:	39	Acq. Method Set:	Precision 1_Accuracy_PyridineM
Injection #:	1	Date Processed:	18.02.2023 09:26:37
Injection Volume:	100,00 ul	Processing Method:	Precision 1_Accuracy_PyridineM
Run Time:	20,0 Minutes	Channel Name:	Extract 275,0
Acquired By:	Roxana_Roman	Proc. Chnl. Descr.:	PDA 275,0 nm, Smoothed by 25

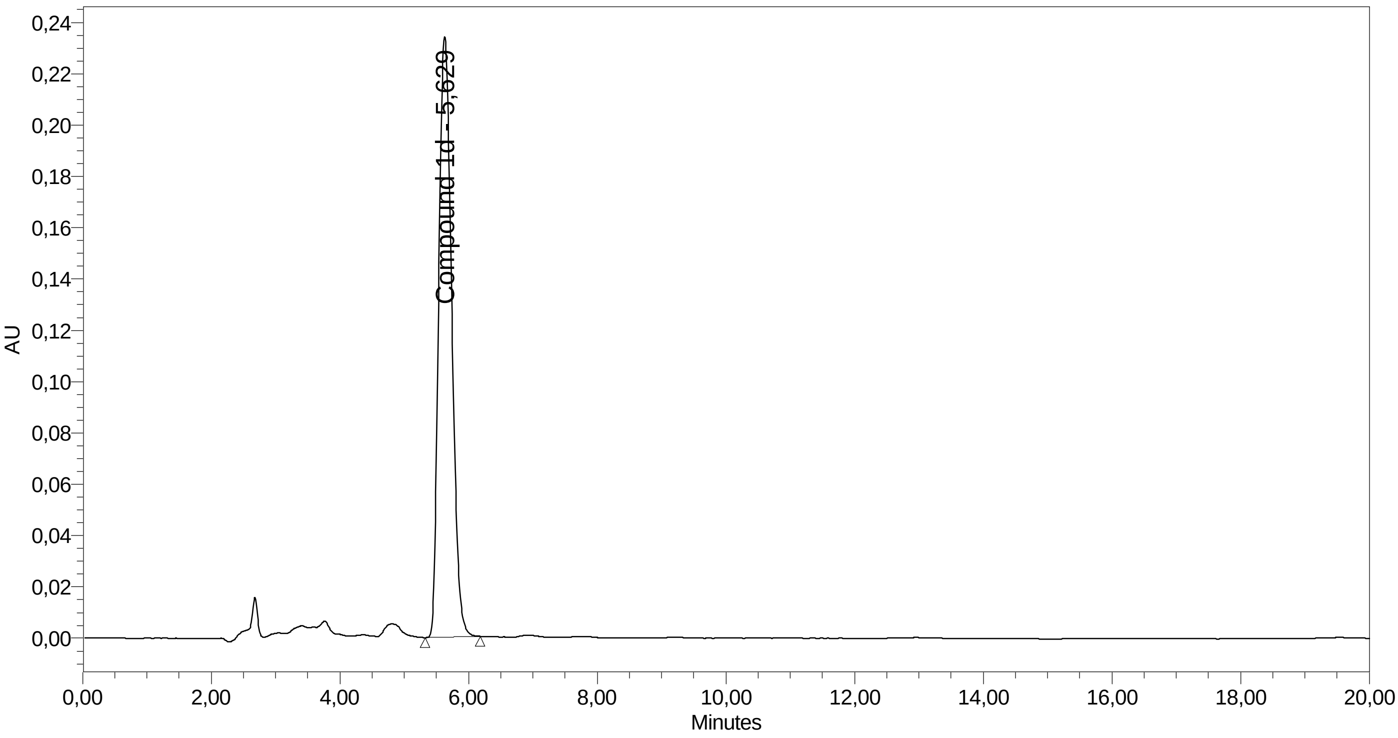

	Peak Name	RT	Area	Height (µV)	Purity1 Angle	Purity1 Threshold	Purity1 Flag
1	Compound 1d	5,629	3276413	234016	0,173	0,288	No

Precision 1_Accuracy_1d_Report

Reported by User: Roxana Roman (Roxana_Roman)
Acquisition Server Waters7
Project Name: Test
Sample Set Name: Precision 1_Accuracy_PyridineM
Code column: Inertsil ODS-3, 4,6*250 mm, 5 um

SAMPLE INFORMATION

Sample Name:	Methyl-pyridine 40 ug/ mL		
Sample Type:	Unknown	Date Acquired:	12.06.2022 06:56:57
Vial:	40	Acq. Method Set:	Precision 1_Accuracy_PyridineM
Injection #:	1	Date Processed:	16.02.2023 13:14:42
Injection Volume:	100,00 ul	Processing Method:	Precision 1_Accuracy_PyridineM
Run Time:	20,0 Minutes	Channel Name:	Extract 275,0
Acquired By:	Roxana_Roman	Proc. Chnl. Descr.:	PDA 275,0 nm, Smoothed by 25

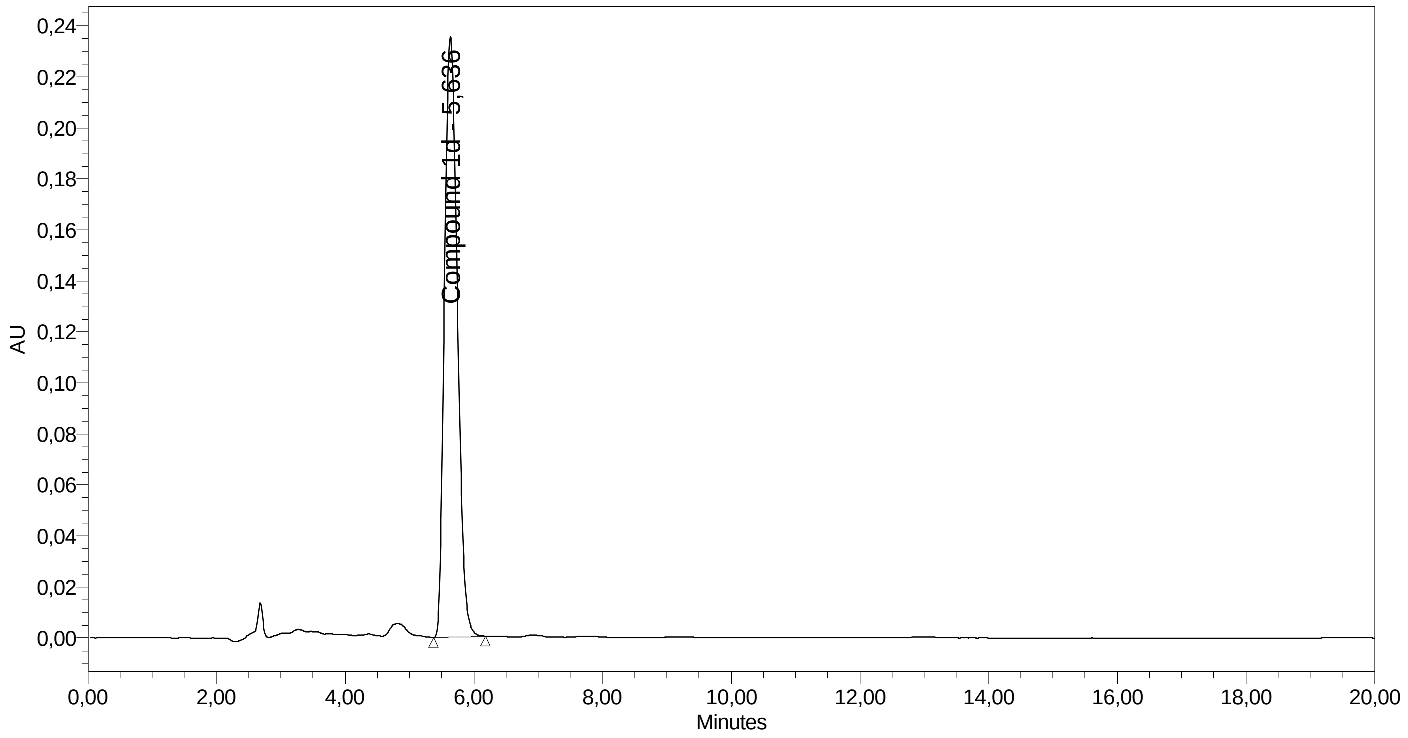

	Peak Name	RT	Area	Height (µV)	Purity1 Angle	Purity1 Threshold	Purity1 Flag
1	Compound 1d	5,636	3288268	235494	0,141	0,286	No

Precision 1_Accuracy_1d_Report

Reported by User: Roxana Roman (Roxana_Roman)
Acquisition Server Waters7
Project Name: Test
Sample Set Name: Precision 1_Accuracy_PyridineM
Code column: Inertsil ODS-3, 4,6*250 mm, 5 um

SAMPLE INFORMATION

Sample Name:	Methyl-pyridine 40 ug/ mL		
Sample Type:	Unknown	Date Acquired:	12.06.2022 07:17:44
Vial:	41	Acq. Method Set:	Precision 1_Accuracy_PyridineM
Injection #:	1	Date Processed:	16.02.2023 13:15:04
Injection Volume:	100,00 ul	Processing Method:	Precision 1_Accuracy_PyridineM
Run Time:	20,0 Minutes	Channel Name:	Extract 275,0
Acquired By:	Roxana_Roman	Proc. Chnl. Descr.:	PDA 275,0 nm, Smoothed by 25

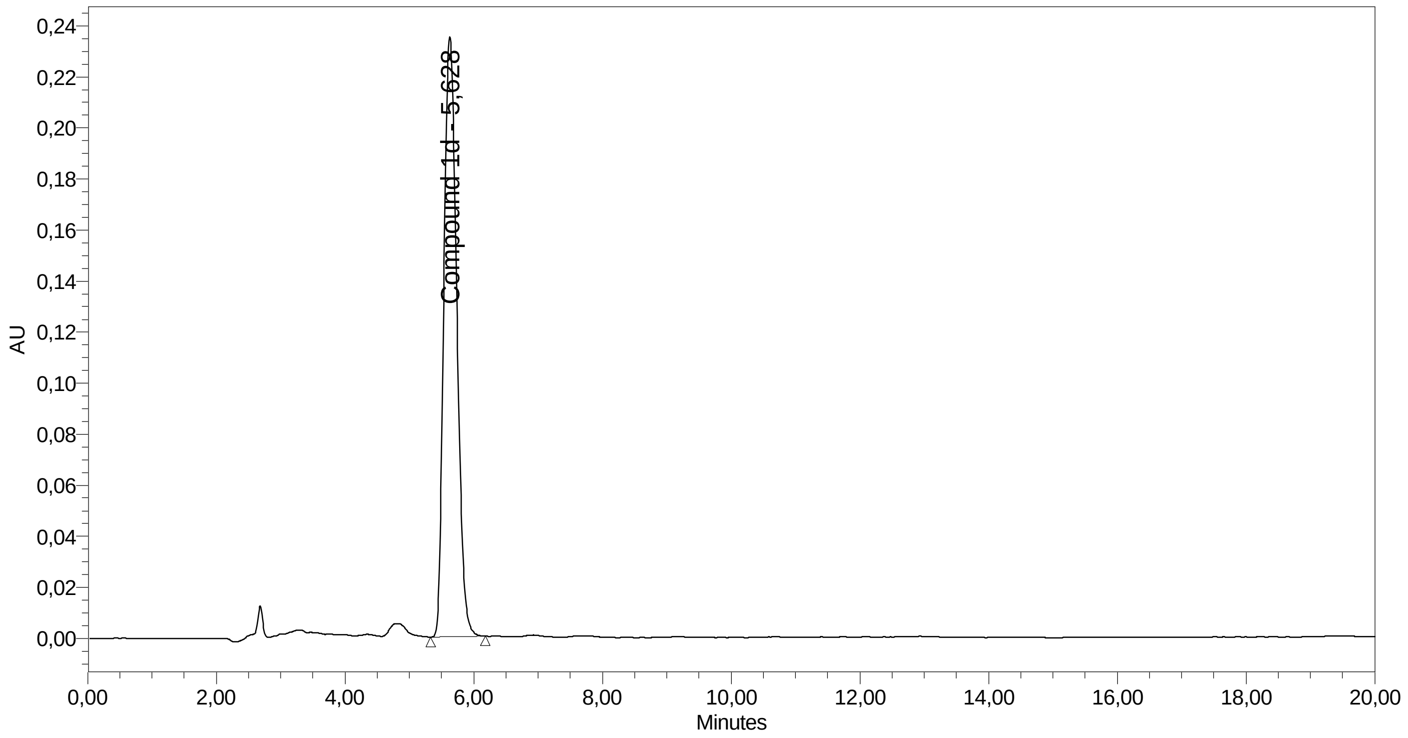

	Peak Name	RT	Area	Height (µV)	Purity1 Angle	Purity1 Threshold	Purity1 Flag
1	Compound 1d	5,628	3281385	235056	0,162	0,288	No

Precision 1_Accuracy_1d_Report

Reported by User: Roxana Roman (Roxana_Roman)
Acquisition Server Waters7
Project Name: Test
Sample Set Name: Precision 1_Accuracy_PyridineM
Code column: Inertsil ODS-3, 4,6*250 mm, 5 um

S A M P L E I N F O R M A T I O N

Sample Name:	Methyl-pyridine 40 ug/ mL		
Sample Type:	Unknown	Date Acquired:	12.06.2022 07:38:32
Vial:	42	Acq. Method Set:	Precision 1_Accuracy_PyridineM
Injection #:	1	Date Processed:	18.02.2023 09:27:02
Injection Volume:	100,00 ul	Processing Method:	Precision 1_Accuracy_PyridineM
Run Time:	20,0 Minutes	Channel Name:	Extract 275,0
Acquired By:	Roxana_Roman	Proc. Chnl. Descr.:	PDA 275,0 nm, Smoothed by 25

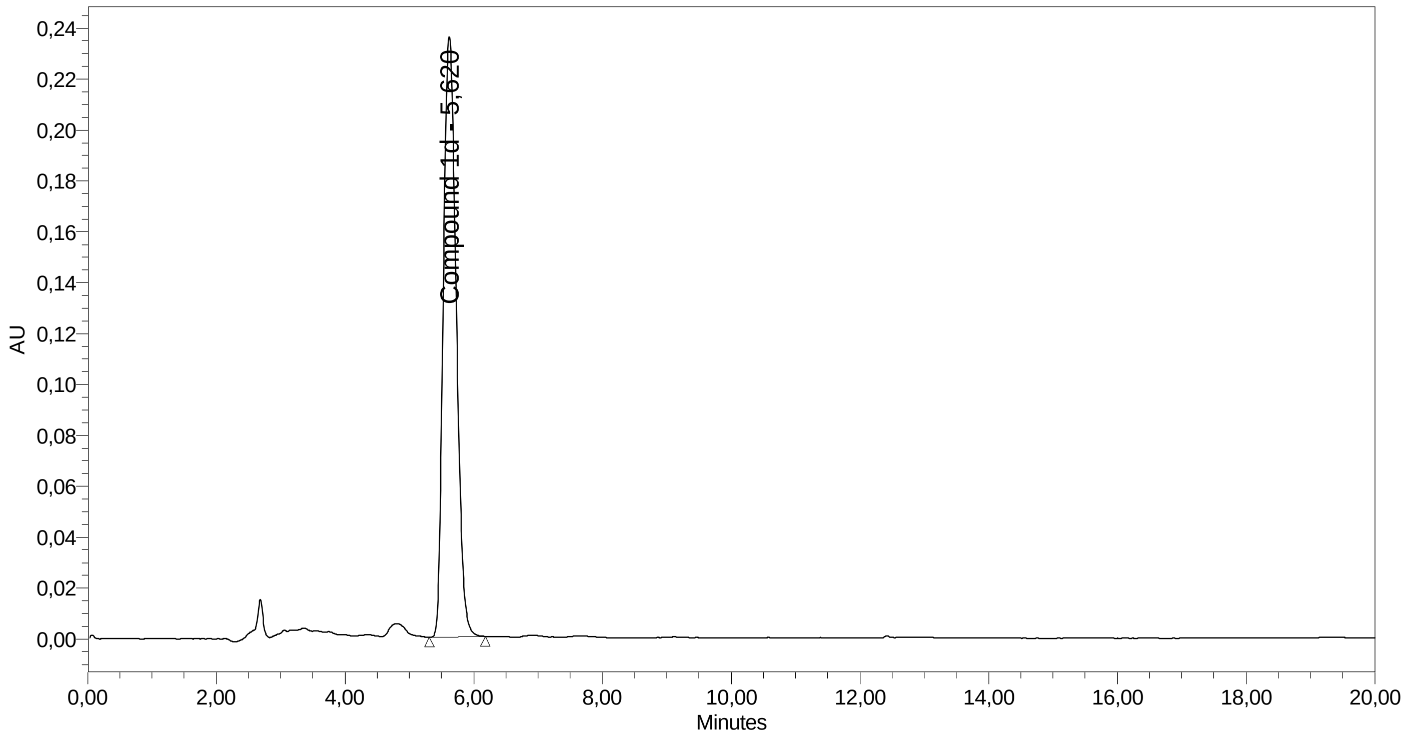

	Peak Name	RT	Area	Height (µV)	Purity1 Angle	Purity1 Threshold	Purity1 Flag
1	Compound 1d	5,620	3287538	235920	0,186	0,287	No

Precision 1_Accuracy_1d_Report

Reported by User: Roxana Roman (Roxana_Roman)
Acquisition Server Waters7
Project Name: Test
Sample Set Name: Precision 1_Accuracy_PyridineM
Code column: Inertsil ODS-3, 4,6*250 mm, 5 um

SAMPLE INFORMATION

Sample Name:	Methyl-pyridine 40 ug/ mL		
Sample Type:	Unknown	Date Acquired:	12.06.2022 07:59:20
Vial:	43	Acq. Method Set:	Precision 1_Accuracy_PyridineM
Injection #:	1	Date Processed:	18.02.2023 09:27:09
Injection Volume:	100,00 ul	Processing Method:	Precision 1_Accuracy_PyridineM
Run Time:	20,0 Minutes	Channel Name:	Extract 275,0
Acquired By:	Roxana_Roman	Proc. Chnl. Descr.:	PDA 275,0 nm, Smoothed by 25

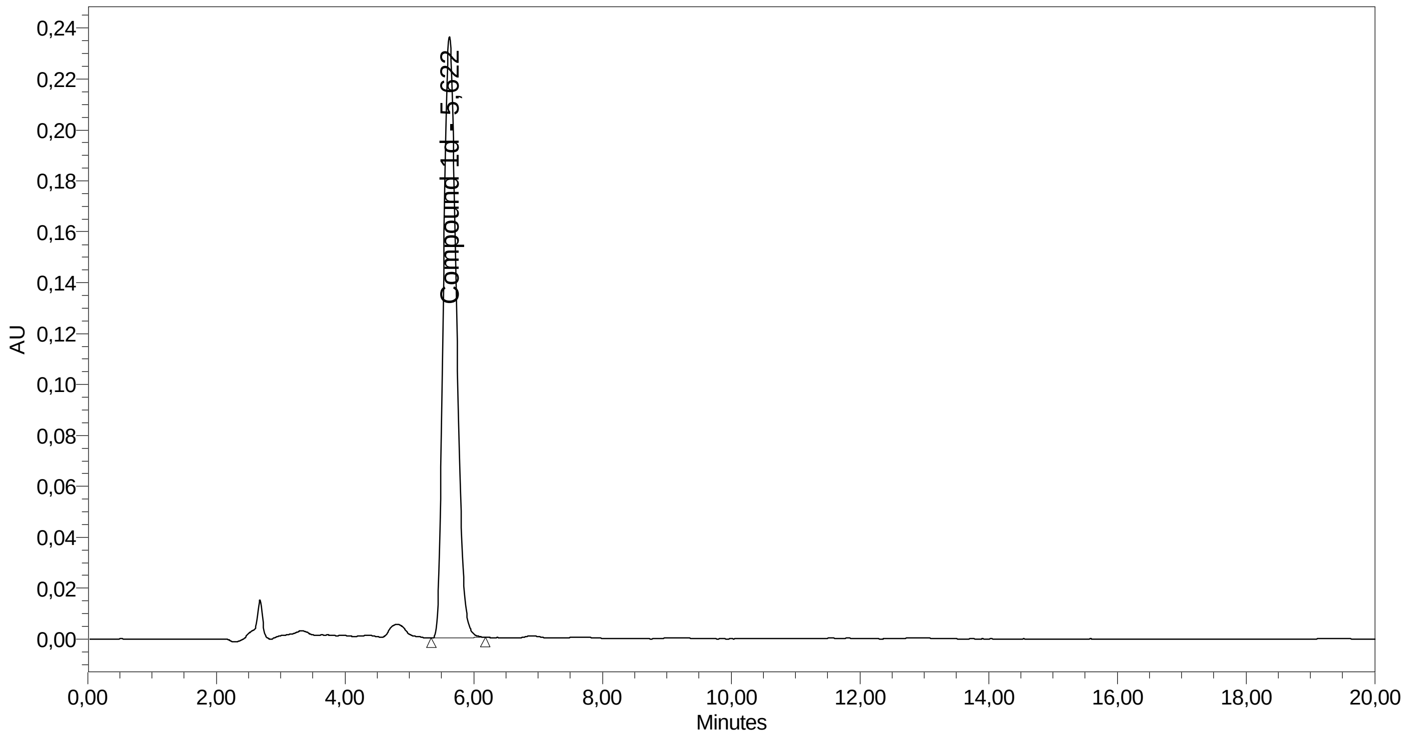

	Peak Name	RT	Area	Height (µV)	Purity1 Angle	Purity1 Threshold	Purity1 Flag
1	Compound 1d	5,622	3288522	236135	0,161	0,287	No

Quantitative determination of

compound 1d

- Validation of the analytical method -

Validation parameters:

- Specificity
- Precision 1
- **Precision 2**
- LOD – LOQ
- Linearity
- Range
- **Accuracy**

Precision 2_1d_Report

Reported by User: Roxana Roman (Roxana_Roman)
Acquisition Server Waters7
Project Name: Test
Sample Set Name: Precision 2_PyridineM
Code column: Inertsil ODS-3, 4,6*250 mm, 5 um

SAMPLE INFORMATION

Sample Name:	Methyl-pyridine 30 ug/ mL		
Sample Type:	Standard	Date Acquired:	15.06.2022 13:04:35
Vial:	25	Acq. Method Set:	Precision 2_PyridineM
Injection #:	1	Date Processed:	18.02.2023 09:43:00
Injection Volume:	100,00 ul	Processing Method:	Precision 2_PyridineM
Run Time:	20,0 Minutes	Channel Name:	Extract 275,0
Acquired By:	Roxana_Roman	Proc. Chnl. Descr.:	PDA 275,0 nm, Smoothed by 25

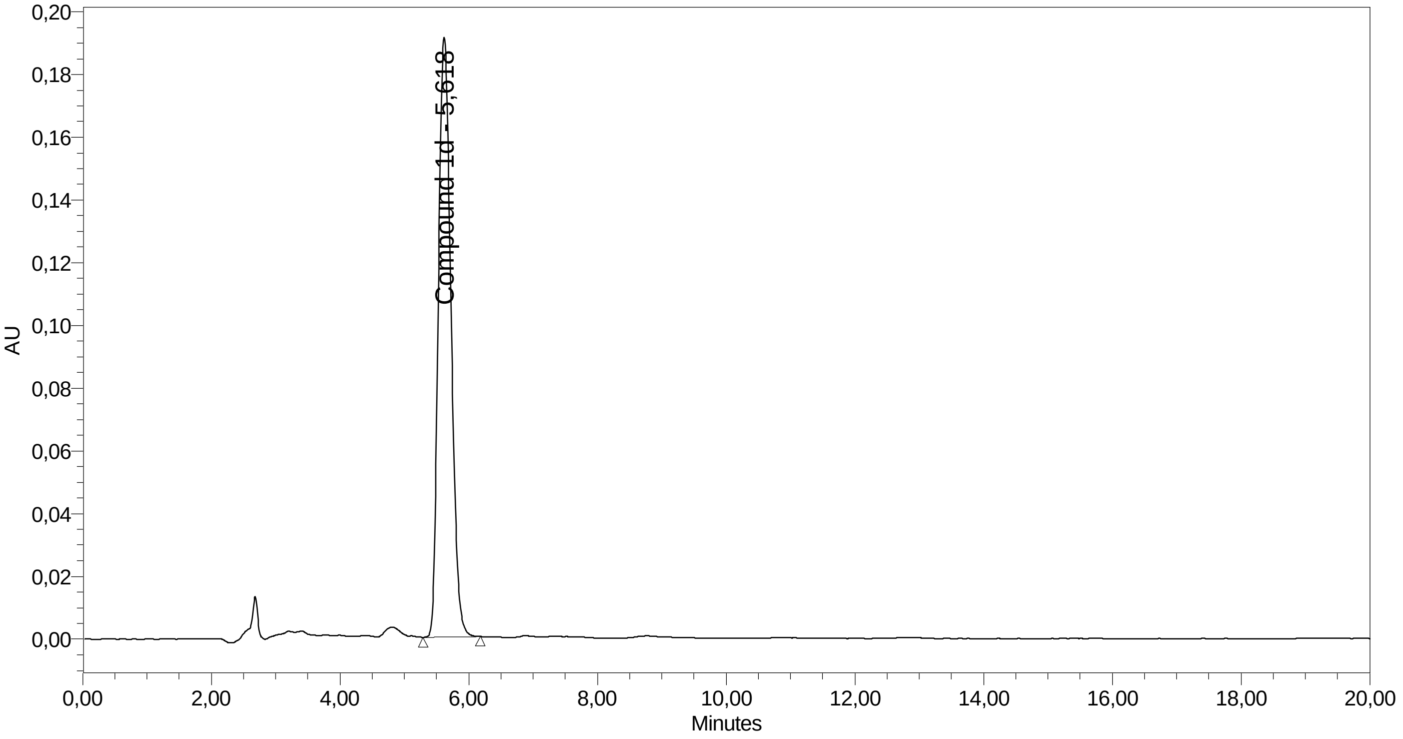

	Peak Name	RT	Area	Height (µV)	Purity1 Angle	Purity1 Threshold	Purity1 Flag
1	Compound 1d	5,618	2598931	191148	0,282	0,313	No

Precision 2_1d_Report

Reported by User: Roxana Roman (Roxana_Roman)
Acquisition Server Waters7
Project Name: Test
Sample Set Name: Precision 2_PyridineM
Code column: Inertsil ODS-3, 4,6*250 mm, 5 um

SAMPLE INFORMATION

Sample Name:	Methyl-pyridine 30 ug/ mL		
Sample Type:	Standard	Date Acquired:	15.06.2022 13:25:19
Vial:	25	Acq. Method Set:	Precision 2_PyridineM
Injection #:	2	Date Processed:	18.02.2023 09:43:08
Injection Volume:	100,00 ul	Processing Method:	Precision 2_PyridineM
Run Time:	20,0 Minutes	Channel Name:	Extract 275,0
Acquired By:	Roxana_Roman	Proc. Chnl. Descr.:	PDA 275,0 nm, Smoothed by 25

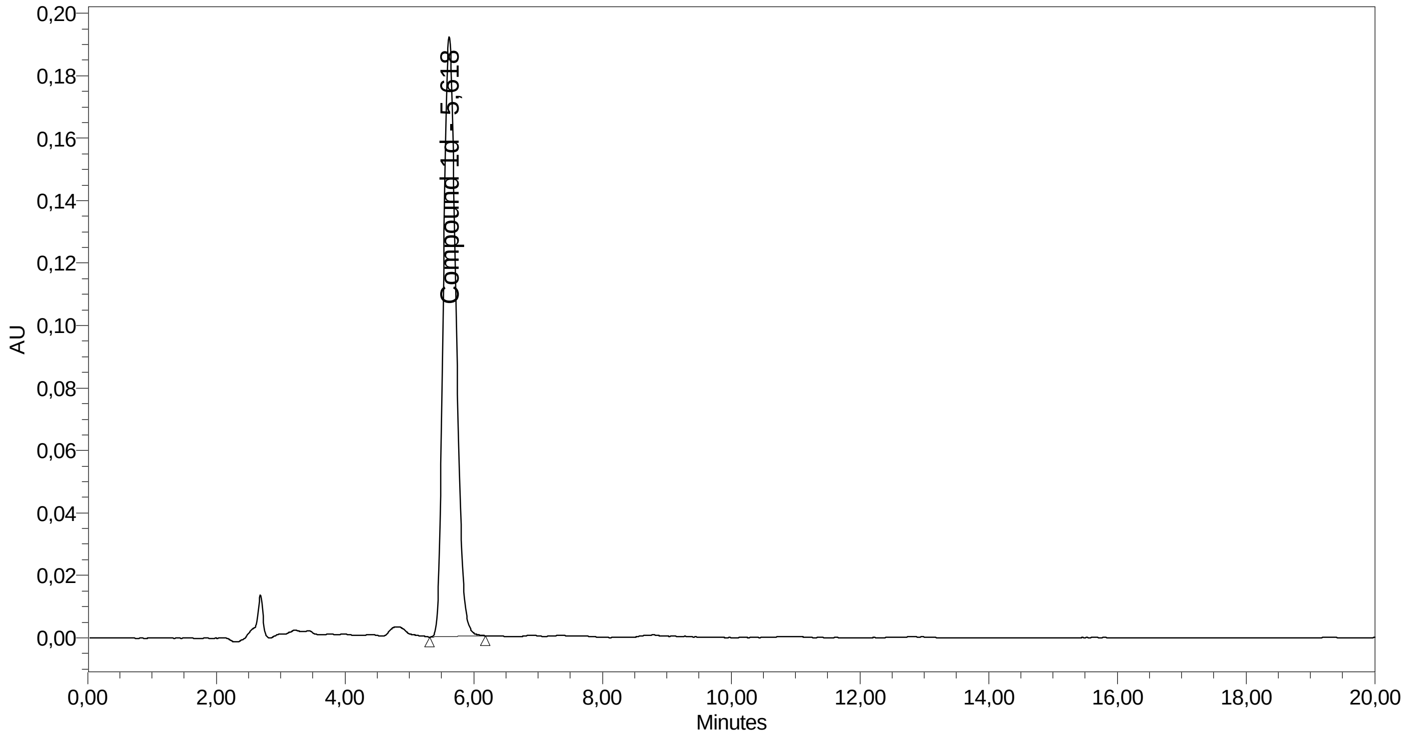

	Peak Name	RT	Area	Height (µV)	Purity1 Angle	Purity1 Threshold	Purity1 Flag
1	Compound 1d	5,618	2607971	192018	0,235	0,288	No

Precision 2_1d_Report

Reported by User: Roxana Roman (Roxana_Roman)
Acquisition Server Waters7
Project Name: Test
Sample Set Name: Precision 2_PyridineM
Code column: Inertsil ODS-3, 4,6*250 mm, 5 um

SAMPLE INFORMATION

Sample Name:	Methyl-pyridine 30 ug/ mL		
Sample Type:	Standard	Date Acquired:	15.06.2022 13:46:02
Vial:	25	Acq. Method Set:	Precision 2_PyridineM
Injection #:	3	Date Processed:	18.02.2023 09:43:08
Injection Volume:	100,00 ul	Processing Method:	Precision 2_PyridineM
Run Time:	20,0 Minutes	Channel Name:	Extract 275,0
Acquired By:	Roxana_Roman	Proc. Chnl. Descr.:	PDA 275,0 nm, Smoothed by 25

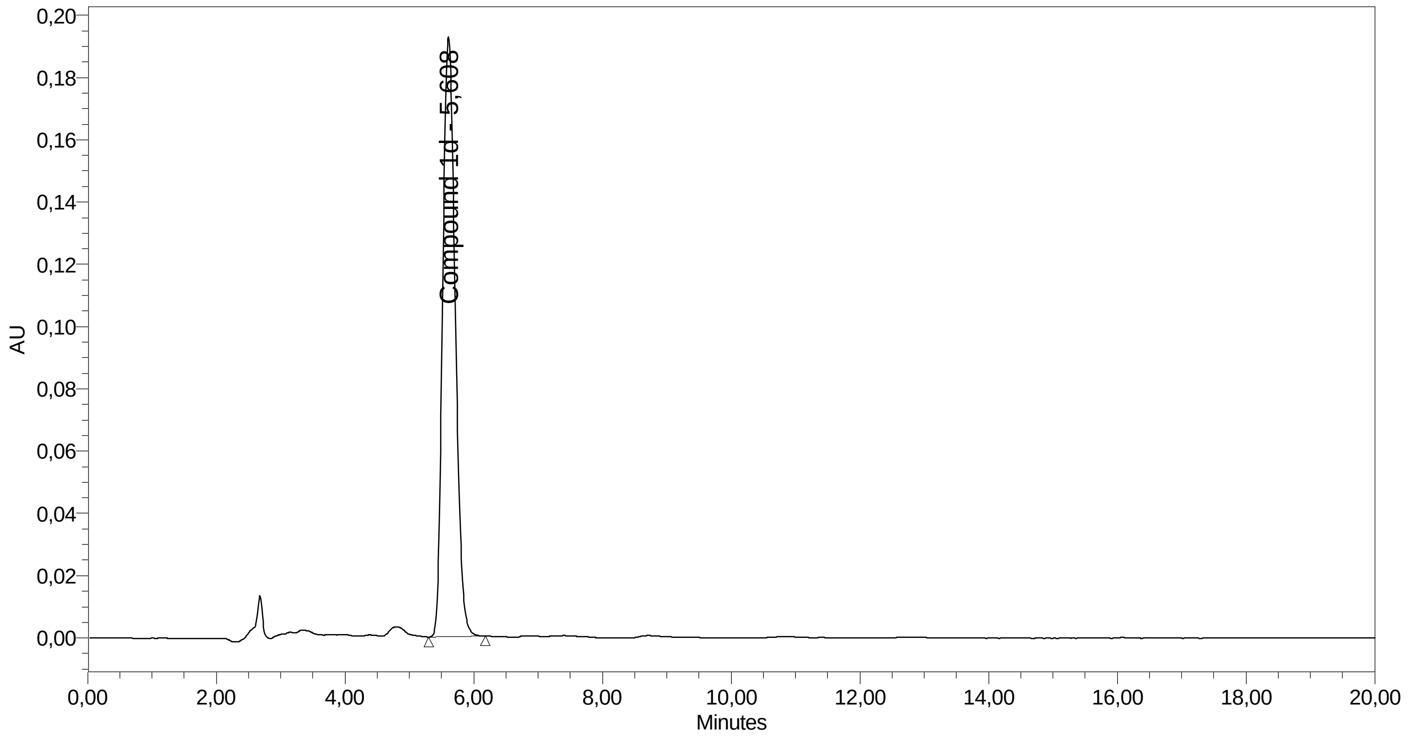

	Peak Name	RT	Area	Height (µV)	Purity1 Angle	Purity1 Threshold	Purity1 Flag
1	Compound 1d	5,608	2616204	192615	0,233	0,285	No

Component Summary Area Time

Reported by User: Roxana Roman (Roxana_Roman)
Acquisition Server Waters7
Project Name: Test
Sample Set Name: Precision 2_PyridineM
Code column: Inertsil ODS-3, 4,6*250 mm, 5 um

Component Summary For Retention Time
Channel: W2996

	SampleName	Inj	Channel	Vial	Compound 1d
1	Methyl-pyridine 30 ug/ mL	1	W2996	25	5,618
2	Methyl-pyridine 30 ug/ mL	2	W2996	25	5,618
3	Methyl-pyridine 30 ug/ mL	3	W2996	25	5,608
Mean					5,615
Std. Dev.					0,006
% RSD					0,11

Component Summary For Area
Channel: W2996

	SampleName	Inj	Channel	Vial	Compound 1d
1	Methyl-pyridine 30 ug/ mL	1	W2996	25	2598931
2	Methyl-pyridine 30 ug/ mL	2	W2996	25	2607971
3	Methyl-pyridine 30 ug/ mL	3	W2996	25	2616204
Mean					2607702
Std. Dev.					8640
% RSD					0,33

Precision 2_1d_Report

Reported by User: Roxana Roman (Roxana_Roman)
Acquisition Server Waters7
Project Name: Test
Sample Set Name: Precision 2_PyridineM
Code column: Inertsil ODS-3, 4,6*250 mm, 5 um

SAMPLE INFORMATION

Sample Name:	Methanol		
Sample Type:	Standard	Date Acquired:	15.06.2022 12:43:32
Vial:	11	Acq. Method Set:	Precision 2_PyridineM
Injection #:	1	Date Processed:	18.02.2023 09:42:52
Injection Volume:	100,00 ul	Processing Method:	Precision 2_PyridineM
Run Time:	20,0 Minutes	Channel Name:	Extract 275,0
Acquired By:	Roxana_Roman	Proc. Chnl. Descr.:	PDA 275,0 nm, Smoothed by 25

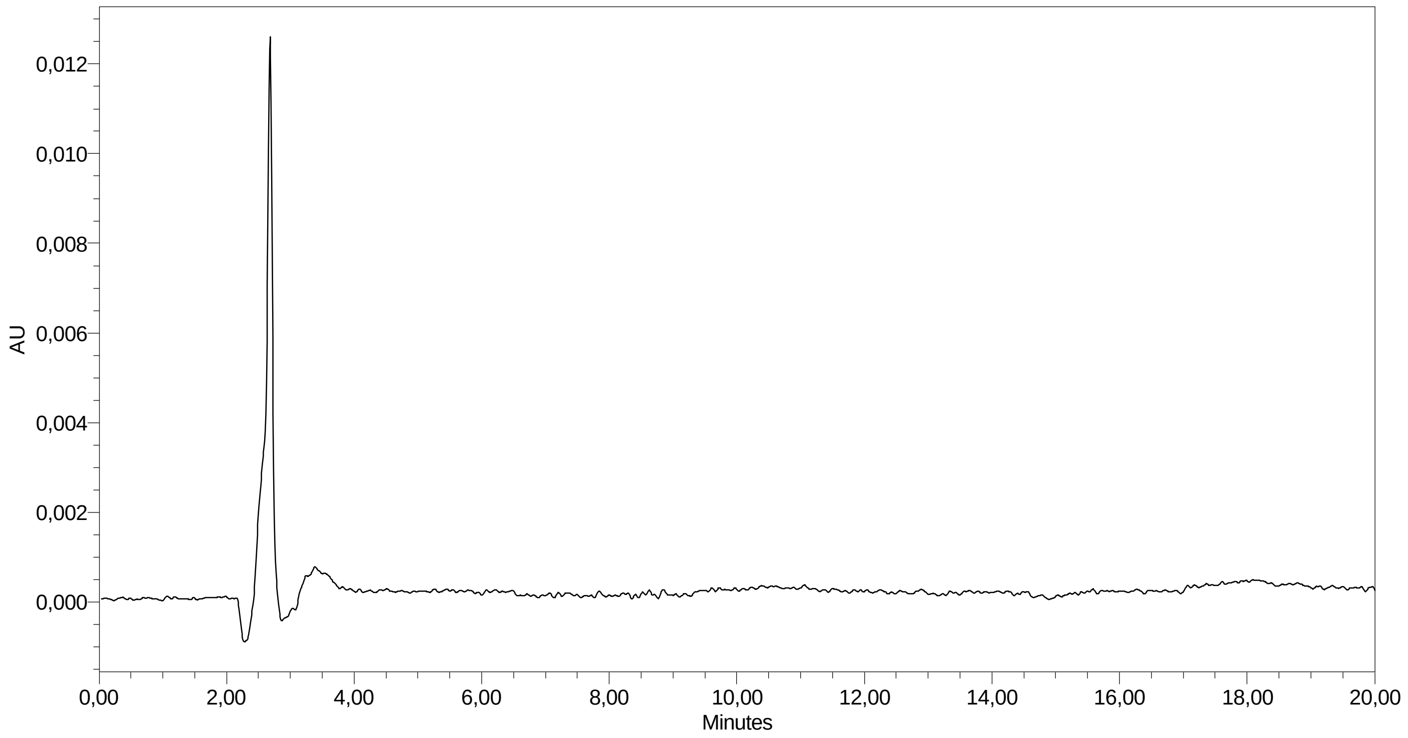

Basic LC Peaks Table group contains no data.

Precision 2_1d_Report

Reported by User: Roxana Roman (Roxana_Roman)
Acquisition Server Waters7
Project Name: Test
Sample Set Name: Precision 2_PyridineM
Code column: Inertsil ODS-3, 4,6*250 mm, 5 um

SAMPLE INFORMATION

Sample Name:	Methyl-pyridine 20 ug/ mL		
Sample Type:	Unknown	Date Acquired:	15.06.2022 15:56:17
Vial:	26	Acq. Method Set:	Precision 2_PyridineM
Injection #:	1	Date Processed:	18.02.2023 09:44:24
Injection Volume:	100,00 ul	Processing Method:	Precision 2_PyridineM
Run Time:	20,0 Minutes	Channel Name:	Extract 275,0
Acquired By:	Roxana_Roman	Proc. Chnl. Descr.:	PDA 275,0 nm, Smoothed by 25

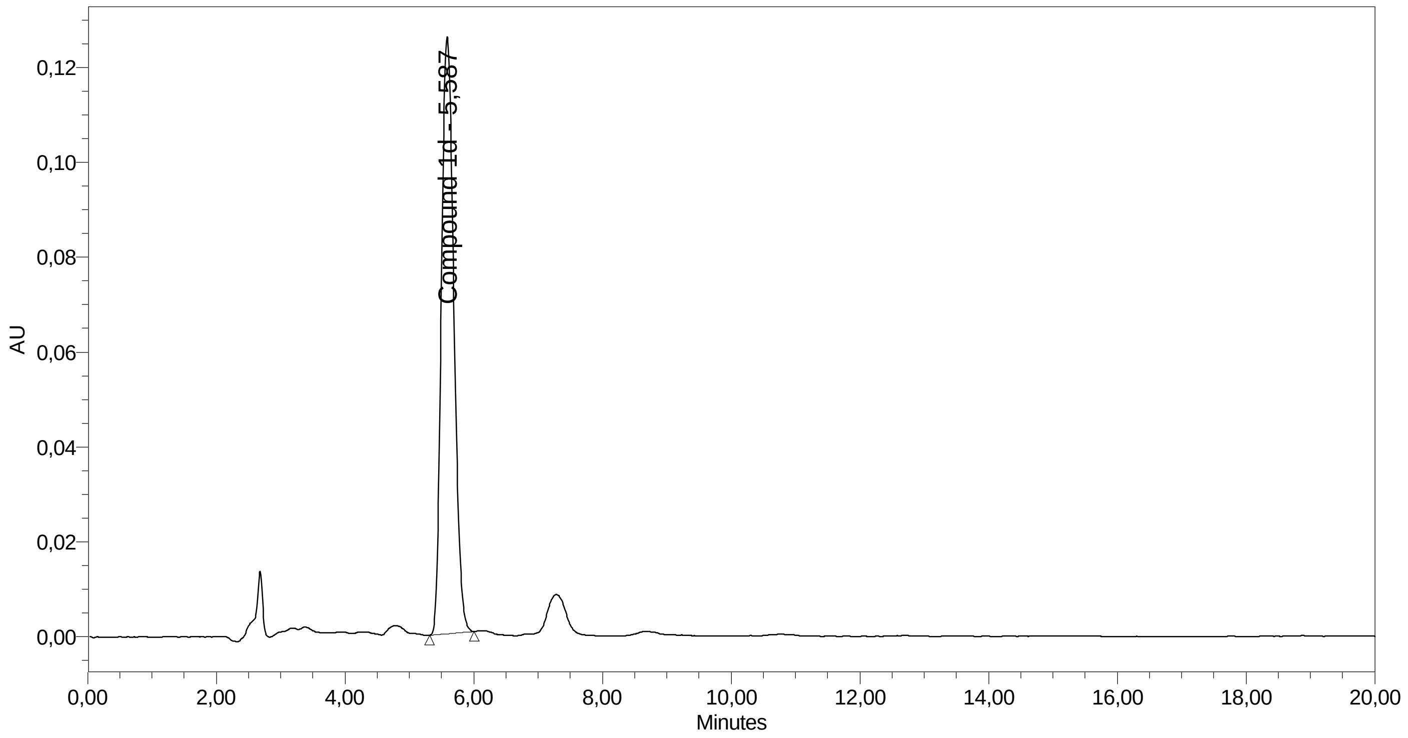

	Peak Name	RT	Area	Height (µV)	Purity1 Angle	Purity1 Threshold	Purity1 Flag
1	Compound 1d	5,587	1691833	125886	0,277	0,314	No

Precision 2_1d_Report

Reported by User: Roxana Roman (Roxana_Roman)
Acquisition Server Waters7
Project Name: Test
Sample Set Name: Precision 2_PyridineM
Code column: Inertsil ODS-3, 4,6*250 mm, 5 um

SAMPLE INFORMATION

Sample Name:	Methyl-pyridine 20 ug/ mL		
Sample Type:	Unknown	Date Acquired:	15.06.2022 14:52:49
Vial:	27	Acq. Method Set:	Precision 2_PyridineM
Injection #:	1	Date Processed:	18.02.2023 09:45:21
Injection Volume:	100,00 ul	Processing Method:	Precision 2_PyridineM
Run Time:	20,0 Minutes	Channel Name:	Extract 275,0
Acquired By:	Roxana_Roman	Proc. Chnl. Descr.:	PDA 275,0 nm, Smoothed by 25

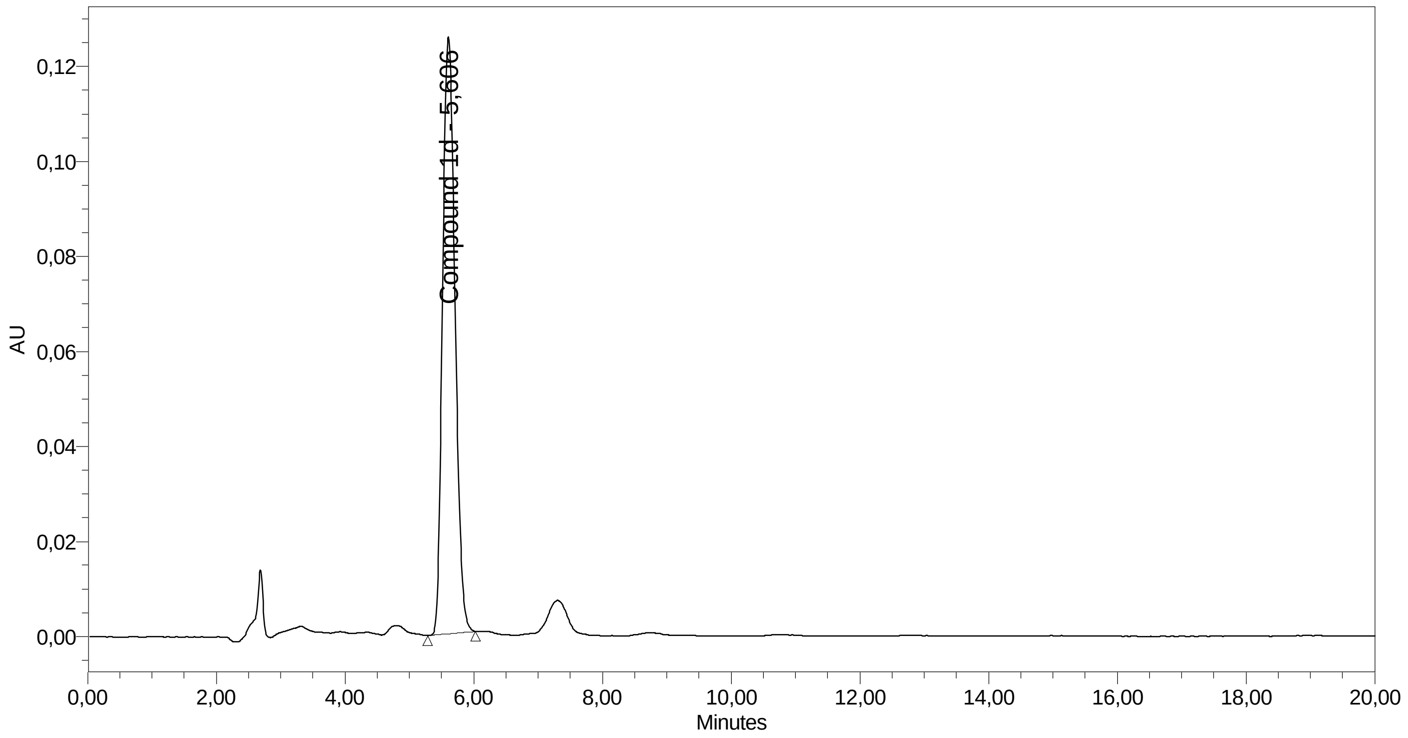

	Peak Name	RT	Area	Height (µV)	Purity1 Angle	Purity1 Threshold	Purity1 Flag
1	Compound 1d	5,606	1690608	125674	0,268	0,286	No

Precision 2_1d_Report

Reported by User: Roxana Roman (Roxana_Roman)
Acquisition Server Waters7
Project Name: Test
Sample Set Name: Precision 2_PyridineM
Code column: Inertsil ODS-3, 4,6*250 mm, 5 um

SAMPLE INFORMATION

Sample Name:	Methyl-pyridine 20 ug/ mL		
Sample Type:	Unknown	Date Acquired:	15.06.2022 15:13:37
Vial:	28	Acq. Method Set:	Precision 2_PyridineM
Injection #:	1	Date Processed:	18.02.2023 09:49:25
Injection Volume:	100,00 ul	Processing Method:	Precision 2_PyridineM
Run Time:	20,0 Minutes	Channel Name:	Extract 275,0
Acquired By:	Roxana_Roman	Proc. Chnl. Descr.:	PDA 275,0 nm, Smoothed by 25

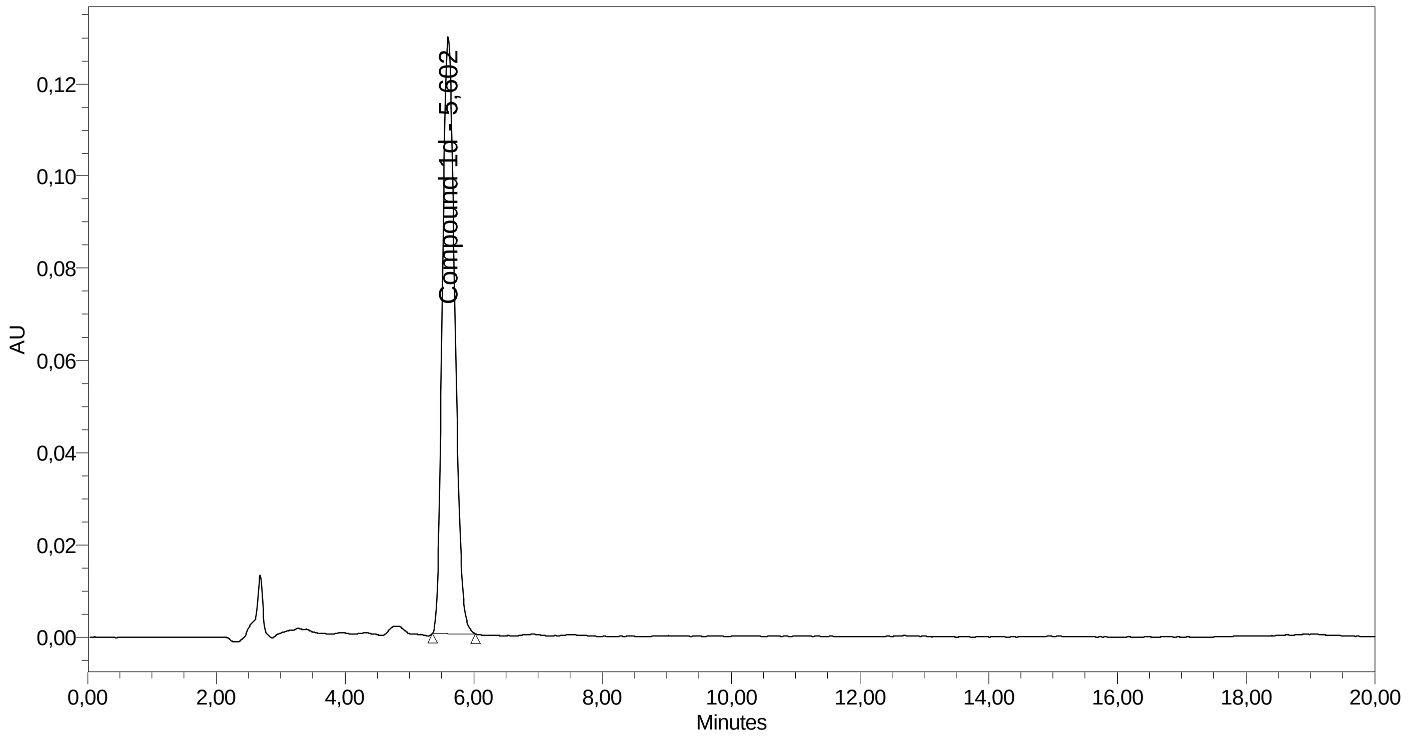

	Peak Name	RT	Area	Height (µV)	Purity1 Angle	Purity1 Threshold	Purity1 Flag
1	Compound 1d	5,602	1736221	129434	0,222	0,285	No

Precision 2_1d_Report

Reported by User: Roxana Roman (Roxana_Roman)
Acquisition Server Waters7
Project Name: Test
Sample Set Name: Precision 2_PyridineM
Code column: Inertsil ODS-3, 4,6*250 mm, 5 um

SAMPLE INFORMATION

Sample Name:	Methyl-pyridine 20 ug/ mL		
Sample Type:	Unknown	Date Acquired:	15.06.2022 15:34:26
Vial:	29	Acq. Method Set:	Precision 2_PyridineM
Injection #:	1	Date Processed:	18.02.2023 09:45:51
Injection Volume:	100,00 ul	Processing Method:	Precision 2_PyridineM
Run Time:	20,0 Minutes	Channel Name:	Extract 275,0
Acquired By:	Roxana_Roman	Proc. Chnl. Descr.:	PDA 275,0 nm, Smoothed by 25

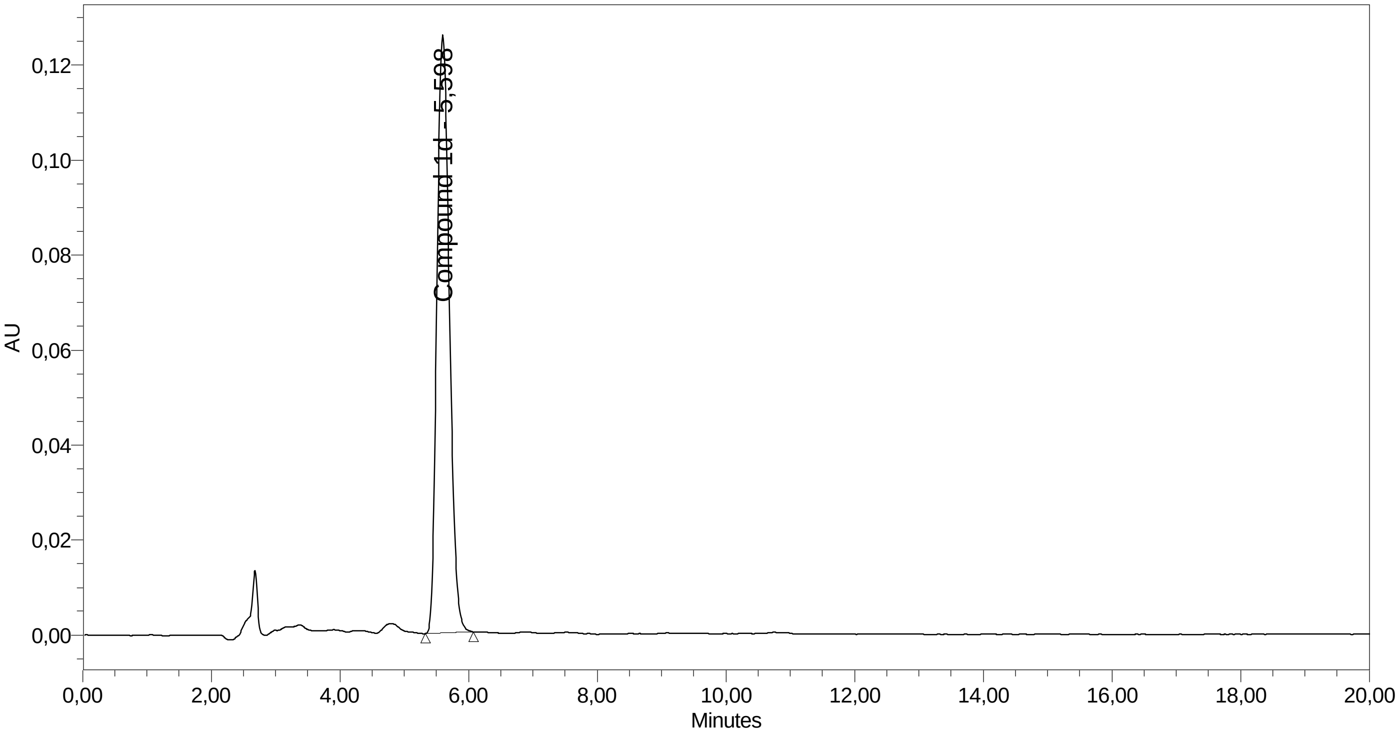

	Peak Name	RT	Area	Height (µV)	Purity1 Angle	Purity1 Threshold	Purity1 Flag
1	Compound 1d	5,598	1697748	125914	0,285	0,286	No

Precision 2_1d_Report

Reported by User: Roxana Roman (Roxana_Roman)
Acquisition Server Waters7
Project Name: Test
Sample Set Name: Precision 2_PyridineM
Code column: Inertsil ODS-3, 4,6*250 mm, 5 um

SAMPLE INFORMATION

Sample Name:	Methyl-pyridine 20 ug/ mL		
Sample Type:	Unknown	Date Acquired:	15.06.2022 16:17:06
Vial:	30	Acq. Method Set:	Precision 2_PyridineM
Injection #:	1	Date Processed:	18.02.2023 09:46:52
Injection Volume:	100,00 ul	Processing Method:	Precision 2_PyridineM
Run Time:	20,0 Minutes	Channel Name:	Extract 275,0
Acquired By:	Roxana_Roman	Proc. Chnl. Descr.:	PDA 275,0 nm, Smoothed by 25

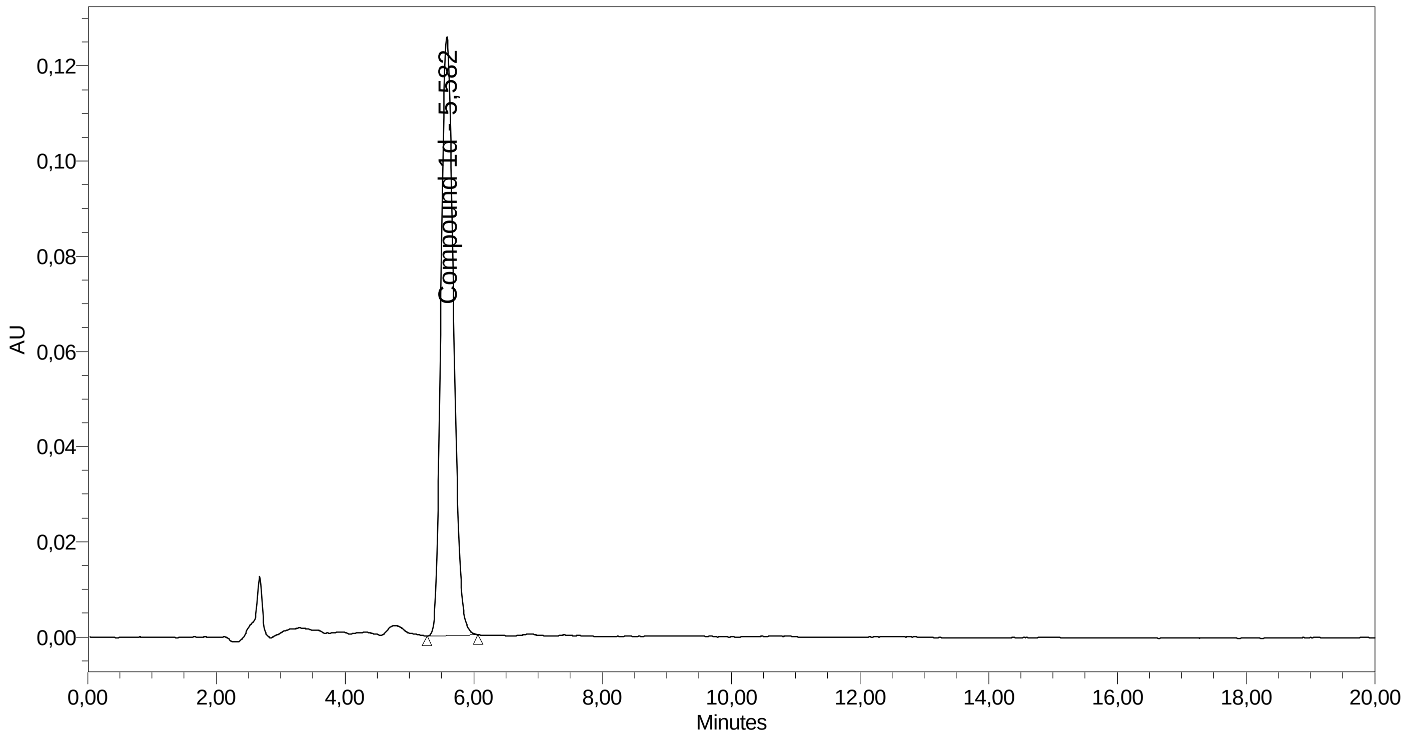

	Peak Name	RT	Area	Height (µV)	Purity1 Angle	Purity1 Threshold	Purity1 Flag
1	Compound 1d	5,582	1704577	125772	0,281	0,288	No

Precision 2_1d_Report

Reported by User: Roxana Roman (Roxana_Roman)
Acquisition Server Waters7
Project Name: Test
Sample Set Name: Precision 2_PyridineM
Code column: Inertsil ODS-3, 4,6*250 mm, 5 um

SAMPLE INFORMATION

Sample Name:	Methyl-pyridine 20 ug/ mL		
Sample Type:	Unknown	Date Acquired:	15.06.2022 16:37:53
Vial:	31	Acq. Method Set:	Precision 2_PyridineM
Injection #:	1	Date Processed:	18.02.2023 09:47:53
Injection Volume:	100,00 ul	Processing Method:	Precision 2_PyridineM
Run Time:	20,0 Minutes	Channel Name:	Extract 275,0
Acquired By:	Roxana_Roman	Proc. Chnl. Descr.:	PDA 275,0 nm, Smoothed by 25

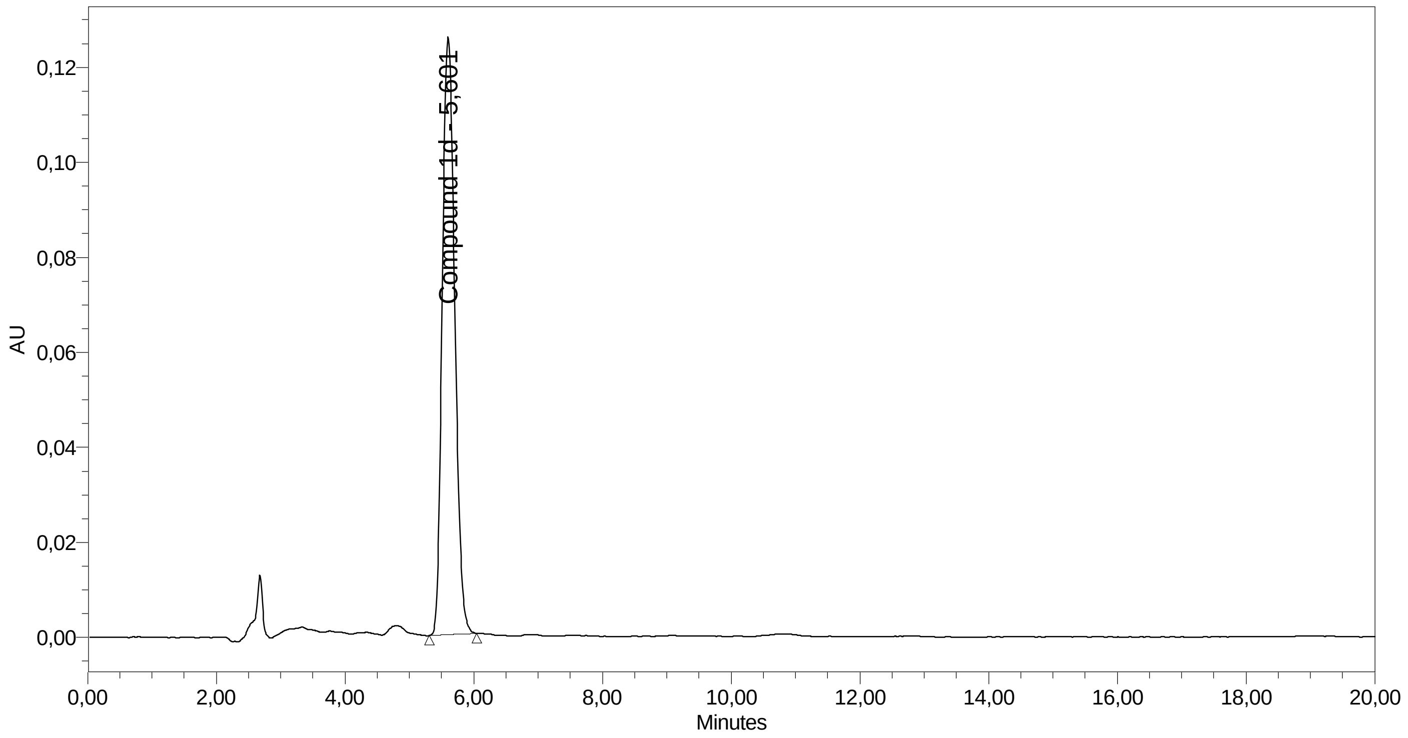

	Peak Name	RT	Area	Height (µV)	Purity1 Angle	Purity1 Threshold	Purity1 Flag
1	Compound 1d	5,601	1697552	125813	0,280	0,281	No

Precision 2_1d_Report

Reported by User: Roxana Roman (Roxana_Roman)
Acquisition Server Waters7
Project Name: Test
Sample Set Name: Precision 2_PyridineM
Code column: Inertsil ODS-3, 4,6*250 mm, 5 um

SAMPLE INFORMATION

Sample Name:	Methyl-pyridine 30 ug/ mL		
Sample Type:	Unknown	Date Acquired:	15.06.2022 16:58:41
Vial:	32	Acq. Method Set:	Precision 2_PyridineM
Injection #:	1	Date Processed:	18.02.2023 09:49:27
Injection Volume:	100,00 ul	Processing Method:	Precision 2_PyridineM
Run Time:	20,0 Minutes	Channel Name:	Extract 275,0
Acquired By:	Roxana_Roman	Proc. Chnl. Descr.:	PDA 275,0 nm, Smoothed by 25

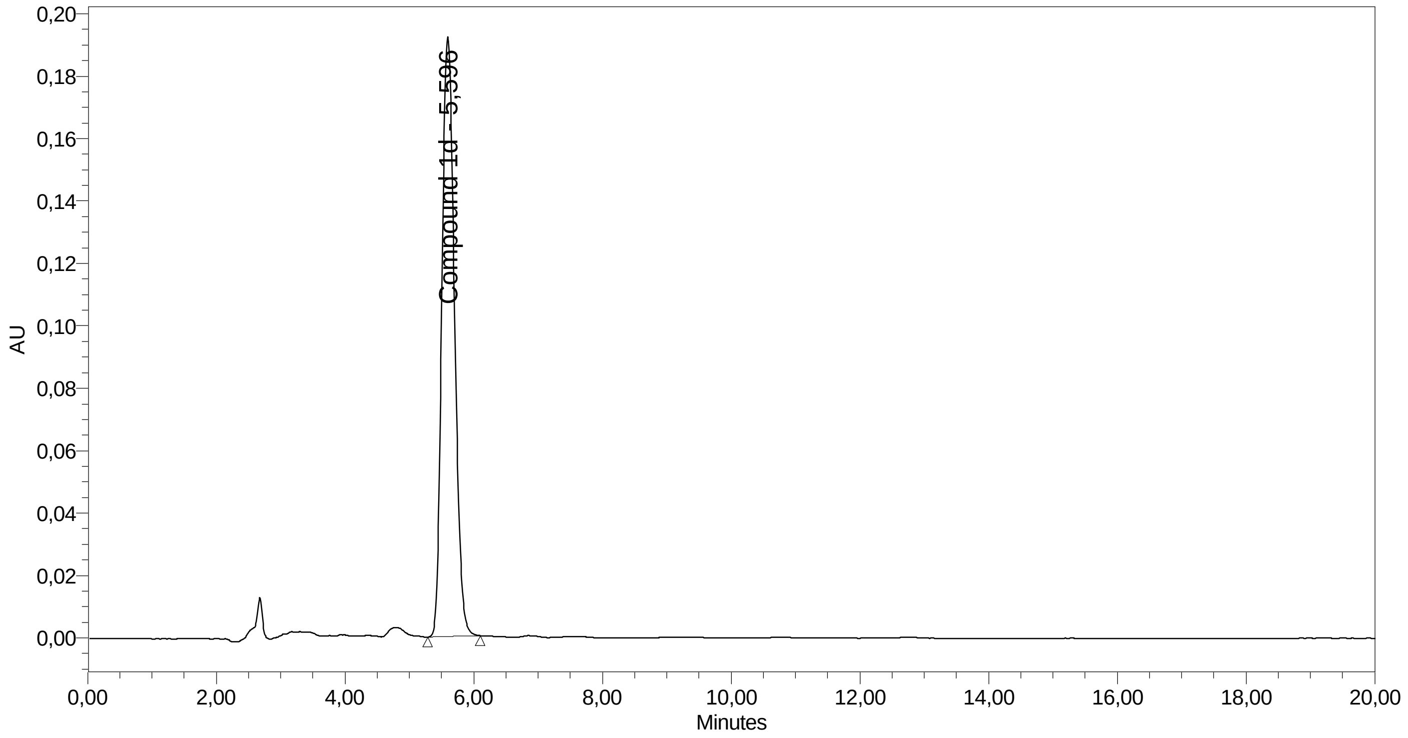

	Peak Name	RT	Area	Height (µV)	Purity1 Angle	Purity1 Threshold	Purity1 Flag
1	Compound 1d	5,596	2614065	192238	0,212	0,282	No

Precision 2_1d_Report

Reported by User: Roxana Roman (Roxana_Roman)
Acquisition Server Waters7
Project Name: Test
Sample Set Name: Precision 2_PyridineM
Code column: Inertsil ODS-3, 4,6*250 mm, 5 um

SAMPLE INFORMATION

Sample Name:	Methyl-pyridine 30 ug/ mL		
Sample Type:	Unknown	Date Acquired:	15.06.2022 17:19:29
Vial:	33	Acq. Method Set:	Precision 2_PyridineM
Injection #:	1	Date Processed:	18.02.2023 09:49:42
Injection Volume:	100,00 ul	Processing Method:	Precision 2_PyridineM
Run Time:	20,0 Minutes	Channel Name:	Extract 275,0
Acquired By:	Roxana_Roman	Proc. Chnl. Descr.:	PDA 275,0 nm, Smoothed by 25

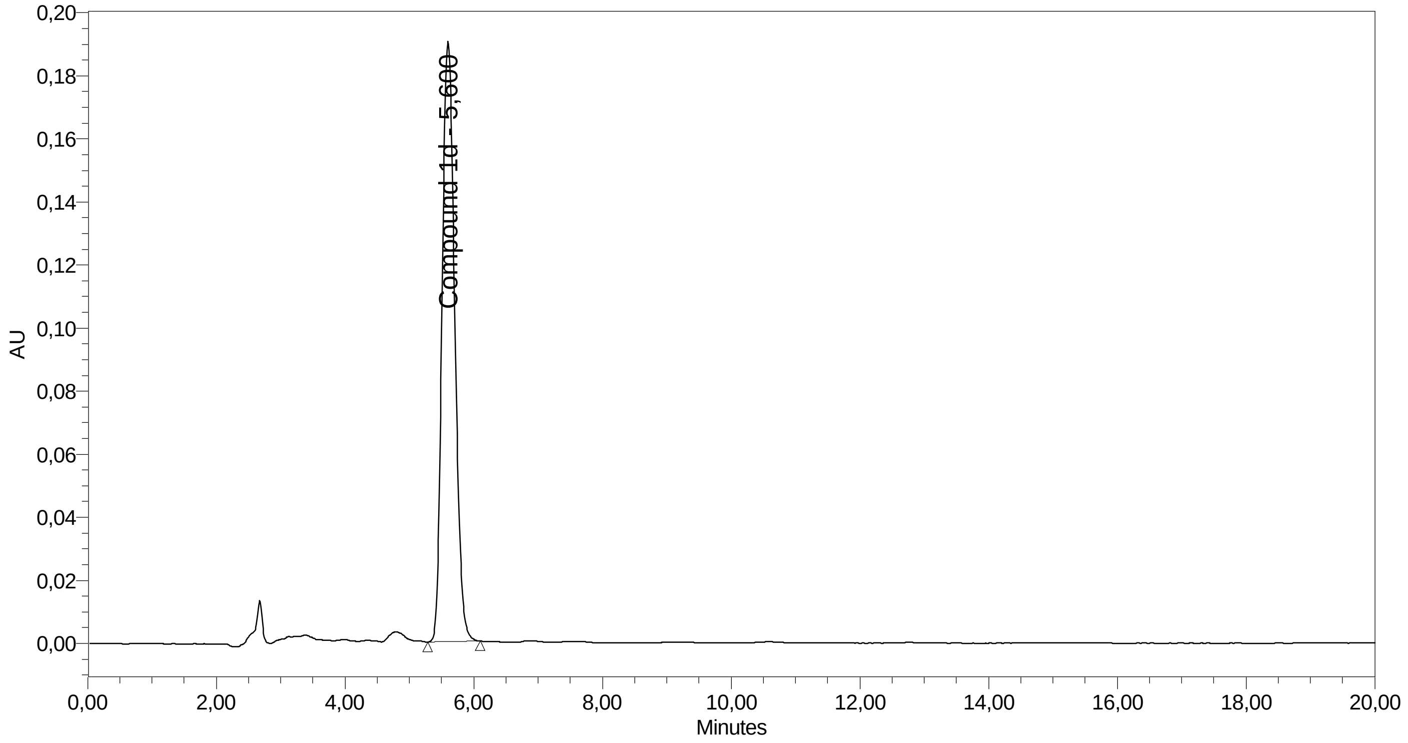

	Peak Name	RT	Area	Height (µV)	Purity1 Angle	Purity1 Threshold	Purity1 Flag
1	Compound 1d	5,600	2594510	190263	0,194	0,284	No

Precision 2_1d_Report

Reported by User: Roxana Roman (Roxana_Roman)
Acquisition Server Waters7
Project Name: Test
Sample Set Name: Precision 2_PyridineM
Code column: Inertsil ODS-3, 4,6*250 mm, 5 um

SAMPLE INFORMATION

Sample Name:	Methyl-pyridine 30 ug/ mL		
Sample Type:	Unknown	Date Acquired:	15.06.2022 17:40:17
Vial:	34	Acq. Method Set:	Precision 2_PyridineM
Injection #:	1	Date Processed:	18.02.2023 09:49:50
Injection Volume:	100,00 ul	Processing Method:	Precision 2_PyridineM
Run Time:	20,0 Minutes	Channel Name:	Extract 275,0
Acquired By:	Roxana_Roman	Proc. Chnl. Descr.:	PDA 275,0 nm, Smoothed by 25

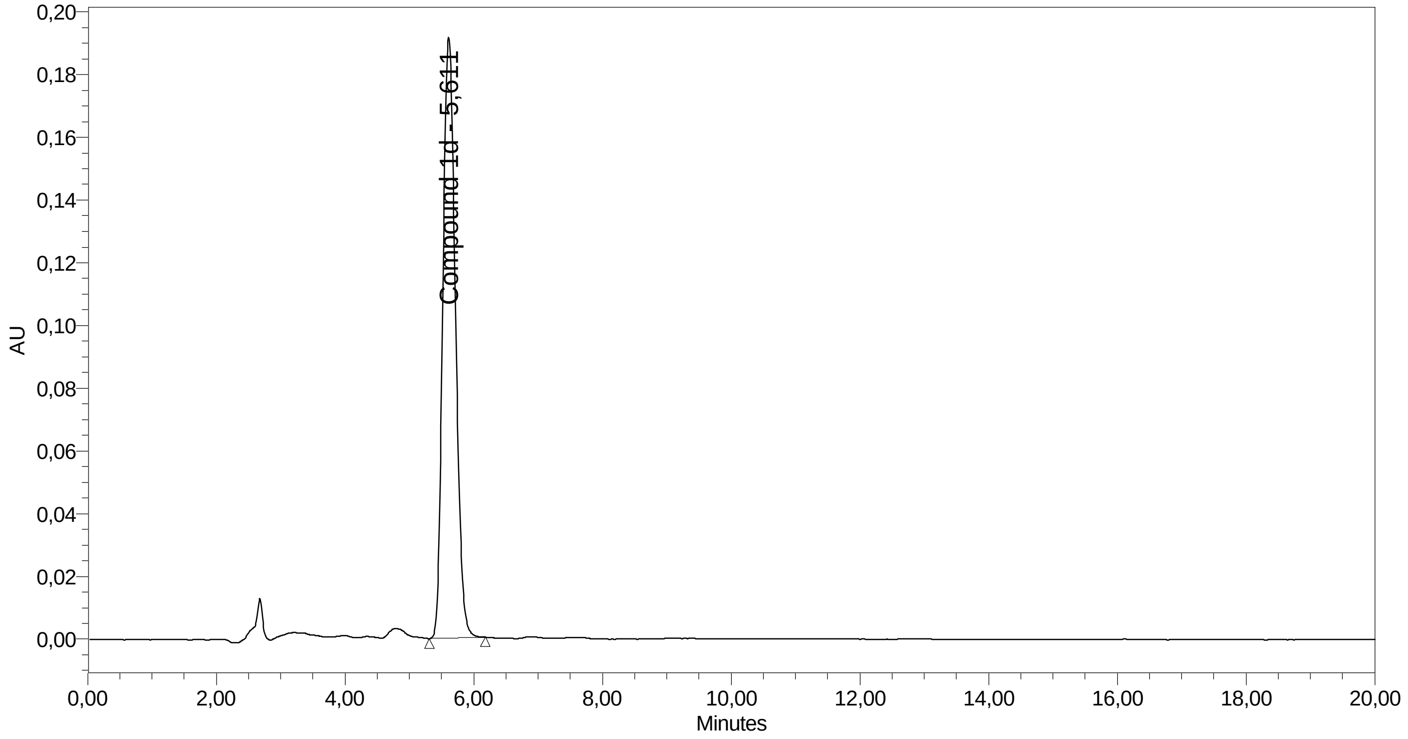

	Peak Name	RT	Area	Height (µV)	Purity1 Angle	Purity1 Threshold	Purity1 Flag
1	Compound 1d	5,611	2607145	191537	0,242	0,287	No

Precision 2_1d_Report

Reported by User: Roxana Roman (Roxana_Roman)
Acquisition Server Waters7
Project Name: Test
Sample Set Name: Precision 2_PyridineM
Code column: Inertsil ODS-3, 4,6*250 mm, 5 um

SAMPLE INFORMATION

Sample Name:	Methyl-pyridine 30 ug/ mL		
Sample Type:	Unknown	Date Acquired:	15.06.2022 18:01:05
Vial:	35	Acq. Method Set:	Precision 2_PyridineM
Injection #:	1	Date Processed:	18.02.2023 09:49:56
Injection Volume:	100,00 ul	Processing Method:	Precision 2_PyridineM
Run Time:	20,0 Minutes	Channel Name:	Extract 275,0
Acquired By:	Roxana_Roman	Proc. Chnl. Descr.:	PDA 275,0 nm, Smoothed by 25

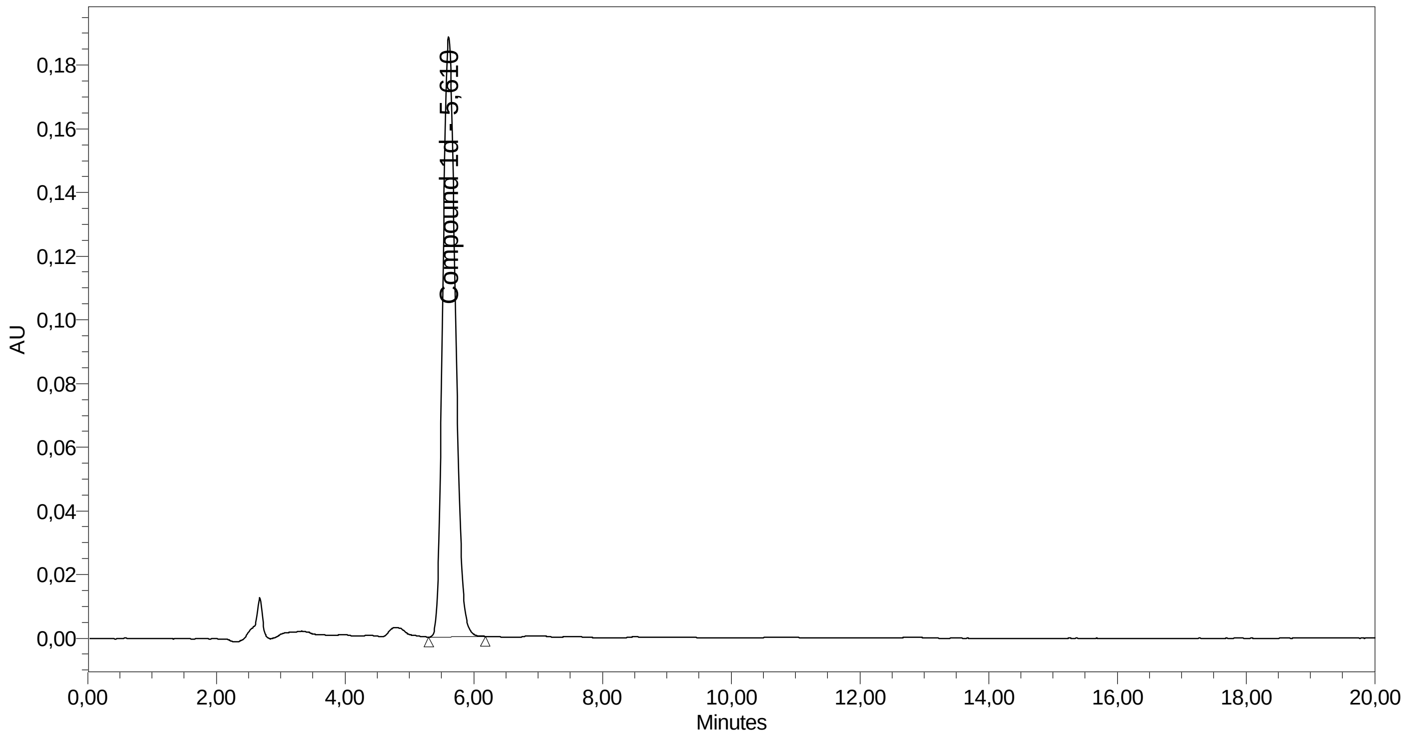

	Peak Name	RT	Area	Height (µV)	Purity1 Angle	Purity1 Threshold	Purity1 Flag
1	Compound 1d	5,610	2564753	188442	0,242	0,294	No

Precision 2_1d_Report

Reported by User: Roxana Roman (Roxana_Roman)
Acquisition Server Waters7
Project Name: Test
Sample Set Name: Precision 2_PyridineM
Code column: Inertsil ODS-3, 4,6*250 mm, 5 um

SAMPLE INFORMATION

Sample Name:	Methyl-pyridine 30 ug/ mL		
Sample Type:	Unknown	Date Acquired:	15.06.2022 18:21:53
Vial:	36	Acq. Method Set:	Precision 2_PyridineM
Injection #:	1	Date Processed:	18.02.2023 09:50:03
Injection Volume:	100,00 ul	Processing Method:	Precision 2_PyridineM
Run Time:	20,0 Minutes	Channel Name:	Extract 275,0
Acquired By:	Roxana_Roman	Proc. Chnl. Descr.:	PDA 275,0 nm, Smoothed by 25

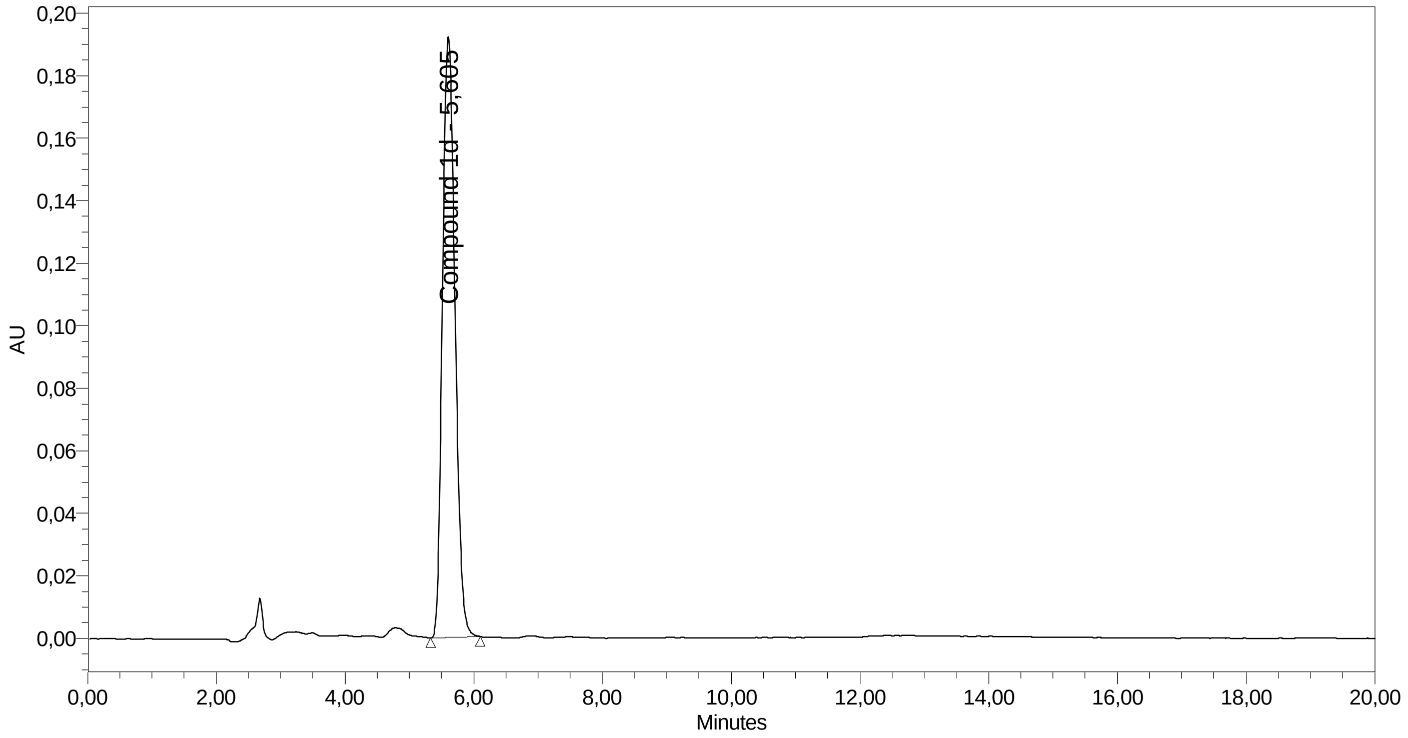

	Peak Name	RT	Area	Height (µV)	Purity1 Angle	Purity1 Threshold	Purity1 Flag
1	Compound 1d	5,605	2603224	192151	0,191	0,284	No

Precision 2_1d_Report

Reported by User: Roxana Roman (Roxana_Roman)
Acquisition Server Waters7
Project Name: Test
Sample Set Name: Precision 2_PyridineM
Code column: Inertsil ODS-3, 4,6*250 mm, 5 um

SAMPLE INFORMATION

Sample Name:	Methyl-pyridine 30 ug/ mL		
Sample Type:	Unknown	Date Acquired:	15.06.2022 21:20:40
Vial:	37	Acq. Method Set:	Precision 2_PyridineM
Injection #:	1	Date Processed:	18.02.2023 09:50:16
Injection Volume:	100,00 ul	Processing Method:	Precision 2_PyridineM
Run Time:	20,0 Minutes	Channel Name:	Extract 275,0
Acquired By:	Roxana_Roman	Proc. Chnl. Descr.:	PDA 275,0 nm, Smoothed by 25

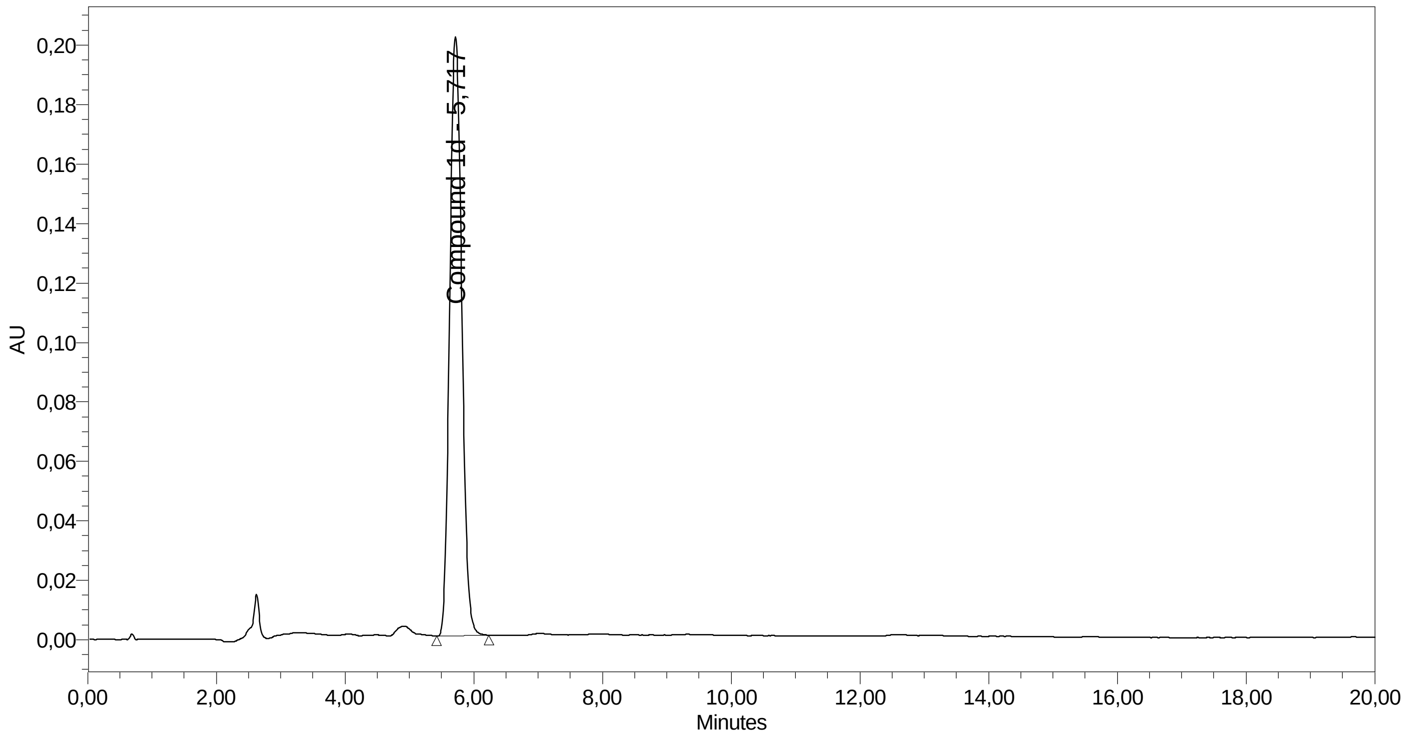

	Peak Name	RT	Area	Height (µV)	Purity1 Angle	Purity1 Threshold	Purity1 Flag
1	Compound 1d	5,717	2617823	201379	0,126	0,578	No

Precision 2_1d_Report

Reported by User: Roxana Roman (Roxana_Roman)
Acquisition Server Waters7
Project Name: Test
Sample Set Name: Precision 2_PyridineM
Code column: Inertsil ODS-3, 4,6*250 mm, 5 um

S A M P L E   I N F O R M A T I O N

Sample Name:	Methyl-pyridine 40 ug/ mL		
Sample Type:	Unknown	Date Acquired:	15.06.2022 19:03:29
Vial:	38	Acq. Method Set:	Precision 2_PyridineM
Injection #:	1	Date Processed:	18.02.2023 09:52:20
Injection Volume:	100,00 ul	Processing Method:	Precision 2_PyridineM
Run Time:	20,0 Minutes	Channel Name:	Extract 275,0
Acquired By:	Roxana_Roman	Proc. Chnl. Descr.:	PDA 275,0 nm, Smoothed by 25

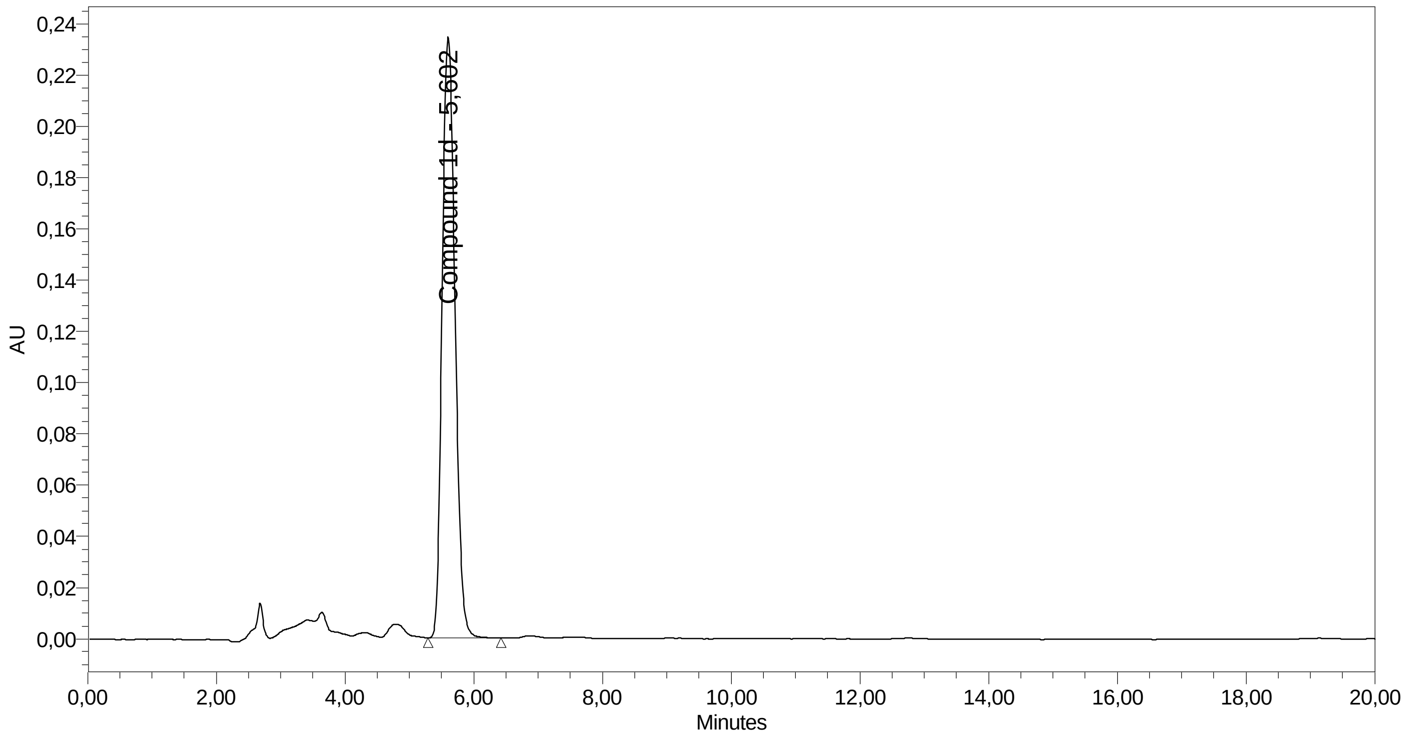

	Peak Name	RT	Area	Height (µV)	Purity1 Angle	Purity1 Threshold	Purity1 Flag
1	Compound 1d	5,602	3259885	234623	0,279	0,294	No

Precision 2_1d_Report

Reported by User: Roxana Roman (Roxana_Roman)
Acquisition Server Waters7
Project Name: Test
Sample Set Name: Precision 2_PyridineM
Code column: Inertsil ODS-3, 4,6*250 mm, 5 um

SAMPLE INFORMATION

Sample Name:	Methyl-pyridine 40 ug/ mL		
Sample Type:	Unknown	Date Acquired:	15.06.2022 19:24:17
Vial:	39	Acq. Method Set:	Precision 2_PyridineM
Injection #:	1	Date Processed:	18.02.2023 09:52:33
Injection Volume:	100,00 ul	Processing Method:	Precision 2_PyridineM
Run Time:	20,0 Minutes	Channel Name:	Extract 275,0
Acquired By:	Roxana_Roman	Proc. Chnl. Descr.:	PDA 275,0 nm, Smoothed by 25

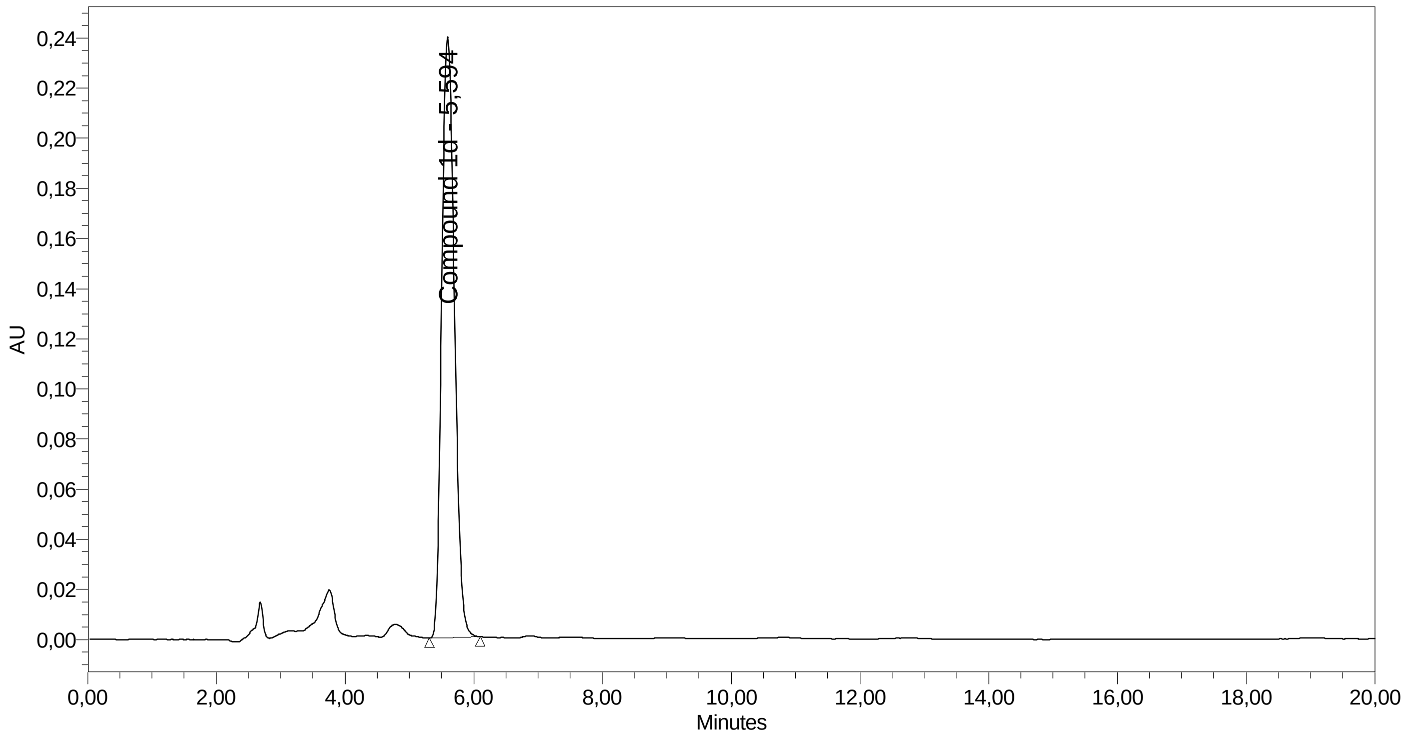

	Peak Name	RT	Area	Height (µV)	Purity1 Angle	Purity1 Threshold	Purity1 Flag
1	Compound 1d	5,594	3293681	239713	0,183	0,289	No

Precision 2_1d_Report

Reported by User: Roxana Roman (Roxana_Roman)
Acquisition Server Waters7
Project Name: Test
Sample Set Name: Precision 2_PyridineM
Code column: Inertsil ODS-3, 4,6*250 mm, 5 um

SAMPLE INFORMATION

Sample Name:	Methyl-pyridine 40 ug/ mL		
Sample Type:	Unknown	Date Acquired:	15.06.2022 19:45:05
Vial:	40	Acq. Method Set:	Precision 2_PyridineM
Injection #:	1	Date Processed:	18.02.2023 09:52:49
Injection Volume:	100,00 ul	Processing Method:	Precision 2_PyridineM
Run Time:	20,0 Minutes	Channel Name:	Extract 275,0
Acquired By:	Roxana_Roman	Proc. Chnl. Descr.:	PDA 275,0 nm, Smoothed by 25

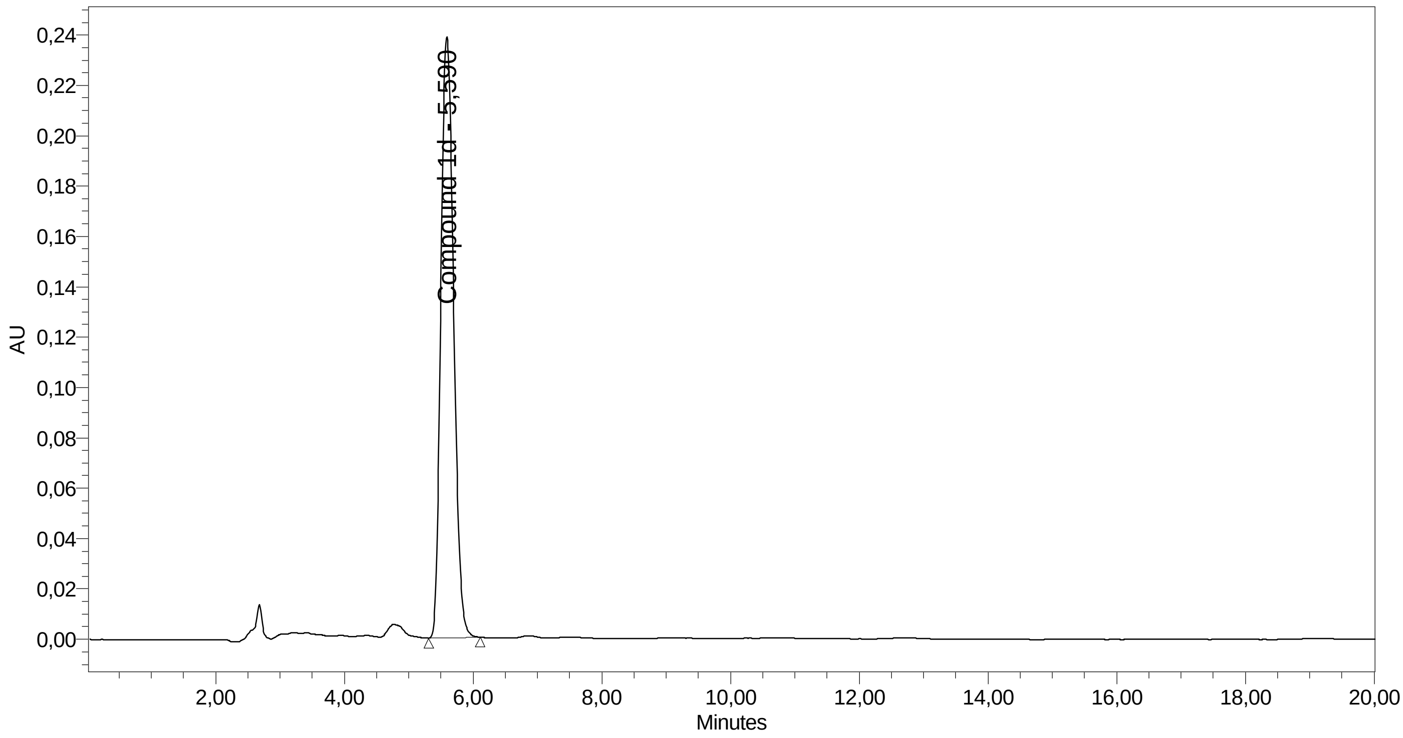

	Peak Name	RT	Area	Height (µV)	Purity1 Angle	Purity1 Threshold	Purity1 Flag
1	Compound 1d	5,590	3297946	238693	0,182	0,287	No

Precision 2_1d_Report

Reported by User: Roxana Roman (Roxana_Roman)
Acquisition Server Waters7
Project Name: Test
Sample Set Name: Precision 2_PyridineM
Code column: Inertsil ODS-3, 4,6*250 mm, 5 um

SAMPLE INFORMATION

Sample Name:	Methyl-pyridine 40 ug/ mL		
Sample Type:	Unknown	Date Acquired:	15.06.2022 20:05:53
Vial:	41	Acq. Method Set:	Precision 2_PyridineM
Injection #:	1	Date Processed:	18.02.2023 09:53:01
Injection Volume:	100,00 ul	Processing Method:	Precision 2_PyridineM
Run Time:	20,0 Minutes	Channel Name:	Extract 275,0
Acquired By:	Roxana_Roman	Proc. Chnl. Descr.:	PDA 275,0 nm, Smoothed by 25

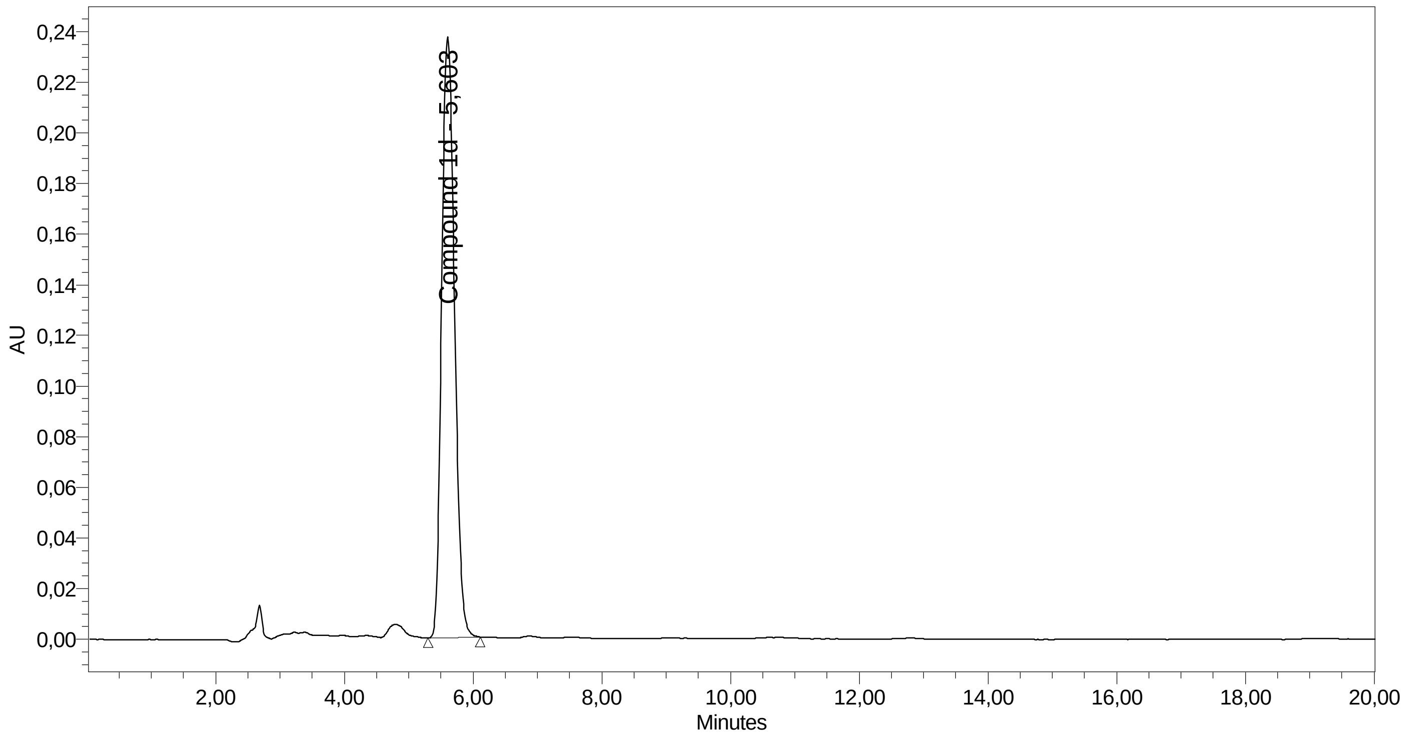

	Peak Name	RT	Area	Height (µV)	Purity1 Angle	Purity1 Threshold	Purity1 Flag
1	Compound 1d	5,603	3291424	237363	0,173	0,287	No

Precision 2_1d_Report

Reported by User: Roxana Roman (Roxana_Roman)
Acquisition Server Waters7
Project Name: Test
Sample Set Name: Precision 2_PyridineM
Code column: Inertsil ODS-3, 4,6*250 mm, 5 um

SAMPLE INFORMATION

Sample Name:	Methyl-pyridine 40 ug/ mL		
Sample Type:	Unknown	Date Acquired:	15.06.2022 20:26:41
Vial:	42	Acq. Method Set:	Precision 2_PyridineM
Injection #:	1	Date Processed:	18.02.2023 09:53:15
Injection Volume:	100,00 ul	Processing Method:	Precision 2_PyridineM
Run Time:	20,0 Minutes	Channel Name:	Extract 275,0
Acquired By:	Roxana_Roman	Proc. Chnl. Descr.:	PDA 275,0 nm, Smoothed by 25

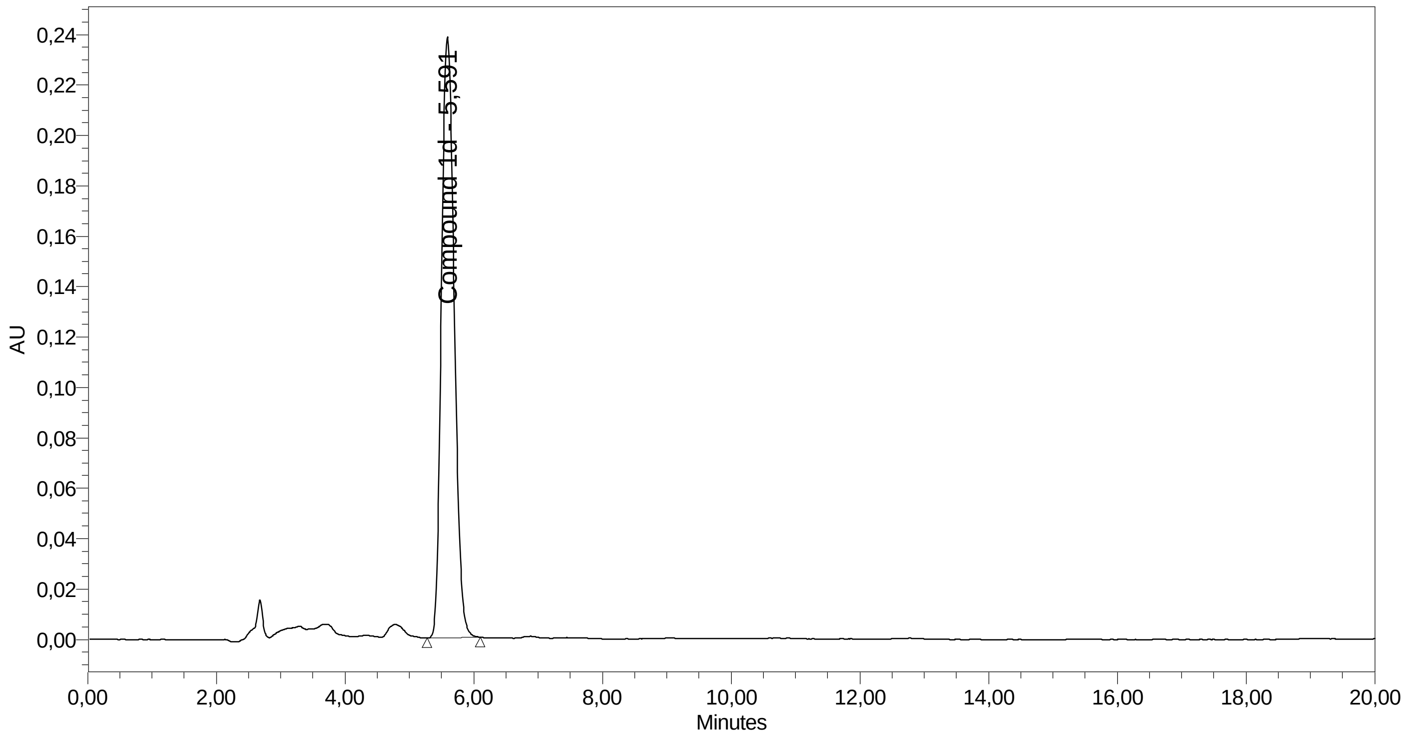

	Peak Name	RT	Area	Height (µV)	Purity1 Angle	Purity1 Threshold	Purity1 Flag
1	Compound 1d	5,591	3297122	238413	0,182	0,287	No

Precision 2_1d_Report

Reported by User: Roxana Roman (Roxana_Roman)
Acquisition Server Waters7
Project Name: Test
Sample Set Name: Precision 2_PyridineM
Code column: Inertsil ODS-3, 4,6*250 mm, 5 um

S A M P L E   I N F O R M A T I O N

Sample Name:	Methyl-pyridine 40 ug/ mL		
Sample Type:	Unknown	Date Acquired:	15.06.2022 20:47:29
Vial:	43	Acq. Method Set:	Precision 2_PyridineM
Injection #:	1	Date Processed:	18.02.2023 09:53:33
Injection Volume:	100,00 ul	Processing Method:	Precision 2_PyridineM
Run Time:	20,0 Minutes	Channel Name:	Extract 275,0
Acquired By:	Roxana_Roman	Proc. Chnl. Descr.:	PDA 275,0 nm, Smoothed by 25

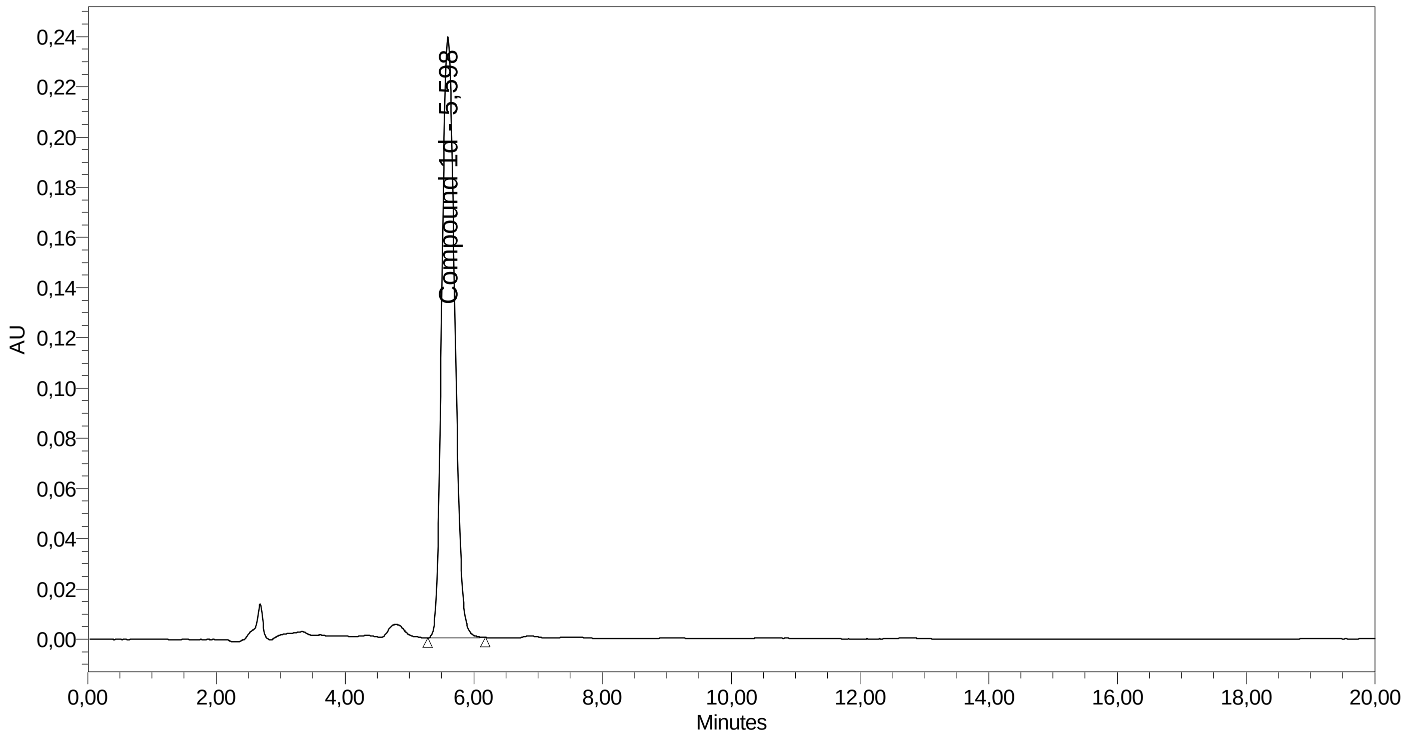

	Peak Name	RT	Area	Height (µV)	Purity1 Angle	Purity1 Threshold	Purity1 Flag
1	Compound 1d	5,598	3328312	239372	0,245	0,290	No
